# Transcriptome Analysis Reveals the Role of Trehalose in Response to Polyethylene Terephthalate Nanoplastics Treatment in Foxtail Millet ( *Setaria italica* ) Seedlings

**DOI:** 10.1002/fsn3.70593

**Published:** 2025-07-21

**Authors:** Shan Du, Yue Guo, Manabu Wen‐Liu Tanimura, Jiaqi Bai, Jie Zheng, Liwen Liu, Pu Yang, Lizhen Zhang, Ben Zhang

**Affiliations:** ^1^ School of Life Science Shanxi University Taiyuan Shanxi China; ^2^ Shanxi Key Laboratory for Research and Development of Regional Plants Shanxi University Taiyuan Shanxi Province China; ^3^ Key Laboratory of Chemical Biology and Molecular Engineering of Ministry of Education Taiyuan Shanxi China; ^4^ Graduate School of Human and Environmental Studies Kyoto University Sakyo Kyoto Japan; ^5^ Seed Bank Co. Ltd. Sakyo Kyoto Japan

**Keywords:** PET nanoplastics, ROS, *Setaria italica*, transcriptome, trehalose

## Abstract

Foxtail millet ( *Setaria italica* ), a drought‐tolerant C4 model plant, faces increasing threats from polyethylene terephthalate (PET) nanoplastics in agricultural ecosystems. While prior studies indicate that PET nanoplastics induce reactive oxygen species (ROS) accumulation and impair crop productivity, the physiological mechanisms underlying plant responses remain unclear. This study investigates the role of trehalose metabolism in mitigating PET nanoplastic stress in foxtail millet. Transcriptome sequencing of seedlings treated with 1 g/L PET nanoplastics (3 and 7 days) revealed significant differential expression of genes linked to trehalose accumulation, hormone signaling, and metabolic pathways. Notably, genes associated with trehalose biosynthesis (*SiTPS*/*SiTPP*) and degradation (*SiTRE*) were dynamically regulated, suggesting trehalose homeostasis as a critical stress–response mechanism. Exogenous trehalose application effectively alleviated ROS damage under nanoplastic treatment, corroborating its protective role. Further WGCNA analysis indicated the potential involvement of ABA signal transduction and the MAPK signaling pathway in foxtail millet's response to PET nanoplastic stress. Additionally, our findings build on earlier observations that elevated leaf potassium content mitigates ROS but further highlight trehalose‐mediated signaling as a complementary adaptive strategy. These results demonstrate that trehalose metabolism, ABA signal transduction, MAPK signaling pathway, and alongside ion homeostasis are integral to foxtail millet's resilience to PET nanoplastics, offering novel insights into plant stress adaptation and potential strategies for enhancing crop tolerance in contaminated environments.

## Graphical abstract

Transcriptome sequencing of seedlings treated with 1 g/L PET nanoplastics (3 and 7 days) revealed genes associated with trehalose biosynthesis (*SiTPS*/*SiTPP*) and degradation (*SiTRE*) were dynamically regulated, suggesting trehalose homeostasis as a critical stress‐response mechanism. (2) Exogenous trehalose application effectively alleviated ROS damage under nanoplastic treatment, corroborating its protective role. (3) Further WGCNA analysis indicated the potential involvement of ABA signal transduction and the MAPK signaling pathway in foxtail millet's response to PET nanoplastic stress.

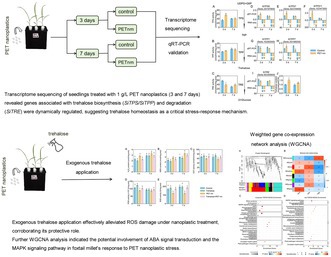

## 1 Introduction

Foxtail millet ( *Setaria italica* ), which originated in China and is now cultivated globally, is a high‐dietary‐fiber whole‐grain food that is rich in vitamins and proteins (Ceasar 2023; He et al. 2015; Yang, Ma, et al. 2022). Due to its drought and barren environment tolerance, small genome size, and the recent advancements in genetic transformation techniques, foxtail millet has become an ideal model organism for exploring C4 plant genetic resources and researching stress‐related response mechanisms (Peng and Zhang 2021).

Along with the development of human industrial civilization, many plastic products have been discharged into the natural environment, resulting in “white pollution” that threatens animals and plants (Liu et al. 2025; Qi et al. 2020). Polyethylene terephthalate (PET), a thermoplastic polymer originating from petroleum, is highly esteemed for its durability, remarkable transparency, and lightweight nature. These characteristics have led to its extensive utilization across a multitude of global applications, encompassing agricultural films, packaging materials, and fiber manufacturing (Dhaka et al. 2022). Similar to other plastic products, PET may undergo degradation into fragments when exposed to varying physical and chemical environments, resulting in the formation of microplastics measuring less than 5 mm and nanoplastics with dimensions smaller than 1 mm (Banerjee and Shelver 2021; He et al. 2018). Due to their minute size, large surface area, and resistance to biodegradation, these plastic particles can easily accumulate in organisms, including plants, and impact plant growth (Rillig et al. 2019). In the foxtail millet‐producing regions of northern China, including Shanxi Province, the Inner Mongolia Autonomous Region, and Hebei Province, microplastics have been detected in farmland with mean abundances of 1300–1600 items per kg, approximately 2–3 times higher than those found in the arid northwestern regions. Notably, nanoplastics, including PET, have been detected in the farmland (Chen et al. 2022). Recent studies have indicated that PET nanoplastic pollution could affect agricultural crop productivity. Research reveals that exposure to PET micro/nanoplastics can cause a rapid increase in reactive oxygen species (ROS), accumulation of heavy metals, impaired seed germination, and reduced biomass accumulation in plants (Abbasi et al. 2020; Mondal et al. 2022; Pignattelli et al. 2021).

Trehalose, a non‐reducing disaccharide composed of two glucose molecules by an α,α‐1,1‐glycosidic bond, was initially discovered in a parasitic mycelium fungus and later found to be ubiquitous across most organisms, including plants (Kosar et al. 2019). In plants, trehalose biosynthesis is facilitated by a singular pathway involving two key enzymes: trehalose‐6‐phosphate (T6P) synthase (TPS, EC 2.4.1.15) and trehalose‐6‐phosphate phosphatase (TPP, EC 3.1.3.12). TPS facilitates the conversion of UDP‐glucose and glucose‐6‐phosphate into T6P, which subsequently undergoes dephosphorylation by TPP to produce trehalose (Avonce et al. 2006; Eh et al. 2024; Kosar et al. 2019). Trehalose plays a pivotal role in protecting biomolecules under extreme conditions, such as drought and freezing, by forming hydrogen bonds with surrounding biopolymers and biomembranes (Fernandez et al. 2010). Recent studies have also highlighted the function of trehalose and its precursor, T6P, as integral signaling molecules. The exogenous application of trehalose has been shown to enhance plant tolerance to various abiotic stresses, including drought, salinity, heat, and cold (Eh et al. 2024; Griffiths et al. 2016). This protective mechanism is linked to the mitigation of reactive oxygen species (ROS) bursts and associated damage (Liu et al. 2021; Mostofa, Hossain, and Fujita 2015; Yang, Yao, et al. 2022). However, it is crucial to note that excessive trehalose can be detrimental to plants (Luo et al. 2021; Schluepmann et al. 2004). To maintain trehalose levels within a beneficial range, plant cells utilize trehalase (TRE, EC 3.2.1.28) to catalyze the degradation of trehalose (Fernandez et al. 2010; Müller et al. 2001).

Our previous study demonstrated that short‐term exposure to 1 g/L PET nanoplastics did not significantly affect the germination and seedling growth of foxtail millet, although it did result in ROS accumulation. Additionally, the treatment led to an elevation in the expression levels of genes related to leaf potassium absorption and transport within a 3‐day period, thereby enhancing leaf potassium content. Furthermore, we discovered that high leaf potassium content mitigated ROS damage induced by PET nanoplastic treatment (Guo et al. 2024). However, apart from the rise in potassium content, it is still unclear whether other physiological processes are involved in the response of foxtail millet to PET nanoplastic treatment.

To further elucidate the physiological response mechanisms of foxtail millet under PET nanoplastic treatment, we performed transcriptome sequencing on plants subjected to 1 g/L PET nanoplastics for durations of 3 and 7 days, using untreated controls as references. This analysis disclosed changes in the expression levels of numerous genes, particularly those engaged in trehalose metabolism and hormone signal transduction. Follow‐up investigations have demonstrated that trehalose accumulation is crucial for foxtail millet's response to PET nanoplastic treatment. Additionally, the external application of trehalose has been proven to efficiently mitigate ROS damage caused by PET nanoplastic treatment.

## 2 Materials and Methods

### 2.1 Plant Growth Conditions and Treatment

Seeds of the foxtail millet cultivar “Jingu21” were obtained from Professor Lizhen Zhang's laboratory at Shanxi University, China. The PET nanoplastics were acquired from Zhangmutou Suyuan Plastic Material Co. Ltd., located in Dongguan, China. These particles exhibited a spherical structure with a rough surface and had an average size of approximately 790 nm, accompanied by a zeta potential of −18.5 ± 0.1 mV (Guo et al. 2024).

The cultivation of foxtail millet was conducted following previously established protocols (Fan et al. 2024; Guo et al. 2023; Liang et al. 2024; Zhang, Guo, et al. 2022). Seeds were germinated and grown in trays containing a 1:1 (v/v) mixture of vermiculite and nutrient soil. The greenhouse environment was maintained under a 16‐h light/8‐h dark cycle at 26°C/23°C, with a light intensity of 50,000 Lux and relative humidity ranging from 30% to 50%. The soil used had a pH of 6.0 ± 0.1 (in H_2_O). At the two‐leaf stage, uniform seedlings were selected and transplanted into plastic pots, with three plants per pot. After 1 week, these seedlings were irrigated with a solution containing 1 g/L PET nanoplastics, corresponding to a 0.03% w/w plastic/soil ratio. For the control group, leaves from “Jingu21” seedlings that were watered instead of PET nanoplastic solution were collected. To evaluate the impact of PET on foxtail millet in liquid culture, 10‐day‐old “Jingu21” seedlings were transferred to a 1/2 MS liquid medium with a pH of 5.8 and grown for an additional 4 days. Subsequently, they were exposed to a 1/2 MS solution containing 1 g/L PET nanoplastics. To investigate the role of trehalose, based on previous research (Mostofa, Hossain, and Fujita 2015; Yang, Yao, et al. 2022), the seedlings were treated with trehalose (10 mmol/L) 2 days before the PET nanoplastic application. Each experimental condition was replicated three times. The leaves were then harvested, frozen in liquid nitrogen, and stored at −80°C.

### 2.2 RNA Isolation and Library Preparation

Total RNA was isolated utilizing the TRIzol reagent (Invitrogen, CA, USA) adhering to the protocols provided by the manufacturer. The RNA yield was quantified with a NanoDrop 2000 spectrophotometer (Thermo Scientific, USA), and the integrity of RNA was assessed via the Agilent 2100 Bioanalyzer (Agilent Technologies, Santa Clara, CA, USA). The RNA quality assessment revealed that the OD260/280 ratios fell within the range of 2.0–2.1, and the RIN values were all greater than 8. Thereafter, libraries were constructed using the VAHTS Universal V6 RNA‐seq Library Prep Kit in compliance with the manufacturer's instructions. Transcriptome sequencing and subsequent analysis were conducted by OE Biotech Co. Ltd. (Shanghai, China).

### 2.3 RNA Sequencing and Differentially Expressed Genes Analysis

Library sequencing was carried out on an Illumina Novaseq 6000 platform, generating 150 bp paired‐end reads. The raw fastq‐formatted reads were first processed with fastp (Chen et al. 2018) to eliminate low‐quality reads and obtain clean reads. These clean reads were then aligned against the *Setaria italica* v2.2 transcript from the JGI transcriptome reference database (https://phytozome‐next.jgi.doe.gov/) using HISAT2 (Kim et al. 2015). Gene expression levels were quantified by FPKM values (Roberts et al. 2011), with read counts derived from HTSeq (Anders et al. 2015). Significantly differentially expressed genes (DEGs) were determined using a *p* < 0.05 and fold change > 2 or < 0.5 as the threshold. R (v 3.2.0) was utilized for hierarchical cluster analysis. Based on the hypergeometric distribution, Gene Ontology (GO) and Kyoto Encyclopedia of Genes and Genomes (KEGG) pathway enrichment analyses of DEGs were conducted using R (v 3.2.0) to identify significantly enriched terms.

### 2.4 qRT‐PCR Analysis

Quantitative real‐time PCR (qRT‐PCR) was conducted following previously described methods (Fan et al. 2024; Guo et al. 2024; Liang et al. 2024). Total RNA from foxtail millet leaves was extracted using the TransZol UP Plus RNA Kit (TransGen Biotech, Beijing, China). The A260/A230 ratio was between 2.0 and 2.1, indicating good RNA quality. Subsequently, the RNA was reverse‐transcribed using the EasyScript One‐Step gDNA Removal and cDNA Synthesis SuperMix Kit (TransGen Biotech, Beijing, China). The qPCR was performed with the Perfect‐Start Green qPCR SuperMix kit (TransGen Biotech, Beijing, China), and the data were analyzed by the 2^−*ΔΔCT*^ method (Schmittgen and Livak 2008). *SiAct2* (*Seita.8G043100*) and *SiRNA POL II* (*Seita.2G142700*) were used as internal reference genes. All primers employed in the experiments were synthesized by Sangon Biotech, Shanghai (Table S1). Each experiment included three technical replicates and three biological replicates.

### 2.5 Physiology Analysis

The leaf samples of “Jingu21” seedlings were harvested as described above. The malondialdehyde (MDA) content (MDA‐2‐Y), hydrogen peroxide (H_2_O_2_) content (H_2_O_2_‐2‐Y), catalase (CAT) activity (CAT‐2‐Y), peroxidase (POD) activity (POD‐2‐Y), and superoxide dismutase (SOD) activity (SOD‐2‐Y) were measured using assay kits obtained from Suzhou Keming Biotechnology Co. Ltd. (China). The activities of trehalose‐6‐phosphate synthase (TPS) and trehalase (TRE) were determined using kits purchased from Suzhou Michy Biomedical (China). The standard curve provided by the kit is used (Table S11), and all sample detection signal values were within the linear range of the curve. Trehalose content was measured by Qidian Chemical Technology Service (Liaocheng, China). The tissue samples were prepared according to a previous report (Kretzschmar et al. 2015). Analyses of trehalose by an Agilent 1100‐Thermos TSQ Quantum Ultra AM liquid chromatography–tandem mass spectrometry were carried out as described (Hayner et al. 2017; Kretschmer et al. 2016). The testing standards of trehalose were acquired from Shanghai Yuanye Bio‐Technology (China).

### 2.6 Weighted Gene Coexpression Network Analysis (WGCNA)

Construct a gene co‐expression network module using R (v 1.1.25). Filter genes with low fluctuations in expression levels (standard deviation ≤ 0.5), obtain co‐expression modules through automatic network construction and module detection functions, and use a soft threshold power of 30 and *p* < 0.05 as the threshold. Similar modules are fused using a combined cutting height of 0.25. Use the Pearson correlation algorithm to calculate the correlation coefficient (*r*) and *p* value between module feature genes and traits, and select modules related to each trait based on the threshold of |*r*| > 0.3 and *p* < 0.05. Draw a heatmap of the correlation between trait modules and perform KEGG enrichment analysis on key modules.

### 2.7 Statistical Analysis

The statistical analysis of independent experiments is presented as means ± standard error. Significance was evaluated using Student's *t*‐test or ANOVA.

## 3 Results

### 3.1 Transcriptome Sequence and Differential Expression Analysis of Genes in Foxtail Millet Under PET Nanoplastics Treatment

Previous studies have demonstrated that exposing foxtail millet seedlings to 1 g/L PET nanoplastics does not significantly affect their growth. However, it induces ROS accumulation in leaves (Guo et al. 2024; Figure S1). To delve into the physiological implications at the genetic expression level, we conducted transcriptome sequencing and analysis on foxtail millet leaves following a 3‐day and 7‐day exposure to 1 g/L PET nanoplastics, using unexposed plants as controls.

Each experimental condition was replicated three times across biological samples, resulting in a total of 12 cDNA libraries. These were divided into two groups: the control group (designated as TC3d and TC7d) and the treated group (designated as TS3d and TS7d). Transcriptome sequencing was performed on these 12 samples, and after removing adapter sequences and low‐quality reads, a total of 82.13 Gb of clean data was obtained. The average number of clean reads is 47.06 million. For each sample, the effective data volume ranged from 5.98 to 7.05 Gb, with Q30 base percentages between 91.64% and 94.57%, and an average GC content of 52.6%. The sequencing was performed using paired‐end sequencing, with a read length of 150 bp. The clean reads were mapped against the reference genome using HISAT2, achieving alignment rates per sample between 94.51% and 95.96%. The proportion of unique mapped reads for each sample is 91.79% or higher (Table S2). A significant distinction was observed between the control and treatment groups, accompanied by consistent correlations among the three biological replicates within each group (Figure S2), confirming the suitability of the transcriptome data for subsequent differential gene expression analysis.

**FIGURE 1:**
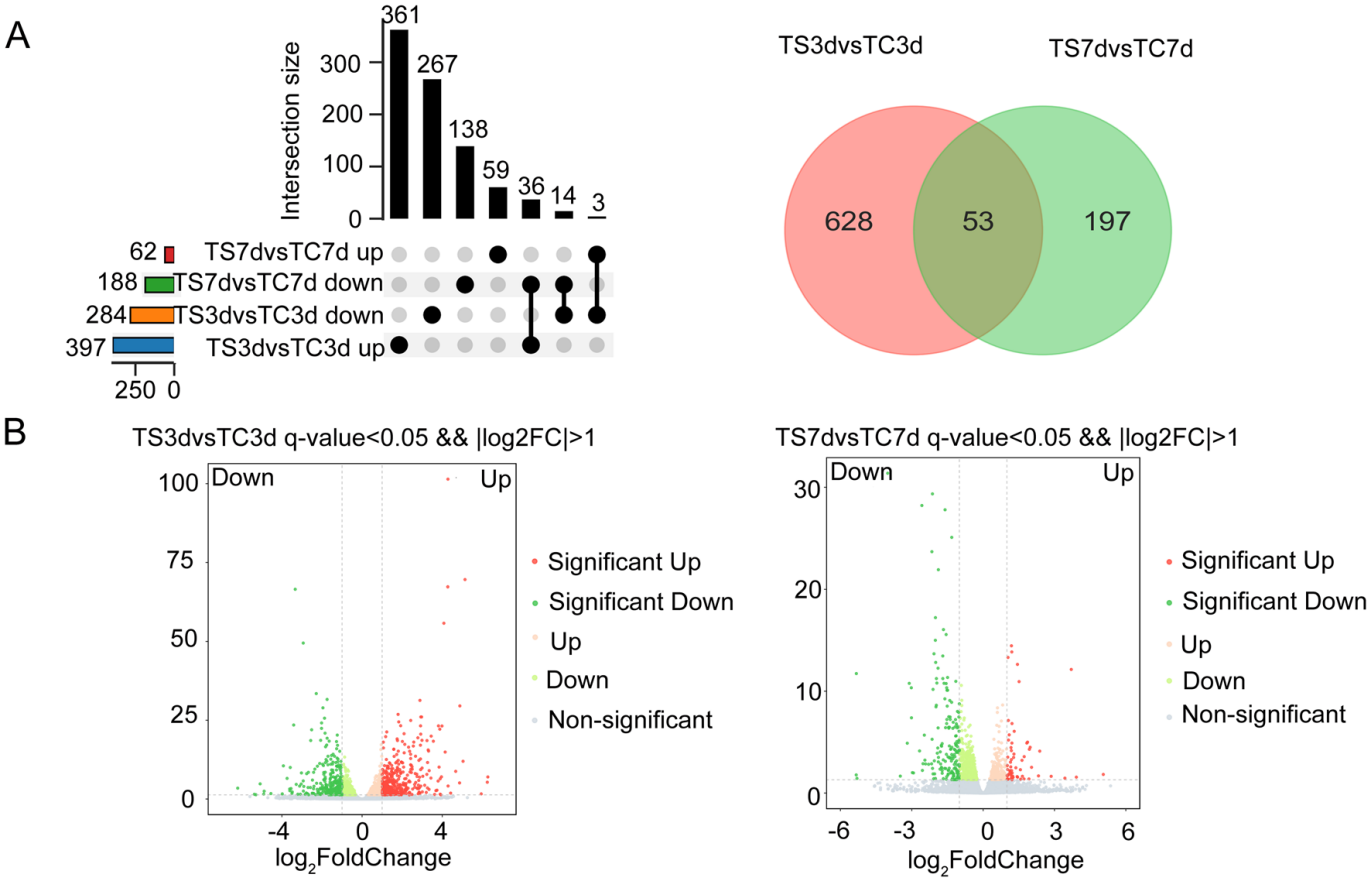
Statistics of the number of differentially expressed genes. (A) Upset plots and Venn diagrams illustrate the distribution of upregulated and downregulated differentially expressed genes in foxtail millet after 3 and 7 days of PET nanoplastic treatment, as well as the number of unique and overlapping DEGs between different time points. (B) Volcanic map of differentially expressed genes in foxtail millet after 3 and 7 days of PET nanoplastic treatment. Gray is a nonsignificant difference gene, red and green are significant difference genes; the horizontal axis is log_2_ FoldChange, and the vertical axis is −log_10_ *q*‐value.

Using DESeq2 software, pairwise comparisons were conducted between the control and treatment groups (TS3d vs. TC3d and TS7d vs. TC7d). The differential expression analysis revealed 681 DEGs after 3 days of treatment relative to the control, comprising 397 upregulated and 284 downregulated genes. In contrast, only 250 DEGs were identified after 7 days of treatment, including 62 upregulated and 188 downregulated genes. Notably, the comparison of TS3d versus TC3d exhibited a substantially higher number of DEGs compared to TS7d versus TC7d, with 58.30% of genes upregulated at 3 days—a proportion notably exceeding the 24.80% observed at 7 days. This indicates more pronounced transcriptional adjustments in foxtail millet under PET nanoplastic stress at the earlier time point. Further examination of the DEGs between the two time points and Venn diagram analysis uncovered 628 unique DEGs in TS3d versus TC3d and 197 specific DEGs in TS7d versus TC7d. Among the 53 overlapping DEGs, 14 displayed consistent downregulation, suggesting their potential significance in the prolonged response of foxtail millet to nanoplastic exposure (Figure 1, Tables S3 and S4).

### 3.2 Differential Gene Function Enrichment Analysis

**FIGURE 2:**
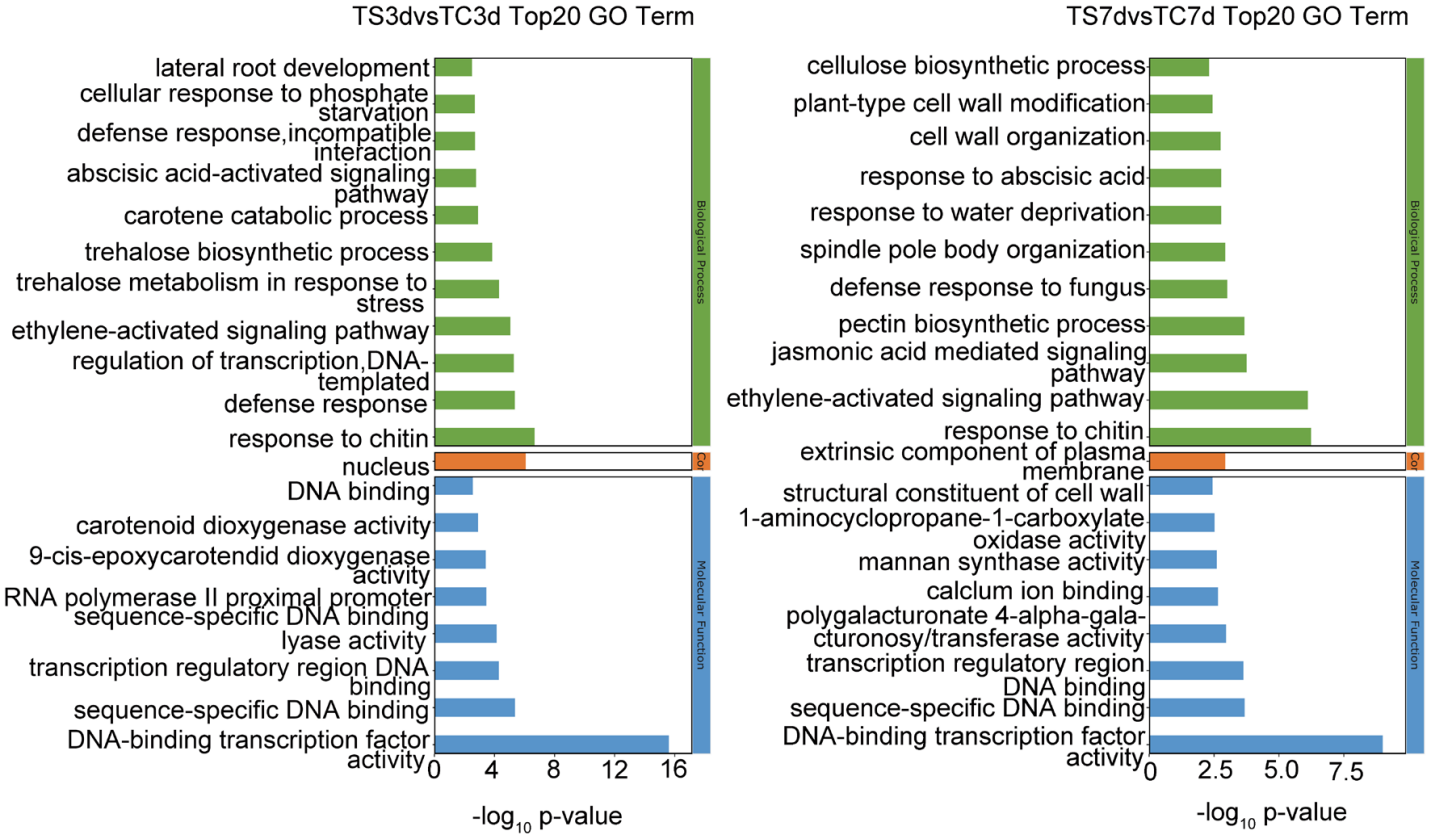
The top 20 GO terms of differentially expressed genes in “Jingu21” seedlings exposed to 1 g/L PET nanoplastics for 3 and 7 days. The vertical axis represents the GO term names, and the horizontal axis represents the −log_10_ *p* value. Three colors represent the GO classification. Green refers to the biological process (BP), orange refers to the cellular component (CC), and blue refers to the molecular function (MF).

Gene Ontology (GO) and Kyoto Encyclopedia of Genes and Genomes (KEGG) enrichment analyses were conducted on the identified sets of DEGs. According to GO annotation (Figure 2, Tables S5 and S6), DEGs from both groups showed significant enrichment in biological processes and molecular functions. The analysis revealed that after a 3‐day exposure to PET nanoplastic stress, foxtail millet markedly activated regulatory networks associated with trehalose metabolic processes (e.g., trehalose metabolism in response to stress, GO: 0070413; trehalose biosynthetic process, GO: 0005992), defense responses (e.g., response to chitin, GO: 0010200; defense response, GO: 0006952; defense response to incompatible interaction, GO: 0009814), plant hormone signaling (e.g., abscisic acid‐activated signaling pathway, GO: 0009738), and other factors. Following a 7‐day exposure, foxtail millet notably activated networks linked to plant hormone signaling (e.g., jasmonic acid‐mediated signaling pathway, GO: 0009867; response to abscisic acid, GO: 0009737) and cell wall structure (e.g., pectin biosynthetic process, GO: 0045489; cellulose biosynthetic process, GO: 0030244; plant‐type cell wall modification, GO: 0009827; cell wall organization, GO: 0071555; structural constituent of cell wall, GO: 0005199). At both 3 and 7 days under PET nanoplastic treatment, foxtail millet significantly activated regulatory networks related to plant hormone signaling (e.g., ethylene‐activated signaling pathway, GO: 0009873).

**FIGURE 3:**
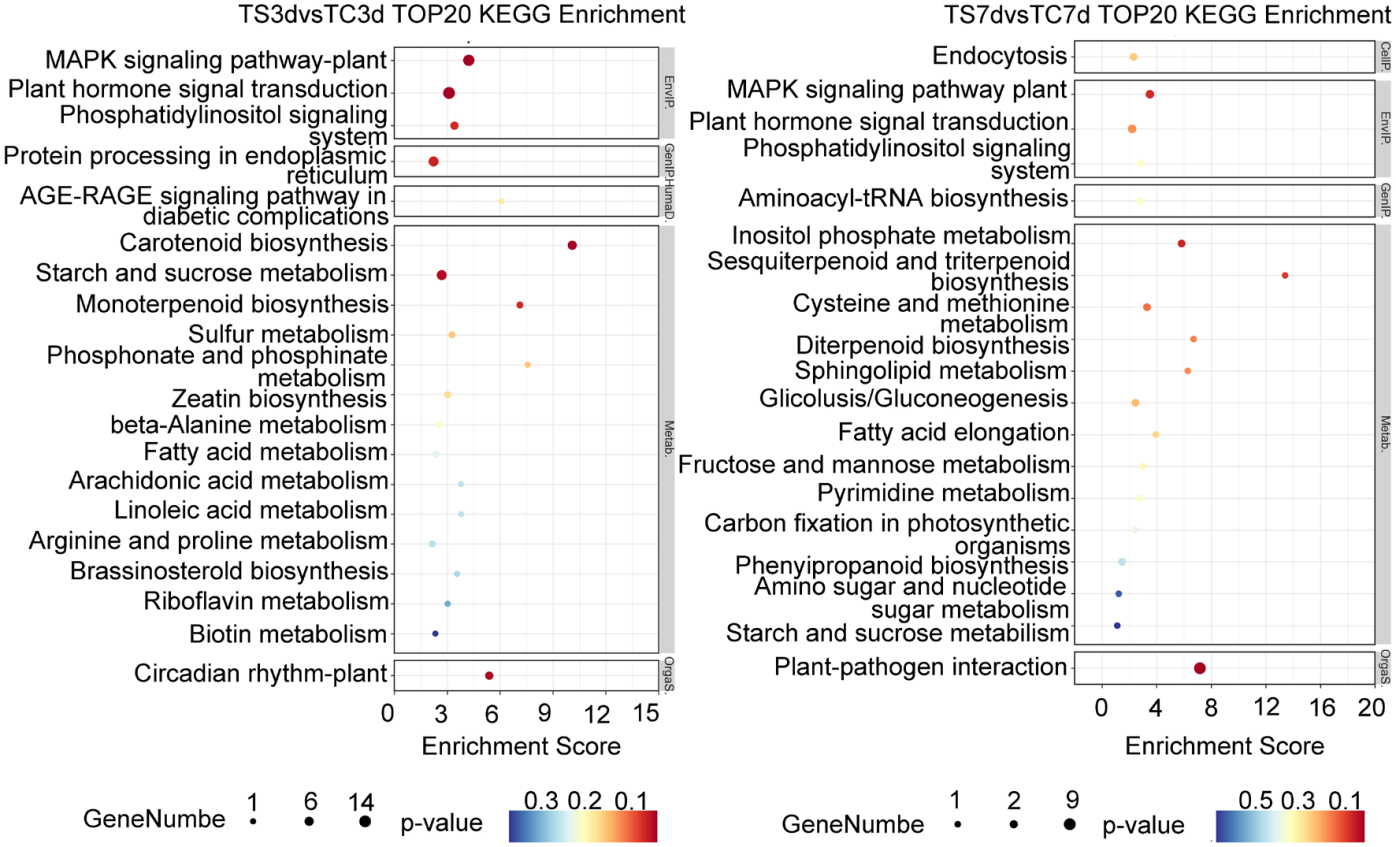
The top 20 KEGG enrichment of differentially expressed genes in “Jingu21” seedlings exposed to 1 g/L PET nanoplastics for 3 and 7 days. Pathway entries with PopHits ≥ 5 were filtered and sorted in descending order based on their −log_10_ *p* value. The horizontal axis represents the enrichment score. The size of the bubbles corresponds to the number of differentially expressed protein‐coding genes, with larger bubbles indicating more genes. The color gradient of the bubbles ranges from blue to white, yellow, and red, reflecting the significance of enrichment, where a smaller *p* value indicates greater significance.

In the KEGG pathway enrichment analysis (Figure 3, Tables S7 and S8), 681 DEGs (from TS3d vs. TC3d) and 250 DEGs (from TS7d vs. TC7d) were mapped to 54 and 19 pathways, respectively. These pathways were categorized into five main processes: metabolism, genetic information processing, environmental information processing, cellular processes, and organismal systems. The DEGs from the 3‐day treatment group were significantly enriched in pathways such as the MAPK signaling pathway in plants (ko04016), plant hormone signal transduction (ko04075), carotenoid biosynthesis (ko00906), starch and sucrose metabolism (ko00500), and circadian rhythm in plants (ko04712). Conversely, the DEGs from the 7‐day treatment group displayed significant enrichment in plant–pathogen interaction (ko04626) and other pathways. These findings imply that distinct regulatory mechanisms are engaged in foxtail millet seedlings when subjected to PET nanoplastic stress at varying time points.

**FIGURE 4:**
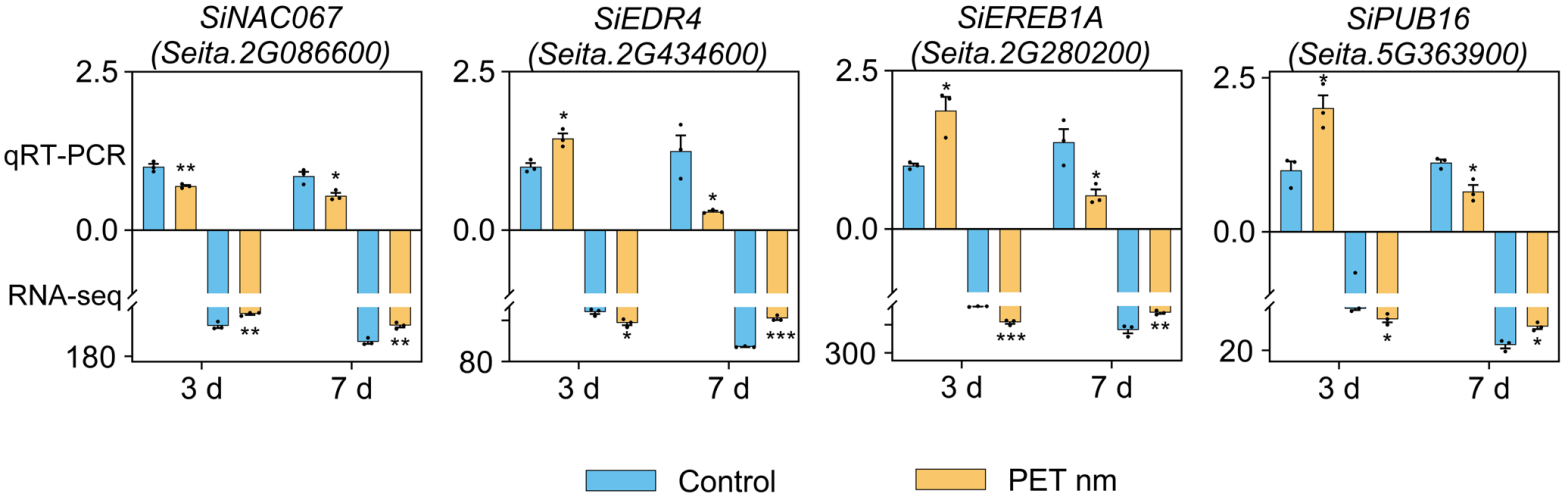
Verification of RNA sequencing results by quantitative real‐time polymerase chain reaction (qRT‐PCR). *SiAct2* (*Seita.8G043100*) and *SiRNA POL II* (*Seita.2G142700*) were used as internal references. In each gene, the transcription level of the 3‐day control was designated as 1, while the expression levels of other samples were transformed. These values are expressed as mean ± SE (*n* = 3) and * show significant differences compared to the respective control seedlings, *p* < 0.05.

To validate the transcriptomic outcomes concerning the DEGs, we randomly selected four genes for qRT‐PCR analysis. The gene expression patterns deduced from qRT‐PCR aligned seamlessly with those observed in the transcriptomic study (Figure 4).

### 3.3 The Effect of PET Nanoplastics Treatment on Trehalose Metabolism in Foxtail Millet Seedlings

Trehalose, a non‐reducing sugar, plays a crucial role in osmotic regulation and stress responses in plants (Eh et al. 2024; Hassan et al. 2023; Yang, Yao, et al. 2022). Our transcriptome analysis indicates that exposure to PET nanoplastics influences the expression of a suite of genes associated with trehalose metabolism (Figures 2 and 3), highlighting a potential involvement of trehalose metabolic pathways in the plant's stress response to this pollutant. To further elucidate this mechanism, we examined both the transcriptional levels and enzymatic activities of pivotal enzymes implicated in trehalose biosynthesis and degradation under PET nanoplastic stress conditions.

**FIGURE 5:**
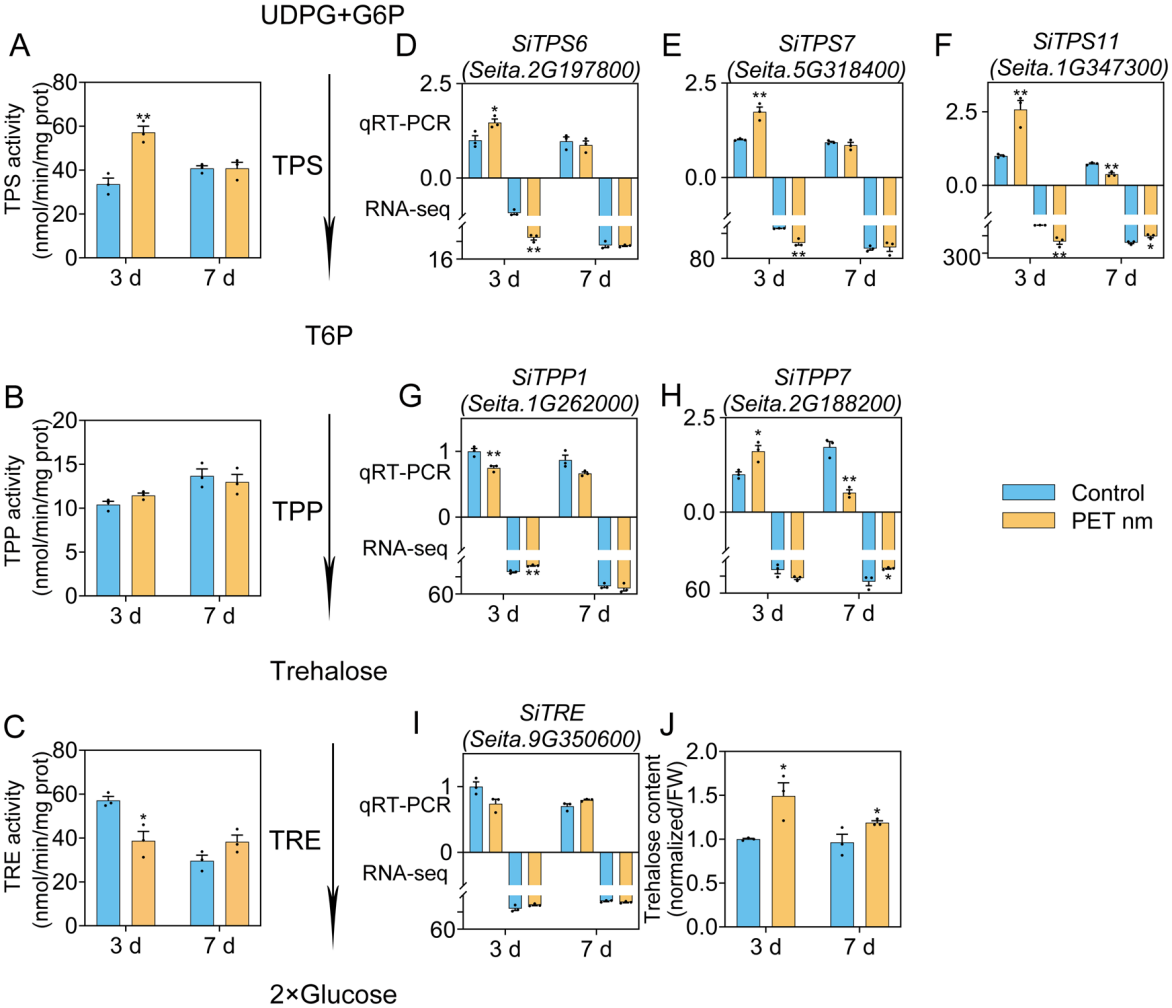
The effect of exogenous trehalose application on the trehalose metabolism pathway in leaves of “Jingu21” foxtail millet. Two‐week‐old “Jingu21” foxtail millet seedlings were hydroponically cultured and subsequently treated with 1 g/L PET nanoplastics. Leaves were harvested at 3 and 7 days posttreatment to measure the activities of TPS, TPP, and TRE. *SiAct2* (*Seita.8G043100*) and *SiRNA POL II* (*Seita.2G142700*) were used as internal references. In each gene, the transcription level of the 3 days control was designated as 1, while the expression levels of other samples were transformed. These values are expressed as mean ± SE (*n* = 3) and * show significant differences compared to the respective control seedlings, *p* < 0.05. (A–C) Represent the enzymatic activities of TPS, TPP, and TRE, respectively. (D–I) The expression levels of *SiTPS6* (*Seita.2G197800*), *SiTPS7* (*Seita.5G318400*), *SiTPS11* (*Seita.1G347300*), *SiTPP1* (*Seita.1G262000*), *SiTPP7* (*Seita.2G188200*), and *SiTRE* (*Seita.9G350600*) are represented, respectively. (J) The relative content of trehalose. The trehalose level was measured using LC–MS, normalized per fresh weight, and expressed relative to control samples on Day 3. For each treatment and time point, the assays were carried out on three independent biological replicates.

As depicted in Figure 5, our transcriptome analysis disclosed an upregulation of key trehalose synthesis genes, specifically those from the *TPS* and *TPP* gene families (Avonce et al. 2006; Eh et al. 2024), following a 3‐day exposure to PET nanoplastics. However, these gene expressions returned to baseline levels by Day 7 posttreatment, with some even showing a slight reduction. These findings were corroborated through qRT‐PCR analyses conducted on foxtail millet samples. Corresponding enzymatic assessments in leaf tissues revealed a concurrent increase in TPS activity after 3 days, which normalized by day 7. Conversely, despite changes in gene transcription profiles, TPP enzymatic activities exhibited no significant variation throughout the experimental period. TRE, an enzyme integral to trehalose catabolism (Eh et al. 2024), displayed a transient decrease in its gene expression 3 days postnanoplastic exposure, subsequently recovering to control values by Day 7. This downturn was mirrored in the measured TRE enzymatic activities, suggesting a coordinated response at both genetic and functional levels. Collectively, these perturbations in gene expression and enzymatic functions resulted in elevated trehalose concentrations within plant tissues subjected to PET nanoplastic treatment.

### 3.4 Exogenous Trehalose Alleviates the Damage Caused by PET Nanoplastic Treatment, Represented by ROS Burst on Foxtail Millet Seedlings

**FIGURE 6:**
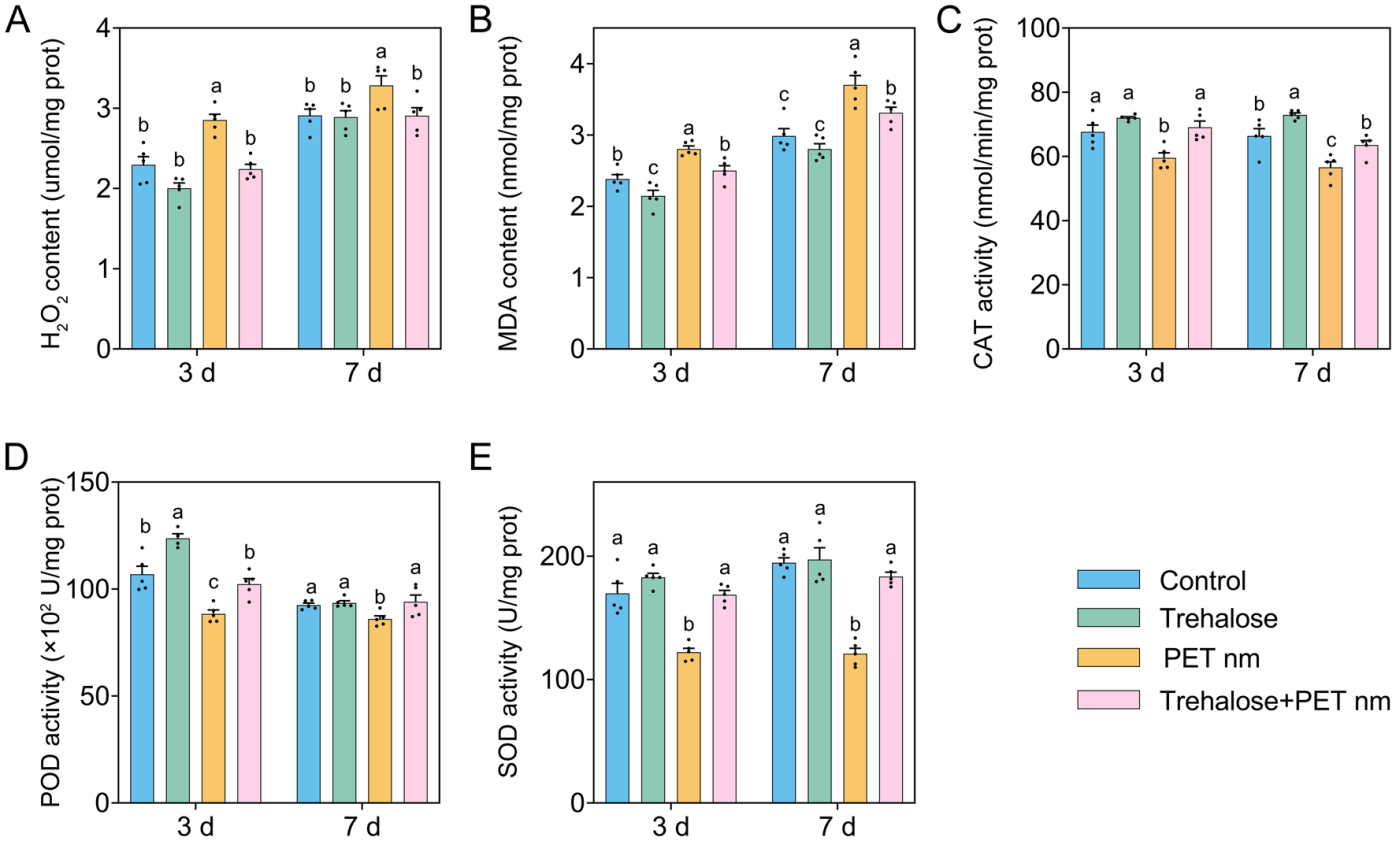
Exogenous trehalose alleviates the damage caused by PET nanoplastic treatment. The “Jingu21” foxtail millet seedlings were hydroponically cultured for 2 weeks and then pretreated with 10 mmol/L trehalose for 2 days prior to exposure to 1 g/L PET nanoplastics in 1/2 MS solution. Leaf samples were collected at 3 and 7 days posttreatment for analysis of ROS‐related physiological parameters and antioxidant enzyme activities. Values show mean ± SE (*n* = 5). Means with different letters differ significantly (*p* < 0.05) according to Duncan's multiple range test following ANOVA. (A–E) Represent the H_2_O_2_ content, MDA content, CAT activity, POD activity, and SOD activity, respectively.

Trehalose plays a role in the plant's response to various biotic and abiotic stresses (Eh et al. 2024). Treatment with PET nanoplastics leads to the accumulation of trehalose in foxtail millet seedlings. To further verify the function of trehalose in this response, we externally applied trehalose and examined the plant's response, represented by an oxidative burst, under PET nanoplastic treatment. As shown in Figure 6, trehalose treatment alone did not result in a significant increase in the level of ROS. The treatment with PET nanoplastics inhibited the activities of antioxidant enzymes such as CAT, SOD, and POD. This led to an increase in the levels of ROS, represented by H_2_O_2_, in the leaves of foxtail millet. Consequently, there was an elevation in MDA levels, indicating a certain degree of membrane damage. These findings are consistent with previous reports (Guo et al. 2024). The exogenous application of trehalose alleviates the ROS damage represented by the above‐mentioned indicators, bringing the plants in line with the control group.

### 3.5 Identification of Coexpression Modules Related to Trehalose Metabolism Pathway Using WGCNA

**FIGURE 7:**
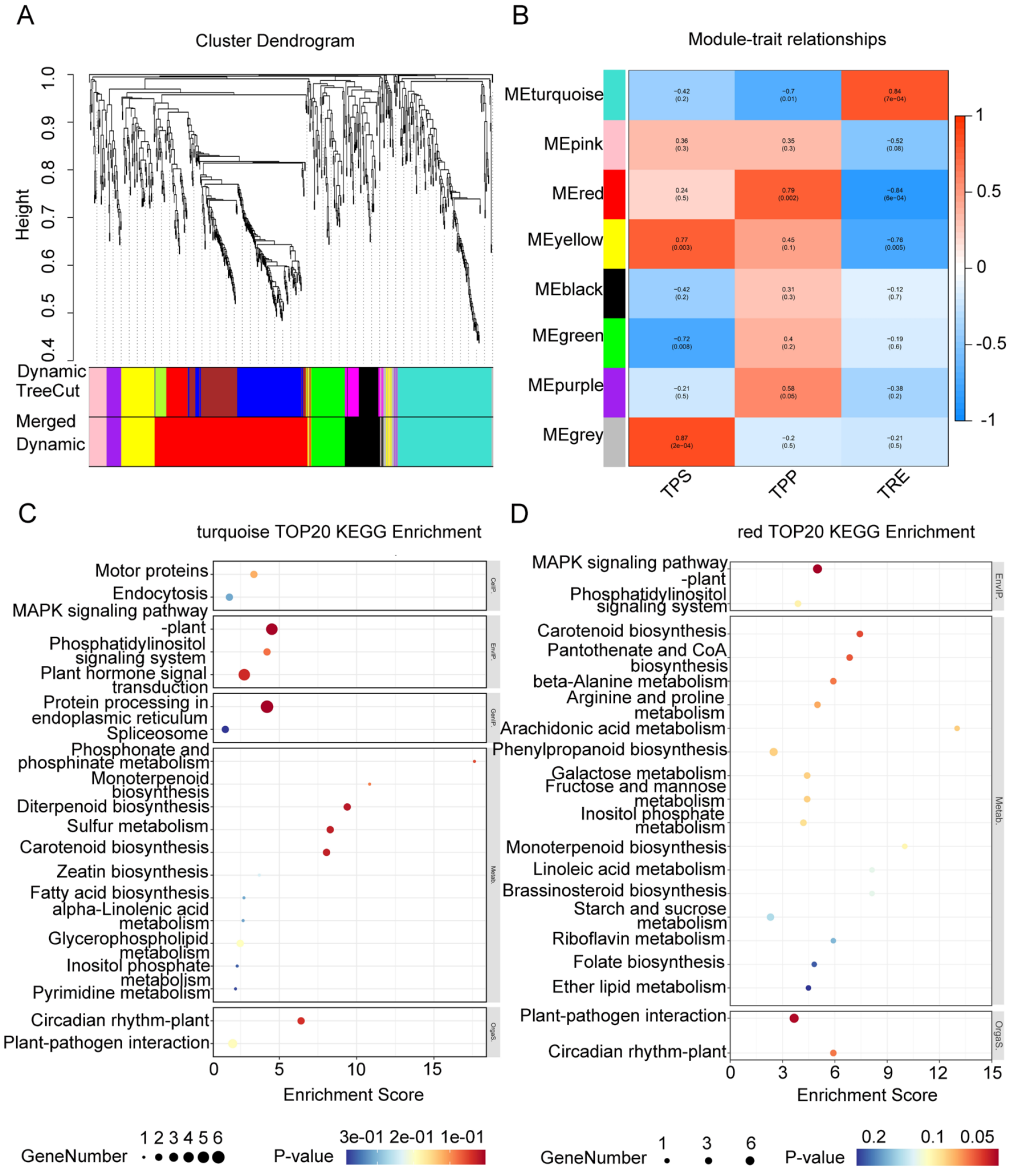
Weighted gene co‐expression network analysis (WGCNA). (A) Hierarchical clustering diagram of the modules. The upper part of the figure is the gene clustering tree constructed by the dissTOM matrix based on the weighted correlation coefficients. The lower part of the figure shows the distribution of genes within each module. Genes assigned the same color belong to the same module, as indicated in the “Dynamic Tree Cut” panel, which was generated using the Dynamic Tree Cut method. Due to correlations between certain modules, some were merged into unified modules, as shown in the “Merged Dynamic” panel below. These merged modules represent the final set used for subsequent analysis. (B) Characteristic module correlation heatmap. Red denotes a positive correlation, while blue represents a negative correlation. The color intensity reflects the strength of the correlation, with darker shades reflecting stronger correlations. The numbers in parentheses are *p* values, with smaller values indicating higher statistical significance. (C) KEGG pathway analysis of the unique DEGs in the turquoise module. (D) KEGG pathway analysis of the unique DEGs in the red module.

To identify gene modules co‐expressed with the trehalose metabolism pathway, we analyzed all DEGs (878 total) identified 3 and 7 days after PET nanoplastic treatment. Genes showing low expression fluctuation (standard deviation ≤ 0.5) were filtered out, retaining 724 genes for weighted gene co‐expression network analysis (WGCNA). This analysis grouped the 724 genes into eight distinct modules (Figure 7A). The gray module contains genes not assigned to any coexpression group and was excluded from further analysis.

Subsequently, we assessed module correlations using module eigengenes (Figure 7B). The red module was the largest (276 genes), while the gray module was the smallest (18 genes). Module‐trait correlation analysis revealed that the turquoise module showed a significant negative correlation with TPP activity (*r* = −0.7, *p* = 0.01) and a significant positive correlation with TRE activity (*r* = 0.84, *p* = 7e‐04). The red module showed a significant positive correlation with TPP activity (*r* = 0.79, *p* = 0.002) and a significant negative correlation with TRE activity (*r* = −0.84, *p* = 5e‐04). The yellow module showed a significant positive correlation with TPS activity (*r* = 0.77, *p* = 0.003) and a significant negative correlation with TRE activity (*r* = −0.76, *p* = 0.005).

As shown in Figure 7B, the expression pattern of the red module paralleled the trehalose metabolic pathway, while the turquoise module exhibited the opposite pattern. Then, KEGG pathway enrichment analysis was performed to elucidate potential functions of module DEGs. The results showed that genes in the turquoise module were significantly enriched in “Plant hormone signal transduction” and the “MAPK signaling pathway” (Figure 7C, Table S9), while genes in the red module were significantly enriched in the “MAPK signaling pathway” and “Plant‐pathogen interaction” (Figure 7D, Table S10). Further analysis found that the red module contained two genes related to ABA signal transduction, one gene related to catalase, and one transcription factor of *WRKY* family. The turquoise module contained three genes related to ABA signal transduction, one *MAPKKK* family gene, and one *ZIP* family gene. These findings suggest that ABA signal transduction, the MAPK signaling pathway, and trehalose metabolism may be involved in foxtail millet's response to PET nanoplastic stress.

## 4 Discussion

In recent years, nanoplastic pollution has been shown to impact plant growth, posing a threat to agricultural production and food security. Foxtail millet, a drought‐tolerant C4 crop, also has its planting areas contaminated by nanoplastics, including PET (Chen et al. 2022). Here, we conducted transcriptome analysis on foxtail millet leaves after 3 and 7 days of treatment with 1 g/L PET nanoplastics to screen for DEGs and explore the response mechanism of foxtail millet to PET nanoplastic treatment. Our findings indicate that: (1) PET nanoplastic treatment for 3 days affected the expression of genes related to trehalose accumulation, defense responses, and hormone signaling, whereas treatment for 7 days predominantly affected the expression of genes related to hormone signaling. Further WGCNA analysis revealed the potential involvement of ABA signal transduction and MAPK signaling pathway in foxtail millet's response to PET nanoplastic stress. (2) The exogenous application of trehalose mitigated the effects of PET nanoplastic treatment on plants by inhibiting the accumulation of ROS.

### 4.1 ABA Signal Transduction and MAPK Signaling Pathway Are Involved in the Response of Foxtail Millet Seedlings to PET Nanoplastic Treatment

In our previous research, we observed that PET nanoplastic treatment did not significantly influence the growth of foxtail millet seedlings (Guo et al. 2024). However, during a 7‐day exposure period, alterations in the expression levels of genes associated with potassium ion absorption and transport were evident, resulting in potassium accumulation within foxtail millet leaves. Besides the elevation in potassium ion content, it remains unclear whether additional physiological mechanisms contribute to the foxtail millet's response to PET nanoplastic treatment. Notably, the previously reported changes in gene expression related to potassium ion absorption and transportation peaked around Day 3 and returned to baseline levels by Day 7 (Guo et al. 2024). To further explore the underlying molecular responses, we therefore performed transcriptome sequencing at both 3 and 7 days posttreatment, using untreated millet plants as controls.

Plant hormones, as important signaling molecules, play a crucial role in regulating stress responses to biotic and abiotic stresses (Das et al. 2025; Han et al. 2023; Waadt et al. 2022). Through transcriptome analysis (Figures 2 and 3) and WGCNA analysis (Figure 7), it was found that ABA signal transduction and MAPK signaling pathway may play a key role in the response of foxtail millet to PET nanoplastic treatment. In addition, the data indicate that PET nanoplastic treatment affected the expression levels of other plant hormone‐related genes at two time points. Based on previous studies, PYR/PYL family proteins function as receptors for abscisic acid (ABA) and regulate ABA signaling by inhibiting PP2C family proteins (Park et al. 2009). Foxtail millet responds to PET nanoplastic treatment by modulating the expression levels of genes involved in the ABA hormone‐related PYL‐PP2C signaling pathway. After 3 days of PET nanoplastic treatment, the ABA signaling pathway genes *SiPYL4* (*Seita.9G437300*) and *SiPYL5* (*Seita.3G076200*) were induced, whereas the expression of *SiPP2C8* (*Seita.3G139000*), *SiPP2C9* (*Seita.5G379400*), *SiPP2C49* (*Seita.3G218800*), and *SiPP2C68* (*Seita.2G177500*) was suppressed. Similar changes in the expression levels of genes involved in these signaling pathways have been demonstrated to participate in plant responses to other stresses. Overexpressing AtPYL4 and AtPYL5 in Arabidopsis significantly improves drought resistance, boosts antioxidant enzyme activity, and promotes the accumulation of osmolyte regulatory substances (Shi et al. 2014). Overexpression of *TaPYL4* positively modulates stomatal movement, stimulates osmolyte biosynthesis, and improves drought adaptation in wheat (Zhang, Zhao, et al. 2022). The *PP2C* gene plays a pivotal switch role in the ABA signaling pathway, functioning as a negative regulator of ABA signals (Jung et al. 2020). The upregulation or suppression of *PP2C* family gene expression serves distinct functions in the plant's response to abiotic stress. In *AtMYB44* transcription factor overexpressing Arabidopsis, the expression of *PP2C* family gene is suppressed, enhancing the plant's sensitivity to ABA, accelerating stomatal closure, and thereby increasing tolerance to drought stress (Jung et al. 2010, 2008). Similar results have also been observed in rice and soybean (Joo et al. 2017; Seo et al. 2012). In our transcriptome analysis, we also found that the transcription factor *SiMYB44* (*Seita.2G140900*), homologous to *AtMYB44* in Arabidopsis, was upregulated under PET nanoplastic treatment. This finding explains why the *SiPP2C* genes were downregulated under the same treatment condition.

As the core regulatory network of plant stress response, the MAPK signaling pathway can integrate multiple environmental signals and is crucial for maintaining plant physiological homeostasis (Sheikh et al. 2013; Van Gerrewey and Chung 2024). Under PET nanoplastic treatment, *SiPYL4/5* and *SiPP2C8/9/49* are involved in the perception and negative regulation of ABA signals, while *SiMAPKKK17*, as an upstream kinase of the MAPK cascade, may integrate ABA signals into the MAPK network through phosphorylation modification. This finding is consistent with existing research that in hybrid rice, MAPKKK17 mediates the ABA signaling pathway, thereby enhancing plant drought resistance (Cui et al. 2024); in Arabidopsis, its homologous gene *AtMAPKKK17* has been confirmed to be a key kinase at the ABA‐MAPK signaling crossover node, coordinating plant stress resistance and development (Tajdel‐Zielińska et al. 2024).

It is worth noting that the interaction mechanism between trehalose accumulation and ABA and MAPK signaling pathways still needs to be further elucidated. Previous studies have shown that exogenous trehalose treatment can regulate ABA signaling, but its mode of action does not directly affect ABA metabolism. It may enhance ABA sensitivity by upregulating the expression of *PYL4/PYL5* receptor genes (Yu et al. 2019). A similar phenomenon has also been validated in tea plants, where trehalose enhances plant heat tolerance by regulating the ABA signaling pathway, inhibiting the expression of *MAPKKK17/18* (Zheng et al. 2024). However, the core issues of how trehalose accumulation perceives environmental stress signals and how to precisely regulate the specific mechanisms of key components in these two signaling pathways remain unclear, which will also be an important direction for future research.

In addition, PET nanoplastic treatment also regulated changes in the expression levels of other hormone‐related genes. Under 7 days of PET nanoplastic stress, the expression of the WRKY transcription factors, including *SiWRKY24* (*Seita.3G206900*), *SiWRKY50* (S*eita.3G108500*), *SiWRKY51* (*Seita.3G164900*), and *SiWRKY71* (*Seita.1G062100*), was significantly downregulated. Previous studies have demonstrated that certain WRKY transcription factors are regulated by the Jasmonic acid (JA) signaling pathway. For instance, in Arabidopsis, *AtWRKY50* and *AtWRKY51* negatively regulate the JA signaling pathway, and their inhibitory effects on JA are independent of salicylic acid (Gao et al. 2011). The overexpression of *SlWRKY50* enhances cold stress tolerance in *Solanum lycopersicum* and plays a crucial role in JA biosynthesis (Wang et al. 2024). Under PET nanoplastic stress for 3 and 7 days, the expression of the key ethylene (ET) biosynthesis enzymes *SiACO1* (*Seita.2G225500*) and *SiACO5* (*Seita.3G037900*) was induced. The 1‐aminocyclopropane‐1‐carboxylic acid oxidase (ACO) catalyzes the oxidation of 1‐aminocyclopropane‐1‐carboxylic acid to produce ET, which represents the final and crucial step in ET biosynthesis (Wang et al. 2025). ET is closely related to plant growth, development, and environmental adaptation (Han et al. 2023; Zhao et al. 2024). In rice varieties with different sensitivities to microplastic stress, the expression of ET‐responsive transcription factors varies under microplastic treatment, suggesting a physiological role for ethylene in responding to microplastic stress (Wu et al. 2022). However, further experimental exploration is needed to investigate the dynamic changes in endogenous hormone levels and the potential mechanisms and reaction processes of PET nanoplastic treatment.

### 4.2 Foxtail Millet Alleviates ROS Damage by Accumulating Trehalose as a Response to PET Nanoplastic Stress

Our transcriptomic analysis demonstrated that a 3‐day exposure to PET nanoplastic treatment resulted in the upregulation of genes associated with trehalose synthesis (Figures 2 and 5). As a nonreducing disaccharide composed of two glucose molecules connected via an a,a‐1,1‐glycosidic bond, trehalose exhibits unique chemical properties. In plants experiencing stress, the levels of trehalose undergo significant fluctuations, serving as an osmoprotectant and thereby augmenting the plant's resistance to biotic and abiotic stresses (Eh et al. 2024). Within plants, trehalose is synthesized via the TPS‐TPP pathway and degraded by TRE (Avonce et al. 2006; Fernandez et al. 2010; Kosar et al. 2019).

As illustrated in Figure 5, following 3 days of PET nanoplastics treatment, there was an increase in the transcriptional levels of *SiTPS6*, *SiTPS7*, and *SiTPS11*, accompanied by heightened TPS activity in leaves, suggesting an augmented conversion of G6P to T6P. Concurrently, a decrease in TRE expression was observed, leading to reduced TRE activity in leaves. These two phenomena acted in concert, ultimately resulting in elevated trehalose content within the leaves. Given that previous research has implicated trehalose in plant responses to abiotic stress (Eh et al. 2024; Yang, Yao, et al. 2022), we postulate that foxtail millet accumulates trehalose as a mechanism to counteract the stress induced by PET nanoplastics.

The results of the DEGs analysis also prompted us to investigate the transcriptional levels of *SiTPP*, another pivotal enzyme implicated in trehalose biosynthesis. While the expression profiles of *SiTPP1* and *SiTPP7* exhibited alterations under PET nanoplastics exposure, the aggregate enzymatic activity of TPP in leaves did not markedly change. This could be attributed to our analysis not encompassing the entire *SiTPP* gene family in foxtail millet, potentially overlooking gene expression modifications that were not captured in the transcriptomic data. A more exhaustive systematic examination and quantification of the *SiTPP* gene family under stress conditions would be instrumental in addressing this knowledge gap.

Moreover, these findings imply that the bottleneck for trehalose accumulation during the experimental stress period was primarily the TPS‐mediated conversion of G6P to T6P. Extensive research has established trehalose and its precursor T6P as vital signaling molecules in plants, orchestrating a myriad of physiological processes (Eh et al. 2024; Griffiths et al. 2016; Morales‐Herrera et al. 2023; Ponnu et al. 2011). T6P has the potential to stimulate growth in conditions where the carbon supply is sufficient; however, this growth can be impeded when the elevation of T6P levels is not proportional to the available carbon resources (O'Hara et al. 2013). T6P inhibits SnRK1 activity, thereby affecting the *bZIP11* promoter, which is usually associated with changes in sucrose content (Tsai and Gazzarrini 2014). Moreover, Arabidopsis transgenic lines overexpressing *TRE* display an abnormal phenotype, also suggesting the signaling function of T6P (Nuñez‐Muñoz et al. 2021). Building on these insights, probing how PET nanoplastic stress influences T6P levels and the subsequent role of T6P in foxtail millet's adaptive response merits further inquiry.

Evidence also suggests that the exogenous application of trehalose enhances plant tolerance against drought, salinity, heat, and cold stress by suppressing ROS production (Eh et al. 2024; Griffiths et al. 2016). Our previous study revealed that PET nanoplastics disturb antioxidant enzyme functions in foxtail millet, leading to ROS overproduction (Guo et al. 2024). Consequently, we investigated the trehalose mitigating effect on PET‐induced oxidative stress. As shown in Figure 6, the synergistic administration of trehalose and PET effectively reduced ROS overaccumulation, providing protective benefits to plants.

Collectively, these data imply that foxtail millet counteracts PET nanoplastics‐triggered ROS bursts through trehalose accumulation, thereby alleviating resultant damage. Notably, our earlier work demonstrated that, simultaneously, foxtail millet increases leaf potassium content to mitigate ROS damage (Guo et al. 2024). The potential interplay between these two defensive mechanisms warrants further elucidation. In addition to suppressing ROS bursts, reports have shown that the exogenous addition of trehalose can also alleviate the effects of salt stress on plants by affecting ion balance. In *Catharanthus roseus* , exogenous trehalose reduces Na^+^ accumulation, increases K^+^ content, and raises the K^+^/Na^+^ ratio (Chang et al. 2014). Similar phenomena have also been reported in tomato, where changes in the expression levels of ion transporter genes such as *SlSOS1*, *SlNHX*, and *SlHKT1.1* under salt stress are influenced by trehalose (Yang, Yao, et al. 2022). Whether trehalose contributes to ion homeostasis in plants under PET nanoplastic stress via modulation of ion channels and transporters remains an avenue for future exploration.

## 5 Conclusion

In summary, the transcriptomic analysis revealed that exposure to PET nanoplastics stress caused alterations in the transcriptional profiles of genes related to various physiological processes in foxtail millet. Notably, genes associated with trehalose metabolism, including *SiTPS* and *SiTRE*, also showed transcriptional changes under this stress condition, leading to increased trehalose levels in leaves. The experiment using exogenous trehalose supplementation demonstrated that foxtail millet reduces oxidative stress‐induced damage from PET nanoplastic exposure by accumulating trehalose. In addition, further WGCNA analysis revealed the potential involvement of ABA signal transduction and MAPK signaling pathway in foxtail millet's response to PET nanoplastic stress. This study not only deepens our understanding of the molecular mechanisms underlying foxtail millet's response to nanoplastic stress but also suggests a potential role for trehalose in enhancing plant resilience against nanoplastic‐induced stress.

## Author Contributions

**Shan Du:** data curation (equal), formal analysis (equal), investigation (lead), methodology (equal), writing – original draft (equal). **Manabu Wen‐Liu Tanimura:** data curation (equal), formal analysis (equal), methodology (equal), writing – review and editing (equal). **Jiaqi Bai:** data curation (equal), formal analysis (equal), investigation (equal), methodology (supporting). **Jie Zheng:** investigation (supporting), methodology (supporting), writing – original draft (supporting). **Liwen Liu:** investigation (equal), methodology (equal). **Pu Yang:** data curation (equal), formal analysis (supporting), writing – review and editing (supporting). **Lizhen Zhang:** project administration (equal), resources (equal), supervision (supporting), writing – review and editing (equal). **Ben Zhang:** funding acquisition (lead), project administration (equal), resources (equal), supervision (lead), writing – review and editing (equal).

## Ethics Statement

The authors have nothing to report.

## Conflicts of Interest

The authors declare no conflicts of interest.

## Supporting information


**Figure S1.** Treatment with 1 g/L PET nanoplastics did not impact the growth of 2‐week‐old “Jingu21” foxtail millet seedlings.
**Figure S2.** Cluster analysis among samples. The horizontal and vertical coordinates are the sample names, and the color represents the distance. The darker the color, the closer the distance is.

**Table S1.** Primer used for qRT‐PCR.
**Table S2.** Transcriptome sequencing quality and reference genome alignment results.
**Table S3.** The differentially expressed genes in foxtail millet after 3 days of PET nanoplastics treatment.
**Table S4.** The differentially expressed genes in foxtail millet after 7 days of PET nanoplastics treatment.
**Table S5.** GO enrichment analysis of differentially expressed genes in foxtail millet after 3 days of PET nanoplastics treatment.
**Table S6.** GO enrichment analysis of differentially expressed genes in foxtail millet after 7 days of PET nanoplastics treatment.
**Table S7.** KEGG enrichment analysis of differentially expressed genes in foxtail millet after 3 days of PET nanoplastics treatment.
**Table S8.** KEGG enrichment analysis of differentially expressed genes in foxtail millet after 7 days of PET nanoplastics treatment.
**Table S9.** KEGG pathway analysis of the unique DEGs in the turquoise module.
**Table S10.** KEGG pathway analysis of the unique DEGs in the red module.
**Table S11.** The standard curve provided by the kit is used by the manufacturer with additional raw data.

## Data Availability

All data generated or analyzed during this study are included in this published article and its Supporting Information files. The raw sequence data reported in this paper have been deposited in the Genome Sequence Archive (Chen et al. 2021) in the National Genomics Data Center (CNCB‐NGDC Members and Partners et al. 2023), China National Center for Bioinformation/Beijing Institute of Genomics, Chinese Academy of Sciences (PRJCA037275; CRA023800 and PRJCA037276; CRA023885) that are publicly accessible at https://ngdc.cncb.ac.cn/gsa/.
