# Supplementary material for: Transcriptome Analysis Reveals the Role of Trehalose in Response to Polyethylene Terephthalate Nanoplastics Treatment in Foxtail Millet ( Setaria italica ) Seedlings
Source: Food Sci Nutr. 2025 Jul 21;13(7):e70593. doi: 10.1002/fsn3.70593 (PMC12280233; doi:10.1002/fsn3.70593)
Supplement: Supplementary file 2 — Table S1. Primer used for qRT‐PCR. Table S2. Transcriptome sequencing quality and reference genome alignment results. Table S3. The differentially expressed genes in foxtail millet after 3 days of PET nanoplastics treatment. Table S4. The differentially expressed genes in foxtail millet after 7 days of PET nanoplastics treatment. Table S5. GO enrichment analysis of differentially expressed genes in foxtail millet after 3 days of PET nanoplastics treatment. Table S6. GO enrichment analysis of differentially expressed genes in foxtail millet after 7 days of PET nanoplastics treatment. Table S7. KEGG enrichment analysis of differentially expressed genes in foxtail millet after 3 days of PET nanoplastics treatment. Table S8. KEGG enrichment analysis of differentially expressed genes in foxtail millet after 7 days of PET nanoplastics treatment. Table S9. KEGG pathway analysis of the unique DEGs in the turquoise module. Table S10. KEGG pathway analysis of the unique DEGs in the red module. Table S11. The standard curve provided by the kit is used by the manufacturer with additional raw data. [file FSN3-13-e70593-s002.docx]

| Table S1. Primer used for qRT-PCR | | | | |
| --- | --- | --- | --- | --- |
| Gene | Gene ID | qRT-PCR Primer F | qRT-PCR Primer R |  |
| *SiAct2* | *Seita.8G043100* | CGCATATGTGGCTCTTGACT | GGGCACCTAAATCTCTCTGC |  |
| *SiRNA POL II* | *Seita.2G142700* | TAGGAAAGGAATTGGCAAGG | TAGGACTGCTTTCGACCCA |  |
| *SiNAC067* | *Seita.2G086600* | GGCAAAGACGGCTCCCTCAG | TCCGCCTCCACGACCTTCTC |  |
| *SiDREB1A* | *Seita.2G280200* | ACATGAGCTGGGACTTGTACTACG | CGTCGCCGAACTCCATGACC |  |
| *SiEDR4* | *Seita.2G434600* | CCCCCTTGCGGAAGAAGATT | CGTAGCCTTCTTGCTTGGGA |  |
| *SiPUB16* | *Seita.5G363900* | ATGAGGTGGGCATCAACGAC | TCCGGCTTGGAATCCGATG |  |
| *SiTPS6* | *Seita.2G197800* | GCTTGTGCAGGGACAAGAAC | GTCCCTCTTAGACCTGAGGA |  |
| *SiTPS7* | *Seita.5G318400* | CACCATCAGGATGCTGACCC | CACCATCAGGATGCTGACCC |  |
| *SiTPS11* | *Seita.1G347300* | AGGTTAACCCCCAGGGCAT | CATTGACGACAGGAGGCTCT |  |
| *SiTPP1* | *Seita.1G262000* | TCATCACCGATCCGTTGTCG | CTCACGTTCATGCTGGGACT |  |
| *SiTPP7* | *Seita.2G188200* | CTGCCCATGATAGAGGAGGTG | ATCTGAAGTGGACGGACACG |  |
| *SiTRE* | *Seita.9G350600* | TACTGGGTCGTCAGGGGATT | CTTGCGCCGTTCAGAACAAA |  |
|  |  |  |  |  |

| Table S2. Transcriptome sequencing quality and reference genome alignment results | | | | | | | | | | | | | | | | | | | |
| --- | --- | --- | --- | --- | --- | --- | --- | --- | --- | --- | --- | --- | --- | --- | --- | --- | --- | --- | --- |
| Sample | RawReads(M) | RawBases(G) | CleanReads(M) | CleanBases(G) | ValidBases(%) | Q30(%) | GC(%) | Total reads | Total mapped reads | Multiple mapped | Uniquely mapped | Read-1 | Read-2 | Reads map to '+' | Reads map to '-' | Non-splice reads | Splice reads | Reads mapped in proper pairs |  |
| TC3d1 | 49.21 | 7.23 | 47.3 | 6.95 | 96.12 | 93.74 | 51.53 | 47298614 | 45390024(95.96%) | 1372961(2.90%) | 44017063(93.06%) | 22022668(46.56%) | 21994395(46.50%) | 21996738(46.51%) | 22020325(46.56%) | 27027132(57.14%) | 16989931(35.92%) | 43656548(92.30%) |  |
| TC3d2 | 50.05 | 7.34 | 48.07 | 7.05 | 96.03 | 94.02 | 51.42 | 48066914 | 45901459(95.49%) | 1488166(3.10%) | 44413293(92.40%) | 22219018(46.23%) | 22194275(46.17%) | 22194795(46.17%) | 22218498(46.22%) | 27514994(57.24%) | 16898299(35.16%) | 44050738(91.64%) |  |
| TC3d3 | 49.46 | 7.26 | 47.14 | 6.92 | 95.31 | 92.8 | 51.23 | 47143630 | 45061534(95.58%) | 1406472(2.98%) | 43655062(92.60%) | 21823608(46.29%) | 21831454(46.31%) | 21812534(46.27%) | 21842528(46.33%) | 26686320(56.61%) | 16968742(35.99%) | 43253668(91.75%) |  |
| TS3d1 | 42.26 | 6.2 | 40.79 | 5.98 | 96.52 | 94.13 | 51.32 | 40792284 | 38927712(95.43%) | 1369507(3.36%) | 37558205(92.07%) | 18776710(46.03%) | 18781495(46.04%) | 18769517(46.01%) | 18788688(46.06%) | 22844487(56.00%) | 14713718(36.07%) | 37257874(91.34%) |  |
| TS3d2 | 45.78 | 6.72 | 44.18 | 6.48 | 96.51 | 94.36 | 51.17 | 44183568 | 42300624(95.74%) | 1314622(2.98%) | 40986002(92.76%) | 20506945(46.41%) | 20479057(46.35%) | 20483218(46.36%) | 20502784(46.40%) | 25074669(56.75%) | 15911333(36.01%) | 40646680(92.00%) |  |
| TS3d3 | 48.43 | 7.12 | 46.82 | 6.88 | 96.68 | 94.57 | 51.07 | 46821494 | 44584434(95.22%) | 1318285(2.82%) | 43266149(92.41%) | 21648572(46.24%) | 21617577(46.17%) | 21623097(46.18%) | 21643052(46.22%) | 26396434(56.38%) | 16869715(36.03%) | 42909050(91.64%) |  |
| TC7d1 | 49.82 | 7.24 | 48.1 | 6.99 | 96.55 | 92.21 | 53.83 | 48100180 | 45458674(94.51%) | 1307478(2.72%) | 44151196(91.79%) | 22762087(47.32%) | 21389109(44.47%) | 22057776(45.86%) | 22093420(45.93%) | 28034576(58.28%) | 16116620(33.51%) | 42437408(88.23%) |  |
| TC7d2 | 50.2 | 7.26 | 48.32 | 6.99 | 96.25 | 92.95 | 54.1 | 48318268 | 46193818(95.60%) | 1274937(2.64%) | 44918881(92.96%) | 22929193(47.45%) | 21989688(45.51%) | 22445860(46.45%) | 22473021(46.51%) | 28572082(59.13%) | 16346799(33.83%) | 43641344(90.32%) |  |
| TC7d3 | 52.36 | 7.39 | 49 | 6.92 | 93.59 | 93.24 | 54.12 | 49001502 | 46676392(95.26%) | 1326200(2.71%) | 45350192(92.55%) | 23219011(47.38%) | 22131181(45.16%) | 22662788(46.25%) | 22687404(46.30%) | 29480995(60.16%) | 15869197(32.39%) | 43915222(89.62%) |  |
| TS7d1 | 49.48 | 7.17 | 47.71 | 6.92 | 96.42 | 92.82 | 53.75 | 47706240 | 45649403(95.69%) | 1379566(2.89%) | 44269837(92.80%) | 22562980(47.30%) | 21706857(45.50%) | 22123155(46.37%) | 22146682(46.42%) | 27767767(58.21%) | 16502070(34.59%) | 43077374(90.30%) |  |
| TS7d2 | 51.35 | 7.37 | 49.03 | 7.04 | 95.47 | 91.64 | 53.45 | 49028906 | 46726967(95.30%) | 1494361(3.05%) | 45232606(92.26%) | 23078431(47.07%) | 22154175(45.19%) | 22600277(46.10%) | 22632329(46.16%) | 28847442(58.84%) | 16385164(33.42%) | 43940528(89.62%) |  |
| TS7d3 | 49.85 | 7.25 | 48.23 | 7.01 | 96.74 | 92.38 | 53.86 | 48225768 | 45987229(95.36%) | 1357885(2.82%) | 44629344(92.54%) | 22810203(47.30%) | 21819141(45.24%) | 22300781(46.24%) | 22328563(46.30%) | 27959882(57.98%) | 16669462(34.57%) | 43303994(89.79%) |  |
|  |  |  |  |  |  |  |  |  |  |  |  |  |  |  |  |  |  |  |  |

| Table S3. The differentially expressed genes in foxtail millet after 3 days of PET nanoplastics treatment | | |
| --- | --- | --- |
| Gene ID | log2FoldChange | Description |
| *Seita.1G001900* | 1.573904929 | Thiosulfate sulfurtransferase 18（STR18） |
| *Seita.1G004600* | -1.136761298 | Beta-fructofuranosidase 1（IVR1） |
| *Seita.1G007800* | 2.887048841 | Dynein 8 kDa light chain, flagellar outer arm |
| *Seita.1G007900* | 1.305205576 | F-box protein At2g26160 |
| *Seita.1G014900* | 1.303263051 | UPF0481 protein At3g47200 |
| *Seita.1G022500* | 1.842113295 | Peroxidase 70（PER70） |
| *Seita.1G025800* | -1.743154839 | Heat stress transcription factor C-2a（HSFC2A） |
| *Seita.1G027600* | -3.583257703 | Unknown |
| *Seita.1G027700* | -1.025104405 | Unknown |
| *Seita.1G029700* | -2.082046175 | Unknown |
| *Seita.1G040100* | -1.35678462 | Unknown |
| *Seita.1G040800* | 1.160373642 | Telomere repeat-binding protein 5（TRP5） |
| *Seita.1G043800* | -1.268575516 | Transcription factor （HY5） |
| *Seita.1G045800* | 1.313200283 | Glucan endo-1,3-beta-glucosidase 14 |
| *Seita.1G054000* | -1.986393411 | Glucomannan 4-beta-mannosyltransferase 1（CSLA1） |
| *Seita.1G054800* | 1.169153404 | Amino acid transporter （AVT6A） |
| *Seita.1G067400* | -1.521444373 | B-box zinc finger protein 32（BBX32） |
| *Seita.1G078200* | -2.562601182 | Unknown |
| *Seita.1G099500* | -2.286324756 | Proteinresponse to low sulfur 1（LSU1） |
| *Seita.1G109000* | 1.338154705 | Unknown |
| *Seita.1G117500* | 2.620917668 | Catalase isozyme 3（CAT3） |
| *Seita.1G133600* | 1.950106092 | Unknown |
| *Seita.1G133700* | 1.467116406 | Unknown |
| *Seita.1G158500* | 1.316175192 | Pentatricopeptide repeat-containing protein At2g01740 |
| *Seita.1G167300* | 1.230909019 | F-box/kelch-repeat protein At1g67480 |
| *Seita.1G167400* | 1.282940061 | Unknown |
| *Seita.1G171900* | -1.16719631 | Glycerophosphodiester phosphodiesterase , chloroplastic（GDPD1） |
| *Seita.1G174900* | 1.143711382 | Unknown |
| *Seita.1G177300* | 1.965655762 | Unknown |
| *Seita.1G181200* | 1.390562085 | Unknown |
| *Seita.1G182300* | -1.474117528 | F-box protein At3g44326 |
| *Seita.1G186200* | -1.79425561 | U-box domain-containing protein 27（PUB27） |
| *Seita.1G186400* | -2.934882431 | E3 ubiquitin-protein ligase （ATL31） |
| *Seita.1G198500* | 1.152055438 | MLO-like protein 1（MLO1） |
| *Seita.1G202200* | 1.176677369 | CASP-like protein 4D1（CASP4D1） |
| *Seita.1G203100* | 1.248350119 | Probable protein phosphatase 2C15（PP2C15） |
| *Seita.1G216200* | 1.128031025 | Inorganic phosphate transporter 2-1, chloroplastic（PHT2-1） |
| *Seita.1G225300* | 1.543888184 | Chitinase 6（Cht6） |
| *Seita.1G227000* | 1.632351947 | Unknown |
| *Seita.1G231800* | -1.069124427 | Adenine phosphoribosyltransferase 2（APT2） |
| *Seita.1G236100* | 3.89803802 | Two-component response regulator-like（PRR1） |
| *Seita.1G239000* | -1.087062667 | Uncharacterized RNA-binding protein C22E12.02 |
| *Seita.1G239600* | -2.023032726 | Phosphoenolpyruvate carboxylase kinase 1（PPCK1） |
| *Seita.1G246000* | 1.261961544 | Receptor kinase-like protein Xa21, processed（XA21） |
| *Seita.1G248300* | -1.697729615 | Ethylene-responsive transcription factor （ERF053） |
| *Seita.1G253700* | -1.701553561 | Homeobox-leucine zipper protein （HOX24） |
| *Seita.1G259000* | -1.581301889 | Ethylene-responsive transcription factor （ERF038） |
| *Seita.1G259200* | -2.229514594 | Ethylene-responsive transcription factor （ERF034） |
| *Seita.1G262000* | -1.05639354 | Probable trehalose-phosphate phosphatase 1（TPP1） |
| *Seita.1G267500* | -1.766807541 | Protein rolling and erect leaf 2（REL2） |
| *Seita.1G268000* | 1.014619869 | Amino acid transporter （AVT3B） |
| *Seita.1G268900* | 1.205584889 | Cellulose synthase-like protein H1（CSLH1） |
| *Seita.1G271000* | 3.340105935 | Dehydration-responsive element-binding protein 1E（DREB1E） |
| *Seita.1G271500* | -1.847711269 | SPX domain-containing membrane protein OsI_08463 |
| *Seita.1G276500* | -2.121042605 | FCS-Like Zinc finger 2（FLZ2） |
| *Seita.1G288100* | 3.959067213 | Abscisic acid 8'-hydroxylase 1（CYP707A5） |
| *Seita.1G288400* | 1.037440969 | 9-cis-epoxycarotenoid dioxygenase, chloroplastic（NCED1） |
| *Seita.1G292400* | 1.477291524 | Unknown |
| *Seita.1G292500* | -1.12428783 | Protein mizu-kussei 1（MIZ1） |
| *Seita.1G294300* | -1.065328795 | Galactan beta-1,4-galactosyltransferase （GALS1） |
| *Seita.1G301300* | 2.557741124 | Zinc finger protein constans-like 9（COL9） |
| *Seita.1G301800* | -1.226678664 | Putative pentatricopeptide repeat-containing protein At3g49142（PCMP-H77） |
| *Seita.1G308800* | 1.769834389 | Zinc finger protein constans-like 16（COL16） |
| *Seita.1G309600* | -3.026147923 | MYB-like transcription factor （ODO1） |
| *Seita.1G310600* | -2.92845754 | Unknown |
| *Seita.1G310800* | -1.276754276 | Nudix hydrolase 18, mitochondrial（NUDT18） |
| *Seita.1G313500* | -2.83867875 | Unknown |
| *Seita.1G315700* | 1.57472595 | Protein jingubang（JGB） |
| *Seita.1G317300* | 1.711234027 | Unknown |
| *Seita.1G317400* | -1.036419565 | Auxin efflux carrier component 1a（PIN1A） |
| *Seita.1G326100* | 3.557072804 | Protein phosphate-induced 1（PHI-1） |
| *Seita.1G326200* | 3.737923072 | Protein phosphate-induced 1 homolog（PHI-1） |
| *Seita.1G326600* | -1.007034193 | 24.1 kDa heat shock protein, mitochondrial（HSP24.1） |
| *Seita.1G327300* | 1.656116438 | E3 ubiquitin-protein ligase（ATL6） |
| *Seita.1G331600* | -1.012815993 | bZIP transcription factor 23（BZIP23） |
| *Seita.1G339600* | -1.131691868 | Fatty-acid-binding protein 2（FAP2） |
| *Seita.1G342400* | -2.066380417 | 18.6 kDa class III heat shock protein（HSP18.6） |
| *Seita.1G345000* | -2.763931875 | Pathogenesis-related protein （PR-1） |
| *Seita.1G347300* | 4.081470203 | Probable alpha,alpha-trehalose-phosphate synthase [UDP-forming] 11（TPS11） |
| *Seita.1G348600* | 1.06204864 | Protein TSS |
| *Seita.1G348800* | 1.331183942 | Unknown |
| *Seita.1G349400* | -1.11440711 | NADPH-dependent aldehyde reductase-like protein, chloroplastic（NADPH） |
| *Seita.1G349700* | -1.596352358 | Protein CDI（CDI） |
| *Seita.1G356800* | 4.86438635 | Auxin-responsive protein （IAA9） |
| *Seita.1G359100* | 1.408024375 | Wall-associated receptor kinase 2（WAK2） |
| *Seita.1G362000* | 2.123374514 | 3-ketoacyl-CoA synthase 3（KCS3） |
| *Seita.1G362500* | -1.11591945 | DeSI-like protein At4g17486 |
| *Seita.1G366500* | 1.806843475 | Unknown |
| *Seita.1G372100* | -1.238247001 | Serine/threonine-protein kinase（PUB33） |
| *Seita.1G376200* | 1.10733096 | Transcription factor （TCP7） |
| *Seita.1G377100* | 1.065533552 | Pentatricopeptide repeat-containing protein At4g36680, mitochondrial |
| *Seita.2G001400* | 1.758474743 | Protein detoxification 18（DTX18） |
| *Seita.2G021200* | 2.810683363 | Unknown |
| *Seita.2G023400* | -2.143196818 | Unknown |
| *Seita.2G025800* | 1.029669597 | L-type lectin-domain containing receptor kinase （SIT2） |
| *Seita.2G026400* | 2.743755772 | Unknown |
| *Seita.2G029800* | 2.929024287 | F-box/FBD/LRR-repeat protein At1g16930 |
| *Seita.2G029900* | 1.290450183 | Unknown |
| *Seita.2G032400* | 1.538972041 | Unknown |
| *Seita.2G035400* | -2.423095735 | 9-cis-epoxycarotenoid dioxygenase, chloroplastic（NCED4） |
| *Seita.2G038100* | 2.011980173 | Unknown |
| *Seita.2G038200* | 1.020935272 | Fasciclin-like arabinogalactan protein 17（FLA17） |
| *Seita.2G050800* | 1.509604724 | Leucine-rich repeat extensin-like protein 6（LRX6） |
| *Seita.2G053300* | -1.140748596 | Low molecular mass early light-inducible protein , chloroplastic（HV90） |
| *Seita.2G056400* | 1.480094143 | Putative disease resistance protein （RGA1） |
| *Seita.2G057000* | 1.137526975 | Putative disease resistance protein （RGA1） |
| *Seita.2G086600* | -1.443507422 | NAC domain-containing protein 67（NAC067） |
| *Seita.2G090600* | -1.437870561 | Zinc transporter 8（ZIP8） |
| *Seita.2G094000* | 2.327678419 | Cytochrome P450 87A3（CYP87A3） |
| *Seita.2G106500* | -1.842377773 | Putative lipid-transfer protein （DIR1） |
| *Seita.2G116100* | -1.339686641 | Probable folate-biopterin transporter 9, chloroplastic（FBT9） |
| *Seita.2G134400* | -2.317682551 | Chlorophyllase-1（CLH1） |
| *Seita.2G140900* | 1.212492744 | Transcription factor （MYB44） |
| *Seita.2G147200* | 1.181879522 | Unknown |
| *Seita.2G155300* | 2.128960413 | Uncharacterized ring finger protein P32A8.03c |
| *Seita.2G177500* | -1.274647359 | Probable protein phosphatase 2C（PP2C68） |
| *Seita.2G197800* | 2.782567369 | Alpha,alpha-trehalose-phosphate synthase [UDP-forming] 6（TPS6） |
| *Seita.2G199400* | 2.229273508 | Probable inactive receptor kinase （RLK902） |
| *Seita.2G199600* | -1.322125661 | Unknown |
| *Seita.2G200700* | -1.232903078 | Putative 1-phosphatidylinositol-3-phosphate 5-kinase （FAB1C） |
| *Seita.2G202300* | -1.592788957 | Unknown |
| *Seita.2G203100* | -1.168639344 | Unknown |
| *Seita.2G206200* | -1.219706192 | CBL-interacting protein kinase 16（CIPK16） |
| *Seita.2G213800* | 1.843173245 | AAA-ATPase At4g25835 |
| *Seita.2G216500* | 2.423191598 | Dormancy-associated protein homolog 3 |
| *Seita.2G217500* | -1.460928803 | Protein TIFY 10c |
| *Seita.2G220400* | -1.942752892 | Serine/threonine-protein kinase （RIPK） |
| *Seita.2G221900* | -3.204394943 | Unknown |
| *Seita.2G222100* | -1.102852088 | Protein Brevis radix-like 1（BRXL1） |
| *Seita.2G222800* | -1.70421497 | Unknown |
| *Seita.2G222900* | -1.832073558 | Zinc finger protein 8（ZFP8） |
| *Seita.2G227900* | -1.481262408 | F-box protein At1g10780 |
| *Seita.2G228700* | -1.853911659 | Transcription factor （HEC1） |
| *Seita.2G229000* | 1.085725142 | Tuliposide A-converting enzyme b1, amyloplastic（TCEA-B1） |
| *Seita.2G229900* | 1.618597896 | Abscisic acid 8'-hydroxylase 3（CYP707A7） |
| *Seita.2G232900* | -2.069676814 | Tuliposide A-converting enzyme 1, chloroplastic（TCEA1） |
| *Seita.2G236200* | -2.199688871 | GLABRA2 expression modulator（GEM） |
| *Seita.2G238400* | -1.321787027 | E3 ubiquitin-protein ligase （ATL6） |
| *Seita.2G242400* | -1.85237807 | Dof zinc finger protein （MNB1A） |
| *Seita.2G251700* | 3.238763107 | Tonoplast dicarboxylate transporter（TDT） |
| *Seita.2G259000* | -1.241240279 | Unknown |
| *Seita.2G261500* | 2.988540799 | Unknown |
| *Seita.2G263000* | 1.927406286 | Transcription factor （BHLH49） |
| *Seita.2G275500* | 1.839986327 | UDP-glucosyltransferase UGT13248 |
| *Seita.2G280000* | 3.985867514 | Dehydration-responsive element-binding protein 1A（DREB1A） |
| *Seita.2G280100* | 4.290961177 | Dehydration-responsive element-binding protein 1A（DREB1A） |
| *Seita.2G280200* | 5.037795493 | Dehydration-responsive element-binding protein 1A（DREB1A） |
| *Seita.2G280300* | 4.276980628 | Dehydration-responsive element-binding protein 1A（DREB1A） |
| *Seita.2G280400* | 5.135853263 | Dehydration-responsive element-binding protein 1A（DREB1A） |
| *Seita.2G282600* | 2.389950085 | UDP-glucose 4-epimerase 3（UGE-3） |
| *Seita.2G289600* | -1.195683587 | Ribonuclease 1（RNS1） |
| *Seita.2G290300* | -1.908422482 | Unknown |
| *Seita.2G291500* | 2.450145687 | Probable aquaporin （PIP2-7） |
| *Seita.2G293600* | -3.741528046 | Putrescine hydroxycinnamoyltransferase 3（PHT3） |
| *Seita.2G295000* | 2.677437165 | Auxin-responsive protein （SAUR39） |
| *Seita.2G302500* | 1.252421554 | Protein pin-likes 7（PILS7） |
| *Seita.2G313800* | -2.198727124 | Probable xyloglucan endotransglucosylase/hydrolase protein 32（XTH32） |
| *Seita.2G325500* | -1.374553065 | Filament-like plant protein 3（FPP3） |
| *Seita.2G327500* | -1.006746064 | Probable 5'-adenylylsulfate reductase 1, chloroplastic（APR1） |
| *Seita.2G331700* | -1.017293514 | Unknown |
| *Seita.2G332800* | 2.346270121 | Unknown |
| *Seita.2G335500* | -1.287382005 | Septum-promoting GTP-binding protein 1（SPG1） |
| *Seita.2G351900* | 1.482709496 | Unknown |
| *Seita.2G352400* | -1.532265971 | Dynein 8 kDa light chain, flagellar outer arm |
| *Seita.2G360300* | -1.010824404 | Unknown |
| *Seita.2G362000* | -1.649033801 | Unknown |
| *Seita.2G365400* | -1.495378713 | L-type lectin-domain containing receptor kinase （SIT2） |
| *Seita.2G381600* | -1.025570879 | Ninja-family protein 5 |
| *Seita.2G401300* | -3.412592575 | E3 ubiquitin-protein ligase （MPSR1） |
| *Seita.2G404300* | -1.047792116 | Cytochrome P450 （CYP709B2） |
| *Seita.2G410400* | -3.499825159 | Probable carboxylesterase 18（CXE18） |
| *Seita.2G415300* | -1.881497577 | Beta-glucosidase 26（BGLU26） |
| *Seita.2G416600* | 1.582909544 | Xylan glycosyltransferase （MUCI21） |
| *Seita.2G420200* | 2.851178617 | Unknown |
| *Seita.2G428900* | 1.67789285 | Universal stress protein A-like protein |
| *Seita.2G432000* | -1.276202102 | CBL-interacting protein kinase 2（CIPK2） |
| *Seita.2G434600* | 1.598498633 | Protein Enhanced disease resistance 4（EDR4） |
| *Seita.2G436600* | 2.026350868 | Thioredoxin-like 1-1, chloroplastic |
| *Seita.2G440700* | 1.108880464 | Unknown |
| *Seita.3G007200* | 1.902143816 | E3 ubiquitin-protein ligase（PUB23） |
| *Seita.3G007800* | -1.434619925 | Unknown |
| *Seita.3G009900* | 1.971650582 | AT-hook motif nuclear-localized protein 5（AHL5） |
| *Seita.3G014100* | 3.811722846 | Stem-specific protein （TSJT1） |
| *Seita.3G020400* | 1.897775128 | Polyamine oxidase 5（PAO5） |
| *Seita.3G020500* | 2.847602842 | Unknown |
| *Seita.3G020600* | 1.029948741 | Polyamine oxidase 4（PAO4） |
| *Seita.3G023900* | -1.559687014 | Unknown |
| *Seita.3G028500* | 1.296963224 | Beta-fructofuranosidase, insoluble isoenzyme 7（CIN7） |
| *Seita.3G031600* | -1.51957112 | Ethylene-responsive transcription factor 3（ERF3） |
| *Seita.3G037000* | 1.445788712 | Heavy metal-associated isoprenylated plant protein 36（HIPP36） |
| *Seita.3G037900* | 1.265107423 | 1-aminocyclopropane-1-carboxylate oxidase 5（ACO5） |
| *Seita.3G044600* | 1.924718627 | Apetala2-like protein 1（AP2-1） |
| *Seita.3G044700* | 1.243537256 | Unknown |
| *Seita.3G046400* | 1.011738689 | Exonuclease V, chloroplastic |
| *Seita.3G047600* | 1.347146807 | L-type lectin-domain containing receptor kinase VIII.1（LECRK81） |
| *Seita.3G048500* | 1.464916187 | Phosphoinositide phospholipase C2（PLC2） |
| *Seita.3G053800* | 1.22126195 | CBL-interacting protein kinase 17（CIPK17） |
| *Seita.3G055100* | -4.909138487 | Unknown |
| *Seita.3G055700* | -1.475128643 | Transcription factor Phytochrome interacting factor-like 15（PIL15） |
| *Seita.3G060600* | 1.218455577 | G-type lectin S-receptor-like serine/threonine-protein kinase At2g19130 |
| *Seita.3G062400* | 1.058975875 | Myb-related protein （MYBAS2） |
| *Seita.3G068600* | 1.10844484 | WAT1-related protein At5g64700 |
| *Seita.3G075300* | 1.453208989 | GDSL esterase/lipase At5g45910 |
| *Seita.3G076200* | 1.762138002 | Abscisic acid receptor （PYL5） |
| *Seita.3G076300* | 1.469729317 | Putative disease resistance protein （RGA1） |
| *Seita.3G078700* | -1.165126836 | Transcription repressor （OFP7） |
| *Seita.3G083300* | 1.350915127 | Putative receptor protein kinase（PK1） |
| *Seita.3G088000* | 1.476888189 | E3 ubiquitin-protein ligase （WAV3） |
| *Seita.3G092300* | -2.457023977 | Noroxomaritidine synthase 2（Cyp96T2） |
| *Seita.3G093400* | 1.57097443 | Protein NRT1/ PTR family 4.3（NPF4.3） |
| *Seita.3G097900* | 3.29801376 | B-box zinc finger protein 22（BBX22） |
| *Seita.3G103200* | 1.609415053 | Unknown |
| *Seita.3G104400* | 2.058197896 | Unknown |
| *Seita.3G105100* | 1.770100806 | Leucine-rich repeat extensin-like protein 5（LRX5） |
| *Seita.3G106800* | -1.654299057 | Unknown |
| *Seita.3G109900* | 1.243099267 | Unknown |
| *Seita.3G110500* | -3.117030949 | Stem 28 kDa glycoprotein（VSPA） |
| *Seita.3G120400* | 2.18847628 | Unknown |
| *Seita.3G123800* | -1.555126594 | Aspartyl protease family protein 2（APF2） |
| *Seita.3G124200* | 2.913911211 | Transcription factor （KUA1） |
| *Seita.3G124300* | 2.192032994 | RNA polymerase sigma factor （SIGC） |
| *Seita.3G132400* | -1.482432312 | Unknown |
| *Seita.3G136500* | -1.660439227 | Unknown |
| *Seita.3G138200* | 1.361503564 | Unknown |
| *Seita.3G138700* | -1.322188416 | CTP synthase（URA7） |
| *Seita.3G139000* | -1.234149246 | Probable protein phosphatase 2C8（PP2C8） |
| *Seita.3G144600* | -1.266569179 | Uncharacterized protein ycf23 |
| *Seita.3G145200* | -1.285487948 | Mitogen-activated protein kinase 7（MPK7） |
| *Seita.3G147500* | -1.559703076 | Protein psi1（PSI1） |
| *Seita.3G155000* | -2.696391931 | Unknown |
| *Seita.3G155800* | 1.57731025 | Unknown |
| *Seita.3G156600* | -1.206430973 | Unknown |
| *Seita.3G158300* | 1.528774789 | AP2/ERF and B3 domain-containing protein Os01g0693400 |
| *Seita.3G160800* | -1.986794299 | Mitogen-activated protein kinase kinase kinase 17（MAPKKK17） |
| *Seita.3G164400* | 1.747982694 | Probable ADP,ATP carrier protein At5g56450 |
| *Seita.3G168400* | -2.037910812 | Unknown |
| *Seita.3G173400* | 1.542137933 | Unknown |
| *Seita.3G176300* | 2.359096285 | Unknown |
| *Seita.3G176500* | -1.744754995 | VQ motif-containing protein 4（VQ4） |
| *Seita.3G176700* | -1.767590842 | Unknown |
| *Seita.3G183000* | 2.204893843 | Probable aspartyl protease At4g16563 |
| *Seita.3G186600* | -1.042661423 | Protein sulfur deficency-induced 1（SDI1） |
| *Seita.3G190000* | -1.59059782 | UDP-glycosyltransferase 73E1（UGT73E1） |
| *Seita.3G191900* | -1.212108034 | Ethylene-responsive transcription factor 4（ERF4） |
| *Seita.3G193800* | -3.077364717 | EID1-like F-box protein 3（EDL3） |
| *Seita.3G196100* | 2.822832575 | Unknown |
| *Seita.3G196200* | 2.922463119 | SNF1-related protein kinase regulatory subunit beta-1（KINB1） |
| *Seita.3G196600* | 1.511761913 | Probable calcium-binding protein （CML9） |
| *Seita.3G205500* | -1.981942581 | U-box domain-containing protein 16（PUB16） |
| *Seita.3G212900* | 1.364231406 | Unknown |
| *Seita.3G216400* | 1.251258276 | GTP cyclohydrolase-2（RIBA3） |
| *Seita.3G216900* | -1.751949981 | Heat shock 70 kDa protein 4（HSP70-4） |
| *Seita.3G218800* | -1.194310532 | Probable protein phosphatase 2C 49 |
| *Seita.3G225200* | 1.079654165 | Glucan endo-1,3-beta-glucosidase 14 |
| *Seita.3G225500* | 1.599545553 | Transcription factor （IVARICATA） |
| *Seita.3G227200* | 3.928630071 | RING-H2 finger protein （ATL79） |
| *Seita.3G236700* | 1.334165251 | Probable receptor-like protein kinase At1g11050 |
| *Seita.3G242400* | 1.029762395 | Transcription factor （MYBC1） |
| *Seita.3G246600* | -1.642867322 | Unknown |
| *Seita.3G251400* | 1.154319713 | Cyclin-P3-1（CYCP3-1） |
| *Seita.3G251900* | 1.57930966 | Unknown |
| *Seita.3G255900* | 2.154100351 | Unknown |
| *Seita.3G261400* | -1.953541278 | Probable calcium-binding protein （CML15） |
| *Seita.3G263800* | 6.275869863 | Lichenase-2 |
| *Seita.3G281800* | 3.317273796 | Protein early responsive to dehydration 15（ERD15） |
| *Seita.3G284500* | -1.019534438 | CBL-interacting protein kinase 22（CIPK22） |
| *Seita.3G297700* | 5.945262077 | Isoflavone reductase homolog |
| *Seita.3G298100* | -1.585396161 | Unknown |
| *Seita.3G300700* | 2.422385217 | Chloride channel protein （CLC-A） |
| *Seita.3G311700* | -1.207711401 | Subtilisin-chymotrypsin inhibitor-2A |
| *Seita.3G325100* | -1.336675948 | Unknown |
| *Seita.3G325400* | 1.023668269 | Scarecrow-like protein 9（SCL9） |
| *Seita.3G330700* | 3.347810721 | Aspartic proteinase nepenthesin-1（NEP1） |
| *Seita.3G338000* | -1.196821707 | Major pollen allergen Aln g 1 |
| *Seita.3G339800* | -1.005018002 | Cytochrome P450 76M5（CYP76M5） |
| *Seita.3G340200* | 2.188681918 | Patatin-like protein 1（PLP1） |
| *Seita.3G344000* | 1.557875839 | Glutaredoxin-C15（GRXC15） |
| *Seita.3G353500* | -1.629605385 | Unknown |
| *Seita.3G367100* | 1.73473893 | Unknown |
| *Seita.3G368200* | 1.021614194 | Zinc finger protein 7（ZFP7） |
| *Seita.3G368300* | 2.652429409 | Cytochrome P450（CYP81Q32） |
| *Seita.3G370000* | 1.543111324 | Myb family transcription factor （EFM） |
| *Seita.3G373500* | -2.417917465 | Protein jingubang （JGB） |
| *Seita.3G379500* | -1.309346656 | Auxin-responsive protein （IAA31） |
| *Seita.3G379700* | 2.579105687 | Light-inducible protein （CPRF2） |
| *Seita.3G380200* | -1.054024476 | CBL-interacting protein kinase 4（CIPK4） |
| *Seita.3G381000* | 1.194071381 | Unknown |
| *Seita.3G382100* | 1.766082382 | Methylcrotonoyl-CoA carboxylase subunit alpha, mitochondrial（MCCA） |
| *Seita.3G386800* | -1.087544659 | Patatin-like protein 3（PLP3） |
| *Seita.4G003100* | 1.309400798 | Monocopper oxidase-like protein （SKU5） |
| *Seita.4G005100* | 1.677723669 | Unknown |
| *Seita.4G015600* | -1.233912336 | Gibberellin 2-beta-dioxygenase 8（GA2OX8） |
| *Seita.4G020200* | -1.549428669 | Unknown |
| *Seita.4G021100* | -4.885979599 | Unknown |
| *Seita.4G025500* | -2.357250329 | Uncharacterized protein At4g22758 |
| *Seita.4G031400* | -1.199841435 | Palmitoyl-acyl carrier protein thioesterase, chloroplastic（FATB） |
| *Seita.4G038600* | 1.404216917 | Unknown |
| *Seita.4G040400* | 2.189949847 | Aspartic proteinase nepenthesin-2（NEP2） |
| *Seita.4G041800* | -1.131626419 | Unknown |
| *Seita.4G042500* | -1.148953333 | RING-H2 finger protein （ATL60） |
| *Seita.4G046600* | 1.958038167 | Putative leucine-rich repeat receptor-like serine/threonine-protein kinase At2g24130 |
| *Seita.4G067200* | -1.526127661 | Probable WRKY transcription factor 53（WRKY53） |
| *Seita.4G070400* | 2.129782619 | F-box/LRR-repeat MAX2 homolog（D3） |
| *Seita.4G075200* | -1.049895355 | Cysteine synthase, chloroplastic/chromoplastic（CYSK） |
| *Seita.4G091400* | -1.250705379 | Unknown |
| *Seita.4G097900* | 1.035954625 | Transcription factor （TCP14） |
| *Seita.4G100500* | -1.837212823 | BAG family molecular chaperone regulator 5, mitochondrial（BAG5） |
| *Seita.4G105600* | 3.123185009 | Protein jingubang（JGB） |
| *Seita.4G112600* | 3.263734407 | Unknown |
| *Seita.4G116600* | 3.84148486 | Zinc finger protein constans-like 16（COL16） |
| *Seita.4G125900* | 1.423602544 | Unknown |
| *Seita.4G134400* | -2.204136283 | Beta-glucosidase 30（BGLU30） |
| *Seita.4G138500* | -1.173065624 | Sodium/hydrogen exchanger 4（NHX4） |
| *Seita.4G168200* | 3.030268724 | Pentatricopeptide repeat-containing protein At3g57430, chloroplastic（PCMP-H81） |
| *Seita.4G173400* | 2.126878644 | Unknown |
| *Seita.4G176600* | 1.137885582 | Peroxidase 52（PER52） |
| *Seita.4G178800* | 2.246409063 | Unknown |
| *Seita.4G181300* | -1.073858105 | Unknown |
| *Seita.4G183200* | 1.743032811 | Unknown |
| *Seita.4G187300* | 1.530601182 | bZIP transcription factor （RISBZ5） |
| *Seita.4G188900* | -3.603534334 | B-box zinc finger protein 32（BBX32） |
| *Seita.4G190600* | -2.419242591 | Unknown |
| *Seita.4G193400* | -2.678897745 | Unknown |
| *Seita.4G194900* | -1.237896636 | Hydrophobic protein （OSR8） |
| *Seita.4G197200* | 1.644360904 | Protein polar localization during asymmetric division and redistribution（POLAR） |
| *Seita.4G199100* | 1.25125559 | Unknown |
| *Seita.4G199400* | 1.683075966 | Unknown |
| *Seita.4G203000* | -2.664972234 | Zealexin A1 synthase（CYP71Z18） |
| *Seita.4G214500* | 2.419966728 | VQ motif-containing protein 25（VQ25） |
| *Seita.4G221600* | -1.445108218 | Unknown |
| *Seita.4G222700* | -1.225047533 | SPX domain-containing protein 1（SPX1） |
| *Seita.4G225400* | 1.680553407 | 3-ketoacyl-CoA synthase 11（KCS11） |
| *Seita.4G226700* | 3.792343554 | F-box/kelch-repeat protein At1g80440 |
| *Seita.4G227700* | -1.799494297 | Multiprotein-bridging factor 1c（MBF1C） |
| *Seita.4G232700* | 1.320292142 | Putative receptor protein kinase Zm（PK1） |
| *Seita.4G244600* | 2.056130671 | Unknown |
| *Seita.4G246200* | 3.266957154 | Probable xyloglucan endotransglucosylase/hydrolase protein 23（XTH23） |
| *Seita.4G250000* | -1.815965148 | Probable receptor-like serine/threonine-protein kinase At5g57670 |
| *Seita.4G262000* | -1.00385415 | Unknown |
| *Seita.4G263400* | -1.538213208 | NAC domain-containing protein 21/22（NAC021） |
| *Seita.4G266000* | -1.848332558 | Auxin-responsive protein （SAUR71） |
| *Seita.4G274800* | 2.460975904 | Unknown |
| *Seita.4G274900* | 1.19020202 | WAT1-related protein At3g45870 |
| *Seita.4G281200* | 2.866432463 | Chaperone protein dnaJ 11, chloroplastic（ATJ11） |
| *Seita.5G010800* | -1.706930874 | Transcription repressor （OFP7） |
| *Seita.5G017100* | 2.992324666 | Unknown |
| *Seita.5G017200* | 2.842505085 | V-type proton ATPase subunit E（VATE） |
| *Seita.5G025200* | -1.541399697 | Myb-related protein |
| *Seita.5G025600* | -1.180594228 | Unknown |
| *Seita.5G027400* | -1.259126001 | Acyl-CoA-binding domain-containing protein 4（ACBP4） |
| *Seita.5G033400* | 1.119445597 | Hydrophobic protein （LTI6B） |
| *Seita.5G033500* | 1.36041166 | Hydrophobic protein （LTI6B） |
| *Seita.5G034500* | -1.338475566 | Transcription factor （MYB61） |
| *Seita.5G034800* | -1.088781896 | Unknown |
| *Seita.5G049200* | 3.202956566 | Unknown |
| *Seita.5G058800* | 1.462248035 | Phospholipase A1 PLIP2, chloroplastic（PLIP2） |
| *Seita.5G065300* | 1.719324814 | Chaperone protein dnaJ 20, chloroplastic（ATJ20） |
| *Seita.5G067800* | 2.320632793 | Light-regulated protein, chloroplastic（LIR1） |
| *Seita.5G091400* | -1.805913767 | Probable leucine-rich repeat receptor-like protein kinase At1g68400 |
| *Seita.5G092600* | -1.386974403 | 17.9 kDa heat shock protein 2（HSP17.9B） |
| *Seita.5G095900* | -2.78191508 | Reticulon-like protein B8（RTNLB8） |
| *Seita.5G096900* | 1.598660218 | AP2/ERF and B3 domain-containing protein Os01g0141000 |
| *Seita.5G099500* | 1.216524525 | Thylakoid lumenal 15 kDa protein 1, chloroplastic |
| *Seita.5G109600* | -1.130259467 | Probable carboxylesterase 15（CXE15） |
| *Seita.5G120800* | 2.046464209 | Pentatricopeptide repeat-containing protein At1g66345, mitochondrial |
| *Seita.5G121800* | 1.356336073 | Unknown |
| *Seita.5G126800* | 2.501372915 | Acyl transferase 7（AT7） |
| *Seita.5G132900* | 1.531344487 | Protein twin lov 1（TLP1） |
| *Seita.5G137500* | -1.4370006 | Monothiol glutaredoxin-S2（GRXS2） |
| *Seita.5G140700* | -1.47464042 | Unknown |
| *Seita.5G140800* | -5.086738348 | Unknown |
| *Seita.5G147400* | -2.26995758 | Gibberellin 2-beta-dioxygenase 3（GA2OX3） |
| *Seita.5G157600* | 2.969239715 | Neutral/alkaline invertase 1, mitochondrial（NIN1） |
| *Seita.5G167100* | 2.828470789 | Glutaredoxin-C1（GRXC1） |
| *Seita.5G169500* | -2.071663417 | 12-oxophytodienoate reductase 1（OPR1） |
| *Seita.5G203200* | 1.010664136 | Cysteine-rich receptor-like protein kinase 6（CRK6） |
| *Seita.5G203400* | 2.179586642 | Glucan endo-1,3-beta-glucosidase 13 |
| *Seita.5G204500* | -3.038466962 | Transcription factor （BHLH87） |
| *Seita.5G214300* | -1.130137241 | Aspartyl protease family protein 2（APF2） |
| *Seita.5G233100* | 1.856678293 | WRKY transcription factor 22（WRKY22） |
| *Seita.5G235900* | 2.926705522 | Unknown |
| *Seita.5G242600* | 2.389287325 | Unknown |
| *Seita.5G260400* | -2.400747735 | Mannan endo-1,4-beta-mannosidase 1（MAN1） |
| *Seita.5G274800* | -1.274702854 | Unknown |
| *Seita.5G279400* | 1.577526956 | Unknown |
| *Seita.5G279500* | 2.187992214 | Unknown |
| *Seita.5G280700* | 1.393908766 | AP2/ERF and B3 domain-containing protein Os01g0693400 |
| *Seita.5G281200* | 1.264233684 | Probable LRR receptor-like serine/threonine-protein kinase At3g47570 |
| *Seita.5G281400* | 1.30670738 | Receptor kinase-like protein, processed（XA21） |
| *Seita.5G284300* | -2.114044922 | Mitogen-activated protein kinase kinase kinase 17（MAPKKK17） |
| *Seita.5G287300* | 1.960454494 | F-box/kelch-repeat protein At1g23390 |
| *Seita.5G303500* | 1.663698687 | Probable galacturonosyltransferase 15（GAUT15） |
| *Seita.5G305300* | 2.094211988 | Unknown |
| *Seita.5G305800* | 1.007028088 | Chaperone protein dnaJ 10（ATJ10） |
| *Seita.5G306000* | 1.92493394 | Probable WRKY transcription factor 69（WRKY69） |
| *Seita.5G309000* | 1.653860299 | Anthocyanidin 5,3-O-glucosyltransferase（RhGT1） |
| *Seita.5G311600* | 1.199596297 | Unknown |
| *Seita.5G312700* | 1.06429076 | Auxin-responsive protein （IAA6） |
| *Seita.5G315500* | -1.566706066 | Chaperone protein （ClpB1） |
| *Seita.5G316100* | 1.192482014 | Unknown |
| *Seita.5G318400* | 2.426585566 | Probable alpha,alpha-trehalose-phosphate synthase [UDP-forming] 7（TPS7） |
| *Seita.5G319300* | 1.829190371 | Protein JLP2 |
| *Seita.5G327000* | 1.579960051 | Triacylglycerol lipase （SDP1） |
| *Seita.5G336800* | -4.549906521 | Transcription factor （BHLH148） |
| *Seita.5G348000* | -1.266602606 | Ethylene-responsive transcription factor 4（ERF4） |
| *Seita.5G348100* | -1.318353585 | Ethylene-responsive transcription factor 12（ERF12） |
| *Seita.5G351800* | -1.568971329 | WAT1-related protein At5g07050 |
| *Seita.5G363900* | 1.910252629 | U-box domain-containing protein 16（PUB16） |
| *Seita.5G369900* | 1.218252881 | Unknown |
| *Seita.5G371800* | -1.499130161 | Unknown |
| *Seita.5G376100* | -1.312078911 | Heat shock 70 kDa protein（HSP70） |
| *Seita.5G379400* | -1.245565991 | Probable protein phosphatase 2C9（PP2C9） |
| *Seita.5G382800* | 2.667178006 | Laccase-7（LAC7） |
| *Seita.5G386600* | -1.311791704 | Unknown |
| *Seita.5G391200* | 1.365309354 | NAC domain-containing protein 90（NAC090） |
| *Seita.5G394300* | 2.165523447 | Ethylene-responsive transcription factor （ERF110） |
| *Seita.5G408100* | 1.908902857 | Unknown |
| *Seita.5G415100* | 1.141574835 | Unknown |
| *Seita.5G432300* | 1.133686466 | Putative disease resistance RPP13-like protein 1（RPPL1） |
| *Seita.5G432600* | 2.373175031 | Unknown |
| *Seita.5G442200* | -1.260109399 | Protein SRC2 |
| *Seita.5G443100* | 2.031584958 | Esterase PIR7B |
| *Seita.5G443200* | 3.231557232 | Probable esterase PIR7A |
| *Seita.5G444000* | -1.345043357 | Protein SRG1 |
| *Seita.5G460400* | 1.738040896 | Unknown |
| *Seita.5G468000* | 1.732085421 | Unknown |
| *Seita.6G009500* | 1.46137194 | Serine/threonine-protein kinase-like protein （CCR4） |
| *Seita.6G014300* | -3.48498566 | Endo-1,3 |
| *Seita.6G023100* | -5.374419431 | Cytochrome P450 99A2（CYP99A2） |
| *Seita.6G023700* | -1.395570522 | Ethylene-responsive transcription factor 7（ERF7） |
| *Seita.6G023800* | -1.970425523 | Ethylene-responsive transcription factor 7（ERF7） |
| *Seita.6G032700* | 1.376109221 | NAC domain-containing protein 21/22（NAC021） |
| *Seita.6G045900* | 1.081758595 | Nuclear transcription factor Y subunit B-11（HD5） |
| *Seita.6G068400* | -3.981919992 | (E)-beta-caryophyllene synthase（TPS23） |
| *Seita.6G070700* | 1.419212253 | Uclacyanin 1（UCC1） |
| *Seita.6G075700* | 1.414228054 | Protein LOL4 |
| *Seita.6G081700* | 1.260350749 | Ma3 domain-containing translation regulatory factor（MRF1） |
| *Seita.6G089200* | 2.911871999 | Unknown |
| *Seita.6G098100* | -2.24050507 | Unknown |
| *Seita.6G110100* | -2.096995523 | Probable monogalactosyldiacylglycerol synthase 2, chloroplastic（MGD2） |
| *Seita.6G122300* | 1.082998818 | Unknown |
| *Seita.6G122900* | 1.073345622 | Bifunctional nuclease 2（BBD2） |
| *Seita.6G136200* | 2.79962155 | BTB/POZ and MATH domain-containing protein 1（BPM1） |
| *Seita.6G136800* | 1.829892409 | Hypersensitive-induced response protein 1（HIR1） |
| *Seita.6G138300* | -1.703357412 | Unknown |
| *Seita.6G139100* | -1.086353866 | Secoisolariciresinol dehydrogenase |
| *Seita.6G144600* | 1.549054689 | Probable alpha,alpha-trehalose-phosphate synthase [UDP-forming] 9（TPS9） |
| *Seita.6G159400* | -1.200332506 | Ribonuclease 1（RNS1） |
| *Seita.6G161100* | 2.262886283 | Zinc-finger homeodomain protein 9（ZHD9） |
| *Seita.6G163200* | -2.517357451 | CBL-interacting protein kinase 6（CIPK6） |
| *Seita.6G165800* | -1.10341342 | Tetraspanin-8（TET8） |
| *Seita.6G170000* | 3.43446068 | Auxin-responsive protein （SAUR76） |
| *Seita.6G170200* | 2.629744415 | Auxin-responsive protein （SAUR76） |
| *Seita.6G170400* | 1.795209508 | Dormancy-associated protein homolog 3 |
| *Seita.6G173200* | 1.075815788 | Unknown |
| *Seita.6G174400* | -1.470164375 | Cytokinin dehydrogenase 11（CKX11） |
| *Seita.6G179700* | 1.542359845 | Unknown |
| *Seita.6G182600* | -1.090659083 | Tuliposide A-converting enzyme 1, chloroplastic（TCEA1） |
| *Seita.6G182700* | -1.000219323 | Tuliposide A-converting enzyme 2, chloroplastic（TCEA2） |
| *Seita.6G184400* | 1.190808685 | DnaJ protein （ERDJ7） |
| *Seita.6G185100* | 1.601532229 | AT-hook motif nuclear-localized protein 17（AHL17） |
| *Seita.6G186400* | 1.134012108 | E3 ubiquitin-protein ligase （ATL6） |
| *Seita.6G189200* | -1.796966142 | Alpha carbonic anhydrase 7（ACA7） |
| *Seita.6G190600* | 1.021424791 | Unknown |
| *Seita.6G193400* | 1.314423803 | Oligopeptide transporter 5（OPT5） |
| *Seita.6G195300* | 1.057762365 | Probable galactinol--sucrose galactosyltransferase 1（RFS1） |
| *Seita.6G198600* | 1.060237235 | Probable WRKY transcription factor 2（WRKY2） |
| *Seita.6G200300* | -1.230785879 | Heat shock protein 81-1（HSP81-1） |
| *Seita.6G210000* | 1.179518762 | Putative L-ascorbate peroxidase 6（APX6） |
| *Seita.6G211600* | -3.336434017 | Unknown |
| *Seita.6G221700* | -3.544850481 | F-box protein At5g07610 |
| *Seita.6G226000* | 1.092818218 | Unknown |
| *Seita.6G226200* | 1.857950771 | Zinc finger protein comstans-like 15（COL15） |
| *Seita.6G234900* | 1.200621095 | Molybdenum cofactor sulfurase（MCSU3） |
| *Seita.6G235000* | 1.112858856 | Sorbitol dehydrogenase（SDH） |
| *Seita.6G236100* | -1.455728241 | Heat stress transcription factor B-2b（HSFB2B） |
| *Seita.6G239700* | 1.521752763 | Myb-related protein Hv1（MYB1） |
| *Seita.7G014100* | 1.522509179 | Protein SRC2 |
| *Seita.7G015100* | 6.248163719 | Unknown |
| *Seita.7G032900* | -1.832922815 | Probable inorganic phosphate transporter 1-4（PHT1-4） |
| *Seita.7G035400* | 3.619232914 | Unknown |
| *Seita.7G076400* | 1.11427427 | ACT domain-containing protein （ACR6） |
| *Seita.7G077700* | 1.427989486 | Unknown |
| *Seita.7G084900* | -1.247129689 | Unknown |
| *Seita.7G090200* | -1.38235798 | Protein BIC1 |
| *Seita.7G095100* | 1.321642099 | U-box domain-containing protein 27（PUB27） |
| *Seita.7G105000* | 1.239325621 | Glycolipid transfer protein 3（GLTP3） |
| *Seita.7G112100* | 3.8975202 | Scarecrow-like protein 9（SCL9） |
| *Seita.7G131700* | -1.002582177 | Disease resistance protein （PIK-1） |
| *Seita.7G135400* | -1.141457211 | Putative protein Brevis radix-like 5（BRXL5） |
| *Seita.7G140200* | -2.577781235 | Protein jingubang （JGB） |
| *Seita.7G149900* | 2.34920105 | Transcription factor （BHLH153） |
| *Seita.7G169600* | -1.185665886 | Auxin response factor 10（ARF10） |
| *Seita.7G179400* | 1.931445231 | (+)-neomenthol dehydrogenase（MNR1） |
| *Seita.7G179600* | -1.597880499 | Transcription factor （LAF1） |
| *Seita.7G188700* | -1.769310259 | Ethylene-responsive transcription factor （ERF038） |
| *Seita.7G202400* | 1.286663107 | Unknown |
| *Seita.7G202900* | -2.020895444 | Heat stress transcription factor B-2a（HSFB2A） |
| *Seita.7G205200* | 4.671220175 | Dehydration-responsive element-binding protein 1E（DREB1E） |
| *Seita.7G209000* | -1.168401441 | Beta-carotene hydroxylase 2, chloroplastic（BCH2） |
| *Seita.7G212300* | 1.970628447 | Unknown |
| *Seita.7G212900* | 1.289280639 | Protein reveille 1（RVE1） |
| *Seita.7G214500* | -1.048294469 | FCS-Like Zinc finger 1（FLZ1） |
| *Seita.7G214600* | -1.306431305 | FCS-Like Zinc finger 1（FLZ1） |
| *Seita.7G214900* | -1.041545903 | FCS-Like Zinc finger 1（FLZ1） |
| *Seita.7G217100* | 2.428355221 | Unknown |
| *Seita.7G228100* | -1.115058571 | Plant intracellular Ras-group-related LRR protein 1（IRL1） |
| *Seita.7G230900* | -1.744998764 | Auxin-responsive protein （SAUR71） |
| *Seita.7G232100* | -1.910854819 | Ethylene-responsive transcription factor 4（ERF4） |
| *Seita.7G241600* | 1.424824646 | Putative disease resistance protein （RGA4） |
| *Seita.7G251900* | 1.196730402 | Receptor-like serine/threonine-protein kinase （SD18） |
| *Seita.7G254100* | 2.871024897 | Unknown |
| *Seita.7G262800* | 1.179523269 | Ethylene-responsive transcription factor （ERF008） |
| *Seita.7G267900* | 1.053527249 | GATA transcription factor 23（GATA23） |
| *Seita.7G272000* | 1.239581193 | Receptor-like protein kinase （HERK 1） |
| *Seita.7G274400* | 3.685905187 | Unknown |
| *Seita.7G274700* | 1.517118909 | Glucuronoxylan 4-O-methyltransferase 3（GXM3） |
| *Seita.7G278800* | 2.099717101 | Zealexin A1 synthase（CYP71Z18） |
| *Seita.7G285800* | 1.257531295 | Unknown |
| *Seita.7G288200* | 1.232882104 | Transcription factor （TCP8） |
| *Seita.7G292900* | -1.111165641 | Unknown |
| *Seita.7G296600* | 1.1034625 | Protein smax1-like 3（SMXL3） |
| *Seita.7G300300* | 1.325500212 | CBL-interacting protein kinase 14（CIPK14） |
| *Seita.7G303400* | 1.446327776 | Unknown |
| *Seita.7G319100* | 2.917436047 | Cyanidin 3-O-rutinoside 5-O-glucosyltransferase（C3G5GT） |
| *Seita.7G323700* | 1.987493765 | Cytochrome P450 （CYP94C1） |
| *Seita.7G328000* | 1.049870525 | Unknown |
| *Seita.8G008100* | 1.027969104 | NAC transcription factor 56（NAC056） |
| *Seita.8G015900* | 1.743051961 | Unknown |
| *Seita.8G016000* | 2.378036935 | BTB/POZ domain-containing protein At3g56230 |
| *Seita.8G022900* | 4.418648222 | Putative receptor-like protein kinase At3g47110 |
| *Seita.8G025300* | 2.930740299 | Unknown |
| *Seita.8G025400* | 1.043773934 | Probable calcium-transporting ATPase 9, plasma membrane-type（ACA9） |
| *Seita.8G027000* | 1.250988644 | Cytochrome P450 90A4（CYP90A4） |
| *Seita.8G028400* | -1.356001729 | Heavy metal-associated isoprenylated plant protein 32（HIPP32） |
| *Seita.8G032600* | -1.681944424 | Unknown |
| *Seita.8G037500* | 1.732271928 | Unknown |
| *Seita.8G037700* | 2.084274431 | Protein smax1-like 3（SMXL3） |
| *Seita.8G049400* | 1.920293847 | Filament-like plant protein 3（FPP3） |
| *Seita.8G050900* | -1.632833114 | Unknown |
| *Seita.8G055100* | 2.047096455 | Unknown |
| *Seita.8G056100* | 1.259781645 | Dirigent protein 21（DIR21） |
| *Seita.8G058100* | -1.548046729 | Unknown |
| *Seita.8G074800* | 1.666435226 | Shewanella-like protein phosphatase 2（SLP2） |
| *Seita.8G122400* | -5.298051591 | Probable methyltransferase At1g27930 |
| *Seita.8G123100* | 1.082612431 | Probable WRKY transcription factor 75（WRKY75） |
| *Seita.8G128400* | -1.979782524 | BAG family molecular chaperone regulator 5, mitochondrial（BAG5） |
| *Seita.8G141100* | -1.175981208 | Unknown |
| *Seita.8G152100* | 1.967368815 | Wall-associated receptor kinase 1（WAK1） |
| *Seita.8G152800* | 1.867345644 | Protein LYK5 |
| *Seita.8G179400* | 1.823257089 | Unknown |
| *Seita.8G212900* | -6.204990576 | Laccase-19（LAC19） |
| *Seita.8G215100* | -1.753263667 | Dirigent protein 1（DIR1） |
| *Seita.8G215500* | 1.588209238 | Kinesin-like protein KIN-14O（KIN14O） |
| *Seita.8G218900* | -3.284524807 | Unknown |
| *Seita.8G219700* | 2.488017839 | Glutaredoxin-C10（GRXC10） |
| *Seita.8G242600* | 1.270222733 | Putative disease resistance protein （RGA1） |
| *Seita.8G242800* | 1.381702597 | Putative disease resistance protein （RGA1） |
| *Seita.8G243800* | 1.371602118 | Putative disease resistance protein （RGA1） |
| *Seita.8G244000* | 1.277486234 | Putative disease resistance protein （RGA1） |
| *Seita.8G247600* | 1.727894308 | Tuliposide A-converting enzyme 2, chloroplastic（TCEA2） |
| *Seita.9G004600* | 2.193725817 | Ribosome-binding factor, chloroplastic（PSRP1） |
| *Seita.9G005800* | 1.230623447 | Probable xyloglucan endotransglucosylase/hydrolase protein 30（XTH30） |
| *Seita.9G018800* | 1.970193068 | Unknown |
| *Seita.9G019600* | -2.026160292 | G-type lectin S-receptor-like serine/threonine-protein kinase At1g34300 |
| *Seita.9G030600* | 2.889895969 | Bowman-Birk type trypsin inhibitor |
| *Seita.9G031500* | -1.909466526 | Unknown |
| *Seita.9G039200* | -1.360005236 | NAC domain-containing protein 2（NAC002） |
| *Seita.9G042400* | -1.047011274 | (+)-neomenthol dehydrogenase（SDR1） |
| *Seita.9G050000* | -1.237026555 | AAA-ATPase ASD, mitochondrial（AATP1） |
| *Seita.9G051600* | 1.463846482 | Thioredoxin H2-2 |
| *Seita.9G064500* | 1.217508266 | Probable glutathione S-transferase（GSTU1） |
| *Seita.9G067200* | 1.141548207 | Transcription factor phytochrome interacting factor-like 13（PIL13） |
| *Seita.9G074100* | 3.87860977 | Unknown |
| *Seita.9G074500* | 1.003653982 | L-type lectin-domain containing receptor kinase （SIT2） |
| *Seita.9G079200* | 4.145497371 | CCG-binding protein 1（MEE14） |
| *Seita.9G081900* | -1.600579118 | Subtilisin-like protease （SBT1.7） |
| *Seita.9G083400* | 1.879678541 | Cytochrome P450 81Q32（CYP81Q32） |
| *Seita.9G083600* | 1.011080075 | Cytochrome P450 81Q32（CYP81Q32） |
| *Seita.9G086500* | 2.296935371 | UDP-glycosyltransferase 83A1（UGT83A1） |
| *Seita.9G112100* | -2.72210676 | Unknown |
| *Seita.9G112800* | -1.038112676 | Probable histone H2AXa |
| *Seita.9G115800* | 1.473789771 | Receptor protein kinase （TMK1） |
| *Seita.9G125800* | -1.857373224 | Unknown |
| *Seita.9G137200* | -1.086509824 | Gamma-glutamyl peptidase 5（GGP5） |
| *Seita.9G156500* | -1.928870598 | 9-cis-epoxycarotenoid dioxygenase 1, chloroplastic（VP14） |
| *Seita.9G165700* | 1.739672268 | Unknown |
| *Seita.9G170900* | 1.823579832 | Zinc finger protein 7（ZFP7） |
| *Seita.9G171600* | -3.926345814 | Unknown |
| *Seita.9G179600* | -1.528367015 | Unknown |
| *Seita.9G180100* | 1.811025252 | Unknown |
| *Seita.9G188100* | 4.284146852 | Dormancy-associated protein 1（DRM1） |
| *Seita.9G190200* | 1.035652678 | AAA-ATPase At2g46620 |
| *Seita.9G196800* | 3.004928127 | 21 kDa protein |
| *Seita.9G198200* | 2.737604405 | Putative disease resistance protein （RGA3） |
| *Seita.9G216200* | 1.397029905 | Uncharacterized aminotransferase C6B12.04c |
| *Seita.9G217200* | 1.243724806 | Cytokinin dehydrogenase 3（CKX3） |
| *Seita.9G227900* | 2.101063798 | Unknown |
| *Seita.9G228000* | 1.521467941 | Unknown |
| *Seita.9G229700* | -1.571293028 | Beta-amylase 1, chloroplastic（BAMY1） |
| *Seita.9G234200* | 4.878788169 | E3 ubiquitin-protein ligase （RZFP34） |
| *Seita.9G238100* | 1.920005661 | E3 ubiquitin-protein ligase （AIRP2） |
| *Seita.9G239700* | -1.597902859 | UDP-glycosyltransferase 86A1（UGT86A1） |
| *Seita.9G253000* | -2.935536956 | SPX domain-containing protein 3（SPX3） |
| *Seita.9G254900* | 1.272595338 | Probable S-adenosylmethionine carrier 2, chloroplastic（SAMC2） |
| *Seita.9G257300* | -2.908022175 | Unknown |
| *Seita.9G259600* | 1.471275182 | Unknown |
| *Seita.9G271400* | 1.177720878 | F-box/kelch-repeat protein At1g22040 |
| *Seita.9G277500* | 1.991323578 | WAT1-related protein At4g08290 |
| *Seita.9G298200* | 2.730828639 | Peroxidase 53（PER53） |
| *Seita.9G301000* | 1.071208354 | Homeobox-leucine zipper protein （HOX15） |
| *Seita.9G301200* | 2.760713525 | Unknown |
| *Seita.9G306400* | -1.563010053 | Choline-phosphate cytidylyltransferase 2（CCT2） |
| *Seita.9G311100* | 1.125929958 | Protein rgf1 inducible transcription factor 1（RITF1） |
| *Seita.9G311400* | -1.391657372 | RING-H2 finger protein （ATL28） |
| *Seita.9G325200* | -3.611382245 | GATA transcription factor 4（GATA4） |
| *Seita.9G326100* | -2.476382288 | Expansin-B3（EXPB3） |
| *Seita.9G327100* | -2.770837016 | Expansin-B6（EXPB6） |
| *Seita.9G334800* | -1.194838966 | Unknown |
| *Seita.9G341900* | -1.529669303 | Heavy metal-associated isoprenylated plant protein 30（HIPP30） |
| *Seita.9G344400* | 1.033016119 | Uncharacterized protein PAM68-like |
| *Seita.9G349000* | 2.491728842 | F-box protein PP2-A13（PP2A13） |
| *Seita.9G349200* | 1.359503671 | Unknown |
| *Seita.9G355300* | 1.27467567 | Protein short-root 1（SHR1） |
| *Seita.9G365500* | 1.065901559 | Glucose-6-phosphate 1-dehydrogenase, chloroplastic（G6PDH） |
| *Seita.9G367000* | 1.467724945 | Unknown |
| *Seita.9G368900* | 1.804304837 | Unknown |
| *Seita.9G369800* | -1.968987263 | SPX domain-containing protein 5（SPX5） |
| *Seita.9G373200* | 2.071473732 | Ring-H2 finger protein （ATL3） |
| *Seita.9G394900* | 1.207313347 | Probable serine/threonine-protein kinase （PBL15） |
| *Seita.9G400900* | 1.670957907 | Protein iq-domain 14（IQD14） |
| *Seita.9G407000* | -2.489335499 | Desiccation-related protein PCC13-62 |
| *Seita.9G409400* | 1.012719258 | Dormancy-associated protein homolog 3 |
| *Seita.9G417000* | -1.562617078 | Uncharacterized protein At5g39570 |
| *Seita.9G422600* | 1.14071621 | Unknown |
| *Seita.9G427100* | -1.873244245 | Calcium-binding protein （CP1） |
| *Seita.9G432200* | -1.837570126 | Myb-related protein P |
| *Seita.9G436000* | -1.165002346 | Sex determination protein tasselseed-2（TS2） |
| *Seita.9G436600* | 1.538598891 | Sucrose transport protein （SUT2） |
| *Seita.9G437300* | 1.214829323 | Abscisic acid receptor （PYL4） |
| *Seita.9G440100* | -1.042952319 | DnaJ protein （ERDJ3A） |
| *Seita.9G440300* | -1.070428775 | Mitogen-activated protein kinase kinase kinase 17（MAPKKK17） |
| *Seita.9G440500* | -1.416169375 | Probable protein phosphatase 2C 32（PP2C32） |
| *Seita.9G451900* | -1.822508514 | Heat shock 70 kDa protein 4（HSP70-4） |
| *Seita.9G453400* | 1.199588542 | Telomere repeat-binding protein 5（TRP5） |
| *Seita.9G456100* | 1.17520148 | Uncharacterized TPR repeat-containing protein At1g05150 |
| *Seita.9G461500* | 1.783236419 | Hsp70 nucleotide exchange factor（FES1） |
| *Seita.9G461800* | -1.120159305 | Aspartic proteinase （PCS1） |
| *Seita.9G465200* | 2.072167908 | Unknown |
| *Seita.9G465600* | 1.757970235 | Protein far1-related sequence 7（FRS7） |
| *Seita.9G468200* | -1.771201358 | Unknown |
| *Seita.9G469300* | -1.803199844 | Cytochrome P450（CYP76C2） |
| *Seita.9G472200* | -1.49185904 | Endo-1,4-beta-xylanase 5-like |
| *Seita.9G474600* | 1.116050968 | Zinc finger protein 4（ZFP4） |
| *Seita.9G478600* | 1.204827896 | Metal tolerance protein 4（MTP4） |
| *Seita.9G480700* | -1.237674876 | Leucine-rich repeat receptor-like serine/threonine-protein kinase（BAM1） |
| *Seita.9G487000* | 1.778466585 | G-type lectin S-receptor-like serine/threonine-protein kinase At2g19130 |
| *Seita.9G497900* | 1.476581561 | BTB/POZ domain-containing protein（SR1IP1） |
| *Seita.9G500200* | 1.314286019 | Strigolactone esterase （D14） |
| *Seita.9G504800* | -1.525126388 | Polyol transporter 5（PLT5） |
| *Seita.9G505000* | -1.148103925 | Polyol transporter 5（PLT5） |
| *Seita.9G509400* | -1.397068228 | Ethylene-responsive transcription factor （ERF0570） |
| *Seita.9G511700* | -1.695193659 | Transcription factor （BHLH96） |
| *Seita.9G512800* | -1.323251069 | Transport inhibitor response 1-like protein（TIR1） |
| *Seita.9G520900* | 1.738244148 | Probable S-adenosylmethionine carrier 2, chloroplastic（SAMC2） |
| *Seita.9G523100* | 1.626945078 | Unknown |
| *Seita.9G525000* | 1.071487616 | Cyclic dof factor 1（CDF1） |
| *Seita.9G531100* | 1.217249371 | PDDEXK-like family (PDDEXK6) |
| *Seita.9G533300* | 1.413076181 | Unknown |
| *Seita.9G536500* | 1.386420665 | Monooxygenase 2（MO2） |
| *Seita.9G542400* | -1.299287444 | Xyloglucan galactosyltransferase katamari1 homolog（XyGT-Kat1） |
| *Seita.9G545200* | -1.256975955 | tetratricopeptide repeat protein, TPR |
| *Seita.9G550800* | 1.605667822 | Unknown |
| *Seita.9G552100* | -1.703711693 | Probable signal recognition particle 43 kDa protein, chloroplastic |
| *Seita.9G555000* | 1.385850466 | MLO protein homolog 1（MLO-H1） |
| *Seita.9G561500* | 1.095256995 | Flavanone 3-dioxygenase 2（F3H-2） |
| *Seita.9G562600* | -1.26269482 | Peroxidase 5（SBTM4） |
| *Seita.9G564500* | 2.835286136 | Subtilisin-like protease 4 |
| *Seita.9G567300* | 1.098796888 | F-box protein At4g00755 |
| *Seita.9G567400* | 3.447315137 | F-box protein（PP2A13） |
| *Seita.J003100* | 2.523617934 | Peroxidase 1（PRX74） |
|  |  |  |

| Table S4. The differentially expressed genes in foxtail millet after 7 days of PET nanoplastics treatment | | |
| --- | --- | --- |
| Gene ID | log_2_FoldChange | Description |
| *Seita.1G014700* | -1.324120419 | Unknown |
| *Seita.1G015300* | -1.614979279 | Unknown |
| *Seita.1G015400* | -1.473453454 | Unknown |
| *Seita.1G025100* | 1.150911734 | Aquaporin NIP1-1 |
| *Seita.1G036400* | -2.038958116 | Unknown |
| *Seita.1G053500* | -1.981090466 | Unknown |
| *Seita.1G056000* | -1.573547742 | Cleavage site for pathogenic type III effector avirulence factor Avr（CS-PT3E-Avr） |
| *Seita.1G062100* | -1.095734725 | WRKY transcription factor （WRKY71） |
| *Seita.1G095600* | -2.106040069 | Unknown |
| *Seita.1G165400* | -5.33594867 | Unknown |
| *Seita.1G170200* | -1.246562602 | Peroxidase 2（PER2） |
| *Seita.1G186200* | -1.601244813 | U-box domain-containing protein 27（PUB27） |
| *Seita.1G186400* | -2.974033621 | E3 ubiquitin-protein ligase ATL31（ATL31） |
| *Seita.1G197500* | -1.018593912 | E3 ubiquitin-protein ligase （EL5） |
| *Seita.1G201400* | -1.282409159 | Unknown |
| *Seita.1G206000* | -1.068235289 | Unknown |
| *Seita.1G208100* | -1.00073479 | 7-deoxyloganetin glucosyltransferase（UGT85A24） |
| *Seita.1G217800* | -1.480449668 | Suppressor of gamma response 1（SOG1） |
| *Seita.1G239400* | -1.2102407 | Transcription factor （MYB30） |
| *Seita.1G270500* | -1.331855633 | Protein detoxification 49（DTX49） |
| *Seita.1G270800* | -1.812591643 | Ethylene-responsive transcription factor （ERF025） |
| *Seita.1G271000* | -3.189537757 | Dehydration-responsive element-binding protein 1E（DREB1E） |
| *Seita.1G273100* | -1.387711799 | Brassinosteroid-responsive ring protein 1（BRH1） |
| *Seita.1G274900* | -1.172965787 | Unknown |
| *Seita.1G290700* | 1.010619275 | Dof zinc finger protein （DOF5.6） |
| *Seita.1G295900* | -1.07851354 | Unknown |
| *Seita.1G312600* | -1.106818301 | Unknown |
| *Seita.1G314500* | -1.365935657 | Formin-like protein 16（FH16） |
| *Seita.1G314700* | -1.017284142 | Probable galacturonosyltransferase-like 9（GATL9） |
| *Seita.1G315500* | -1.074375848 | Unknown |
| *Seita.1G317300* | -1.276633909 | Unknown |
| *Seita.1G326100* | -1.99411479 | Protein phosphate-induced 1（PHI-1） |
| *Seita.1G326200* | -4.011770544 | Protein phosphate-induced 1 homolog（PHI-1） |
| *Seita.1G326900* | -1.75197166 | Unknown |
| *Seita.1G327300* | -1.320068492 | E3 ubiquitin-protein ligase （ATL6） |
| *Seita.1G336700* | 1.836133123 | Protein trichome birefringence（TBR） |
| *Seita.1G341100* | -1.468530647 | Ethylene-responsive transcription factor （ERF016） |
| *Seita.2G023400* | -2.004849884 | CAMP-response element binding protein-related（CREB） |
| *Seita.2G026400* | -1.495478276 | Unknown |
| *Seita.2G038100* | -1.05163336 | Unknown |
| *Seita.2G038200* | -1.60981341 | Fasciclin-like arabinogalactan protein 17（FLA17） |
| *Seita.2G069800* | -1.019026329 | AAA-ATPase At3g28580 |
| *Seita.2G086600* | -1.027576569 | NAC domain-containing protein 67（NAC067） |
| *Seita.2G095600* | 1.976678641 | Unknown |
| *Seita.2G140900* | -1.184348697 | Transcription factor （MYB44） |
| *Seita.2G179800* | -1.186123392 | Transcription factor （WRKY19） |
| *Seita.2G188200* | -1.38353655 | Probable trehalose-phosphate phosphatase 7（TPP7） |
| *Seita.2G195200* | -1.212267888 | Zinc finger an1 domain-containing stress-associated protein 17（SAP17） |
| *Seita.2G199400* | -1.544223693 | Probable inactive receptor kinase （RLK902） |
| *Seita.2G199500* | -1.241130733 | Probable inactive receptor kinase （RLK902） |
| *Seita.2G202300* | -1.419903195 | Unknown |
| *Seita.2G216900* | -1.097103021 | Unknown |
| *Seita.2G225500* | 1.047523855 | 1-aminocyclopropane-1-carboxylate oxidase 1（ACO1） |
| *Seita.2G242700* | -1.066342755 | Fasciclin-like arabinogalactan protein 1（FLA1） |
| *Seita.2G249000* | 1.191692395 | Aspartyl protease family protein （At5g10770） |
| *Seita.2G249200* | 1.375603244 | Aspartyl protease family protein （At5g10770） |
| *Seita.2G252000* | -1.168967579 | Unknown |
| *Seita.2G257400* | -1.299649657 | Unknown |
| *Seita.2G276500* | 1.095725979 | UDP-glucosyltransferase （UGT13248） |
| *Seita.2G280000* | -2.008340344 | Dehydration-responsive element-binding protein 1A（DREB1A） |
| *Seita.2G280100* | -2.420412641 | Dehydration-responsive element-binding protein 1A（DREB1A） |
| *Seita.2G280200* | -1.945189239 | Dehydration-responsive element-binding protein 1A（DREB1A） |
| *Seita.2G280400* | -1.103232399 | Dehydration-responsive element-binding protein 1A（DREB1A） |
| *Seita.2G347200* | -1.164670766 | G-type lectin S-receptor-like serine/threonine-protein kinase （B120） |
| *Seita.2G352400* | -1.052470162 | Dynein 8 kDa light chain, flagellar outer arm |
| *Seita.2G355700* | -1.226023055 | Unknown |
| *Seita.2G363900* | -1.554799846 | Unknown |
| *Seita.2G375000* | -1.218311451 | Momilactone A synthase |
| *Seita.2G393700* | 2.327410565 | F-box/FBD/LRR-repeat protein （At1g80470） |
| *Seita.2G416600* | -1.088879694 | Xylan glycosyltransferase （MUCI21） |
| *Seita.2G418500* | 1.017272006 | Phosphatidylinositol 4-phosphate 5-kinase 4（PIP5K4） |
| *Seita.2G434600* | -1.882468477 | Protein enhanced disease resistance 4（EDR4） |
| *Seita.3G004800* | 2.860205964 | Cationic peroxidase （SPC4） |
| *Seita.3G008300* | -1.442510265 | NDR1/HIN1-like protein 3（NHL3） |
| *Seita.3G024600* | 1.172265742 | Probable amino acid permease 7（AAP7） |
| *Seita.3G037900* | -1.414996877 | 1-aminocyclopropane-1-carboxylate oxidase 5（ACO5） |
| *Seita.3G047600* | -1.033635522 | L-type lectin-domain containing receptor kinase VIII.1（LECRK81） |
| *Seita.3G048400* | -1.063543779 | Uncharacterized protein At4g08330, chloroplastic（At4g08330） |
| *Seita.3G057300* | 1.196192281 | Unknown |
| *Seita.3G066800* | -1.747232901 | E3 ubiquitin-protein ligase （RDUF1） |
| *Seita.3G077900* | -3.013239205 | Arabinogalactan protein 16（AGP16） |
| *Seita.3G089400* | 1.173325078 | Thioredoxin H2-2 |
| *Seita.3G103300* | -1.079681923 | Unknown |
| *Seita.3G105100* | -1.315868459 | Leucine-rich repeat extensin-like protein 5（LRX5） |
| *Seita.3G108500* | -1.341645894 | Probable WRKY transcription factor 50（WRKY50） |
| *Seita.3G110000* | -1.169556271 | Leucine-rich repeat extensin-like protein 3（LRX3） |
| *Seita.3G126800* | -1.036761227 | Unknown |
| *Seita.3G135500* | -1.148120979 | Probable calcium-binding protein （CML14） |
| *Seita.3G137000* | 1.219394528 | Unknown |
| *Seita.3G137100* | 1.651362694 | Unknown |
| *Seita.3G138700* | 1.132190251 | CTP synthase（URA7） |
| *Seita.3G148100* | 5.052914811 | Gibberellin 2-beta-dioxygenase 3（GA2OX3） |
| *Seita.3G163300* | -1.67285662 | Protein IQ-DOMAIN 1（IQD1） |
| *Seita.3G164900* | -1.052805994 | Probable WRKY transcription factor 51（WRKY51） |
| *Seita.3G206900* | -1.320912328 | WRKY transcription factor （WRKY24） |
| *Seita.3G207000* | -2.077554088 | Unknown |
| *Seita.3G225400* | -1.72108659 | NAC domain-containing protein 90（NAC090） |
| *Seita.3G247800* | -1.057897591 | Unknown |
| *Seita.3G261400* | -1.268052953 | Probable calcium-binding protein （CML15） |
| *Seita.3G286100* | -2.051885611 | Unknown |
| *Seita.3G304600* | 1.18740829 | Unknown |
| *Seita.3G353500* | -1.741374089 | Unknown |
| *Seita.3G367100* | -1.013324954 | Unknown |
| *Seita.3G380800* | -1.3337272 | Calmodulin-like protein 5（CML5） |
| *Seita.3G406500* | 1.307592255 | Protein nrt1/ptr family 2.11（NPF2.11） |
| *Seita.4G004900* | 1.186969415 | Probable polygalacturonase |
| *Seita.4G008800* | -1.059553712 | Cellulose synthase-like protein D2（CSLD2） |
| *Seita.4G016400* | -2.447594429 | Dehydration-responsive element-binding protein 1C（DREB1C） |
| *Seita.4G018100* | -1.927674242 | Unknown |
| *Seita.4G022700* | -1.530728233 | Unknown |
| *Seita.4G022900* | -1.047555354 | Protein exordium - like 3（EXL3） |
| *Seita.4G079000* | -1.002486954 | Unknown |
| *Seita.4G093200* | 3.919829705 | Agamous-like mads-box protein （AGL104） |
| *Seita.4G108400* | -2.004693127 | Probable galacturonosyltransferase-like 9（GATL9） |
| *Seita.4G148800* | -1.31391964 | Protein radialis - like 5（RL5） |
| *Seita.4G154100* | -2.106433532 | Receptor-like protein （EIX2） |
| *Seita.4G169300* | -1.405117753 | Unknown |
| *Seita.4G178900* | -1.274842153 | Unknown |
| *Seita.4G194900* | -1.23844364 | Hydrophobic protein OSR8 |
| *Seita.4G200200* | 1.430166269 | Unknown |
| *Seita.4G225400* | -1.96336422 | 3-ketoacyl-CoA synthase 11（KCS11） |
| *Seita.4G227700* | -1.051238511 | Multiprotein-bridging factor 1c（MBF1C） |
| *Seita.4G246400* | -1.897138881 | Xyloglucan endotransglucosylase/hydrolase protein 22（XTH22） |
| *Seita.4G255900* | -1.494143376 | Calcium-binding protein （PBP1） |
| *Seita.4G284800* | 1.016284713 | Expansin-like A4（EXLA4） |
| *Seita.5G009900* | 1.161703438 | Cytochrome P450 71A6（CYP71A6） |
| *Seita.5G022000* | -1.059933874 | Ethylene-responsive transcription factor （ERF071） |
| *Seita.5G067100* | -1.449382605 | Unknown |
| *Seita.5G132800* | -2.167842492 | Probable wrky transcription factor 50（WRKY50） |
| *Seita.5G144000* | -1.290484175 | Unknown |
| *Seita.5G149600* | -1.069382401 | Signaling peptide taximin 1（TAX1） |
| *Seita.5G158400* | 1.240252917 | Unknown |
| *Seita.5G176000* | -1.533231771 | Unknown |
| *Seita.5G178200* | -1.314858642 | Xylan glycosyltransferase （MUCI21） |
| *Seita.5G235900* | -1.694467109 | Unknown |
| *Seita.5G236500* | -5.303068447 | Unknown |
| *Seita.5G236700* | -1.802453143 | Unknown |
| *Seita.5G251100* | -1.133469779 | Putative glucose-6-phosphate 1-epimerase |
| *Seita.5G261800* | -1.52418492 | Unknown |
| *Seita.5G269700* | 1.04636476 | Unknown |
| *Seita.5G294500* | -1.760119193 | Probable wrky transcription factor 51（WRKY51） |
| *Seita.5G299000* | -1.230613451 | Protein sodium potassium root defective 2（NAKR2） |
| *Seita.5G312000* | -1.360062814 | Unknown |
| *Seita.5G326900* | -1.03079481 | Protein nrt1/ptr family 1.2（NPF1.2） |
| *Seita.5G331000* | -1.020594057 | Probable xyloglucan glycosyltransferase 1（CSLC1） |
| *Seita.5G337400* | 1.172127105 | Disease resistance protein （RPM1） |
| *Seita.5G346700* | 2.035816655 | Subtilisin-like protease （SBT3.5） |
| *Seita.5G363900* | -1.000895583 | U-box domain-containing protein 16（PUB16） |
| *Seita.5G371600* | -1.251698107 | Unknown |
| *Seita.5G371700* | -1.450689736 | Unknown |
| *Seita.5G374200* | -1.443418747 | Unknown |
| *Seita.5G377300* | -1.168613112 | Elicitor-responsive protein 1（ERG1） |
| *Seita.5G378700* | -1.020634346 | Aspartic proteinase （PCS1） |
| *Seita.5G379200* | -1.00457373 | Unknown |
| *Seita.5G381400* | -1.117782988 | Ras-related protein （RAB11C） |
| *Seita.5G391200* | -1.11292501 | NAC domain-containing protein 90（NAC090） |
| *Seita.5G422900* | 1.281534094 | Unknown |
| *Seita.5G448400* | 1.148801247 | Lichenase-2 |
| *Seita.5G451500* | -1.554666435 | Probable calcium-binding protein （CML10） |
| *Seita.5G455600* | -2.552570494 | Unknown |
| *Seita.5G456800* | -1.589003362 | Probable calcium-binding protein （CML31） |
| *Seita.6G003900* | 3.70494034 | Indole-2-monooxygenase（CYP71C4） |
| *Seita.6G004000* | 1.868275836 | Indole-2-monooxygenase（CYP71C4） |
| *Seita.6G004100* | 1.634130979 | Indole-2-monooxygenase（CYP71C4） |
| *Seita.6G017500* | 1.448825039 | Disease resistance protein （RGA2） |
| *Seita.6G023100* | 3.437512385 | Cytochrome P450 99A2（CYP99A2） |
| *Seita.6G023700* | -1.644204674 | Ethylene-responsive transcription factor 7（ERF7） |
| *Seita.6G023800* | -1.579017753 | Ethylene-responsive transcription factor 7（ERF7） |
| *Seita.6G048500* | 1.21235855 | (S)-beta-macrocarpene synthase（TPS11） |
| *Seita.6G052500* | -1.112097802 | Probable mixed-linked glucan synthase 6（CSLF6） |
| *Seita.6G093100* | 1.025472343 | Proline--tRNA ligase, cytoplasmic |
| *Seita.6G128200* | 1.043011667 | Rab escort protein 1（REP） |
| *Seita.6G132800* | -1.1242226 | Transcription factor （WRKY19） |
| *Seita.6G144400* | -1.477777823 | Unknown |
| *Seita.6G159400* | 1.213386234 | Ribonuclease 1 |
| *Seita.6G165800* | -1.967357757 | Tetraspanin-8 |
| *Seita.6G170000* | -3.019327216 | Auxin-responsive protein （SAUR76） |
| *Seita.6G170200* | -1.78631506 | Auxin-responsive protein （SAUR76） |
| *Seita.6G170300* | 1.050440722 | DnaJ homolog subfamily C member 7 homolog |
| *Seita.6G205000* | -1.674754978 | IRK-interacting protein（IRKI） |
| *Seita.7G090400* | -2.527202737 | Glucan endo-1,3-beta-glucosidase 14（Glu14） |
| *Seita.7G094400* | -1.029840105 | Protein MKS1 |
| *Seita.7G099400* | 1.296055858 | Subtilisin-like protease （SBT1.5） |
| *Seita.7G112000* | -1.883735584 | Scarecrow-like protein 33（SCL33） |
| *Seita.7G124900* | 1.018735215 | NAC domain-containing protein 79（NAC079） |
| *Seita.7G128200* | -1.155027573 | Peroxidase 65 |
| *Seita.7G164900* | -1.11935352 | Disease resistance protein （RPS2） |
| *Seita.7G177500* | -1.532623134 | Sphingolipid delta(4)-desaturase DES1-like |
| *Seita.7G204900* | -1.667587744 | Protein detoxification 49（DTX49） |
| *Seita.7G205200* | -3.10605615 | Dehydration-responsive element-binding protein 1E（DREB1E） |
| *Seita.7G206600* | 1.943010148 | Cytochrome p450 704c1（CYP704C1） |
| *Seita.7G216000* | 1.035685285 | Probable purine permease 11（PUP11） |
| *Seita.7G227400* | -2.472556571 | Xyloglucan endotransglucosylase/hydrolase protein 24（XTH24） |
| *Seita.7G239900* | 1.066207618 | LRR receptor-like serine/threonine-protein kinase （FLS2） |
| *Seita.7G248700* | 1.127304253 | Kinesin-like protein （KIN-14H） |
| *Seita.7G253600* | -1.134842617 | Unknown |
| *Seita.7G260400* | -1.419091684 | 36.4 kDa proline-rich protein（TPRP-F1） |
| *Seita.7G296300* | -1.161612812 | NDR1/HIN1-like protein 2（NHL2） |
| *Seita.8G008500* | -1.093203355 | Probable galacturonosyltransferase 7（GAUT7） |
| *Seita.8G033400* | 1.718810045 | Purple acid phosphatase 22（PAP22） |
| *Seita.8G042800* | 1.060872975 | Putative disease resistance protein （RGA3） |
| *Seita.8G057400* | -5.330782166 | Unknown |
| *Seita.8G073800* | 1.566026353 | Cysteine-rich receptor-like protein kinase 25（CRK25） |
| *Seita.8G099200* | -1.510174448 | Unknown |
| *Seita.8G099600* | -2.127537062 | Unknown |
| *Seita.8G123100* | -1.0104144 | Probable WRKY transcription factor 75（WRKY75） |
| *Seita.8G143300* | -1.146033184 | E3 ubiquitin-protein ligase （BOI） |
| *Seita.8G153700* | -1.42535725 | UDP-glycosyltransferase 72B1（UGT72B1） |
| *Seita.8G203700* | 1.204882787 | Unknown |
| *Seita.9G018800* | -1.907660591 | Unknown |
| *Seita.9G069700* | 1.580292256 | Transcription factor jungbrunnen 1（JUB1） |
| *Seita.9G074100* | -2.575113576 | Unknown |
| *Seita.9G080500* | -1.722318902 | Zinc finger protein 1（ZFP1） |
| *Seita.9G082200* | -1.016417858 | Probable protein phosphatase 2C 34（PP2C34） |
| *Seita.9G086100* | -2.293156746 | UDP-glycosyltransferase 83A1（UGT83A1） |
| *Seita.9G086800* | -1.550680413 | UDP-glycosyltransferase 83A1（UGT83A1） |
| *Seita.9G099200* | -3.482314094 | Unknown |
| *Seita.9G103400* | -2.929142924 | Unknown |
| *Seita.9G103500* | -2.672961316 | Unknown |
| *Seita.9G115800* | -1.014525712 | Receptor protein kinase （TMK1） |
| *Seita.9G142900* | 1.726164456 | Bisdemethoxycurcumin synthase |
| *Seita.9G149300* | 1.925649381 | LOB domain-containing protein 4（LBD4） |
| *Seita.9G153500* | 1.21956375 | Probable E3 ubiquitin-protein ligase BAH1-like 1（BAH1L1） |
| *Seita.9G169700* | -1.044409904 | Zinc finger protein 1（ZFP1） |
| *Seita.9G190200* | -1.127961228 | AAA-ATPase At2g46620 |
| *Seita.9G196800* | -1.007017749 | 21 kDa protein |
| *Seita.9G197700* | -1.894129287 | Unknown |
| *Seita.9G197900* | -1.877782107 | Unknown |
| *Seita.9G217600* | 1.190333816 | Probable LRR receptor-like serine/threonine-protein kinase At1g56140 |
| *Seita.9G217700* | 1.443316081 | Cysteine-rich receptor-like protein kinase 42（CRK42） |
| *Seita.9G224200* | 1.26101179 | Long-chain-alcohol oxidase （FAO1） |
| *Seita.9G228200* | -1.380853905 | Unknown |
| *Seita.9G228500* | -1.147169402 | Unknown |
| *Seita.9G320700* | -1.519649619 | Ethylene-responsive transcription factor （ERF105） |
| *Seita.9G323300* | -2.154040556 | Ethylene-responsive transcription factor （ERF034） |
| *Seita.9G327900* | 1.209694109 | Protein NRT1/ PTR family 6.3（NPF6.3） |
| *Seita.9G328000* | 1.511519886 | Protein NRT1/ PTR family 6.3（NPF6.3） |
| *Seita.9G340400* | -2.067026585 | Protein EXORDIUM-like 5（EXL5） |
| *Seita.9G358800* | 2.380141494 | Probable indole-3-acetic acid-amido synthetase （GH3.8） |
| *Seita.9G400900* | -1.211234064 | Protein IQ-DOMAIN 14（IQD14） |
| *Seita.9G413800* | -1.259116323 | Unknown |
| *Seita.9G431900* | -2.145263396 | Unknown |
| *Seita.9G434000* | -1.402027895 | COBRA-like protein 7（COBL7） |
| *Seita.9G456100* | -1.134489071 | Uncharacterized TPR repeat-containing protein At1g05150 |
| *Seita.9G471800* | 1.08546222 | Thaumatin-like protein 1（TLP1） |
| *Seita.9G509400* | -1.722294525 | Ethylene-responsive transcription factor （ERF057） |
| *Seita.9G511600* | -1.695972049 | Unknown |
| *Seita.9G524700* | -2.130415809 | Unknown |
| *Seita.9G537500* | -1.407007036 | Heavy metal-associated isoprenylated plant protein 5（HIPP05） |
| *Seita.9G564500* | -1.104898711 | Subtilisin-like protease 4（SBTM4） |
|  |  |  |

| Table S5. GO enrichment analysis of differentially expressed genes in foxtail millet after 3 days of PET nanoplastics treatment | | | | | | | | | | |
| --- | --- | --- | --- | --- | --- | --- | --- | --- | --- | --- |
| id | Term | Category | ListHits | ListTotal | PopHits | geneID | | | | |
| GO:0003700 | DNA-binding transcription factor activity | molecular_function | 78 | 442 | 1254 | *Seita.1G025800;Seita.1G043800;Seita.1G067400;Seita.1G236100;Seita.1G248300;Seita.1G253700;Seita.1G259000;Seita.1G259200;Seita.1G271000;Seita.1G301300;Seita.1G308800;Seita.1G331600;Seita.1G376200;Seita.2G140900;Seita.2G222900;Seita.2G228700;Seita.2G242400;Seita.2G263000;Seita.2G280000;Seita.2G280100;Seita.2G280200;Seita.2G280300;Seita.2G280400;Seita.3G031600;Seita.3G124200;Seita.3G124300;Seita.3G158300;Seita.3G191900;Seita.3G242400;Seita.3G325400;Seita.3G368200;Seita.3G370000;Seita.3G379700;Seita.4G067200;Seita.4G097900;Seita.4G116600;Seita.4G187300;Seita.4G188900;Seita.4G227700;Seita.4G263400;Seita.5G034500;Seita.5G096900;Seita.5G204500;Seita.5G233100;Seita.5G280700;Seita.5G306000;Seita.5G348000;Seita.5G348100;Seita.5G391200;Seita.5G394300;Seita.6G023700;Seita.6G023800;Seita.6G032700;Seita.6G185100;Seita.6G198600;Seita.6G226200;Seita.6G236100;Seita.7G112100;Seita.7G149900;Seita.7G179600;Seita.7G188700;Seita.7G202900;Seita.7G205200;Seita.7G212900;Seita.7G232100;Seita.7G262800;Seita.7G267900;Seita.7G288200;Seita.8G008100;Seita.8G123100;Seita.9G170900;Seita.9G301000;Seita.9G325200;Seita.9G355300;Seita.9G474600;Seita.9G509400;Seita.9G511700;Seita.9G525000* | | | | |
| GO:0010200 | response to chitin | biological_process | 15 | 442 | 128 | *Seita.1G067400;Seita.1G186200;Seita.1G186400;Seita.1G327300;Seita.2G140900;Seita.2G238400;Seita.3G007200;Seita.3G205500;Seita.4G067200;Seita.4G188900;Seita.5G233100;Seita.5G363900;Seita.6G186400;Seita.7G095100;Seita.7G232100* | | | | |
| GO:0005634 | nucleus | cellular_component | 157 | 442 | 4924 | *Seita.1G025800;Seita.1G040800;Seita.1G043800;Seita.1G045800;Seita.1G067400;Seita.1G236100;Seita.1G239000;Seita.1G239600;Seita.1G246000;Seita.1G248300;Seita.1G253700;Seita.1G259000;Seita.1G259200;Seita.1G271000;Seita.1G301300;Seita.1G308800;Seita.1G310800;Seita.1G315700;Seita.1G331600;Seita.1G356800;Seita.1G376200;Seita.2G086600;Seita.2G140900;Seita.2G155300;Seita.2G217500;Seita.2G222100;Seita.2G222900;Seita.2G228700;Seita.2G242400;Seita.2G263000;Seita.2G280000;Seita.2G280100;Seita.2G280200;Seita.2G280300;Seita.2G280400;Seita.2G381600;Seita.3G009900;Seita.3G031600;Seita.3G046400;Seita.3G053800;Seita.3G055700;Seita.3G062400;Seita.3G076200;Seita.3G078700;Seita.3G097900;Seita.3G124200;Seita.3G145200;Seita.3G147500;Seita.3G158300;Seita.3G160800;Seita.3G176500;Seita.3G191900;Seita.3G193800;Seita.3G196600;Seita.3G205500;Seita.3G225200;Seita.3G225500;Seita.3G242400;Seita.3G325400;Seita.3G368200;Seita.3G370000;Seita.3G373500;Seita.3G379500;Seita.3G379700;Seita.3G380200;Seita.4G067200;Seita.4G070400;Seita.4G097900;Seita.4G105600;Seita.4G116600;Seita.4G187300;Seita.4G188900;Seita.4G214500;Seita.4G222700;Seita.4G263400;Seita.4G281200;Seita.5G010800;Seita.5G025200;Seita.5G027400;Seita.5G034500;Seita.5G065300;Seita.5G096900;Seita.5G132900;Seita.5G204500;Seita.5G233100;Seita.5G280700;Seita.5G281400;Seita.5G284300;Seita.5G287300;Seita.5G306000;Seita.5G312700;Seita.5G315500;Seita.5G336800;Seita.5G348000;Seita.5G348100;Seita.5G363900;Seita.5G391200;Seita.5G394300;Seita.6G023700;Seita.6G023800;Seita.6G032700;Seita.6G045900;Seita.6G075700;Seita.6G122900;Seita.6G136200;Seita.6G161100;Seita.6G170000;Seita.6G170200;Seita.6G185100;Seita.6G198600;Seita.6G226200;Seita.6G236100;Seita.6G239700;Seita.7G090200;Seita.7G112100;Seita.7G135400;Seita.7G140200;Seita.7G149900;Seita.7G169600;Seita.7G179600;Seita.7G188700;Seita.7G202900;Seita.7G205200;Seita.7G212900;Seita.7G232100;Seita.7G262800;Seita.7G267900;Seita.7G288200;Seita.8G008100;Seita.8G123100;Seita.8G219700;Seita.9G039200;Seita.9G067200;Seita.9G079200;Seita.9G112800;Seita.9G170900;Seita.9G216200;Seita.9G234200;Seita.9G253000;Seita.9G301000;Seita.9G325200;Seita.9G349000;Seita.9G355300;Seita.9G369800;Seita.9G417000;Seita.9G432200;Seita.9G437300;Seita.9G440300;Seita.9G453400;Seita.9G465600;Seita.9G474600;Seita.9G500200;Seita.9G509400;Seita.9G511700;Seita.9G512800;Seita.9G525000;Seita.9G567400* | | | | |
| GO:0043565 | sequence-specific DNA binding | molecular_function | 38 | 442 | 757 | *Seita.1G025800;Seita.1G043800;Seita.1G248300;Seita.1G253700;Seita.1G376200;Seita.2G140900;Seita.2G228700;Seita.2G263000;Seita.3G062400;Seita.3G325400;Seita.3G368200;Seita.4G067200;Seita.4G097900;Seita.4G227700;Seita.5G025200;Seita.5G034500;Seita.5G233100;Seita.5G306000;Seita.6G023700;Seita.6G023800;Seita.6G045900;Seita.6G198600;Seita.6G236100;Seita.7G112100;Seita.7G179600;Seita.7G202900;Seita.7G232100;Seita.7G267900;Seita.7G288200;Seita.9G039200;Seita.9G170900;Seita.9G301000;Seita.9G325200;Seita.9G355300;Seita.9G432200;Seita.9G474600;Seita.9G509400;Seita.9G525000* | | | | |
| GO:0006952 | defense response | biological_process | 44 | 442 | 939 | *Seita.1G045800;Seita.1G198500;Seita.1G225300;Seita.1G246000;Seita.1G345000;Seita.2G056400;Seita.2G057000;Seita.2G217500;Seita.2G229000;Seita.2G232900;Seita.2G434600;Seita.3G007200;Seita.3G031600;Seita.3G047600;Seita.3G076300;Seita.3G176500;Seita.3G191900;Seita.3G225200;Seita.3G338000;Seita.3G339800;Seita.3G340200;Seita.3G386800;Seita.4G214500;Seita.5G169500;Seita.5G203400;Seita.5G233100;Seita.5G281400;Seita.5G348000;Seita.5G348100;Seita.6G023100;Seita.6G023700;Seita.6G023800;Seita.6G182600;Seita.6G182700;Seita.7G179400;Seita.7G241600;Seita.8G242600;Seita.8G242800;Seita.8G243800;Seita.8G244000;Seita.8G247600;Seita.9G042400;Seita.9G198200;Seita.9G555000* | | | | |
| GO:0006355 | regulation of transcription, DNA-templated | biological_process | 39 | 442 | 792 | *Seita.1G067400;Seita.1G301300;Seita.1G308800;Seita.1G356800;Seita.1G376200;Seita.2G086600;Seita.2G140900;Seita.2G222900;Seita.2G228700;Seita.2G242400;Seita.2G263000;Seita.3G055700;Seita.3G097900;Seita.3G124200;Seita.3G242400;Seita.3G325400;Seita.3G368200;Seita.3G370000;Seita.3G379500;Seita.4G097900;Seita.4G116600;Seita.4G188900;Seita.5G204500;Seita.5G312700;Seita.5G336800;Seita.6G023700;Seita.6G023800;Seita.6G198600;Seita.6G226200;Seita.7G112100;Seita.7G169600;Seita.7G212900;Seita.7G288200;Seita.9G039200;Seita.9G067200;Seita.9G170900;Seita.9G465600;Seita.9G474600;Seita.9G525000* | | | | |
| GO:0009873 | ethylene-activated signaling pathway | biological_process | 17 | 442 | 215 | *Seita.1G248300;Seita.1G259000;Seita.1G259200;Seita.3G031600;Seita.3G191900;Seita.4G227700;Seita.5G348000;Seita.5G348100;Seita.5G394300;Seita.6G023700;Seita.6G023800;Seita.6G170000;Seita.6G170200;Seita.7G188700;Seita.7G232100;Seita.7G262800;Seita.9G509400* | | | | |
| GO:0070413 | trehalose metabolism in response to stress | biological_process | 4 | 442 | 10 | *Seita.1G347300;Seita.2G197800;Seita.5G318400;Seita.6G144600* | | | | |
| GO:0044212 | transcription regulatory region DNA binding | molecular_function | 18 | 442 | 271 | *Seita.1G259200;Seita.2G140900;Seita.2G222900;Seita.2G242400;Seita.3G368200;Seita.4G067200;Seita.5G025200;Seita.5G034500;Seita.5G233100;Seita.6G023700;Seita.6G023800;Seita.7G179600;Seita.7G288200;Seita.8G008100;Seita.8G123100;Seita.9G170900;Seita.9G432200;Seita.9G474600* | | | | |
| GO:0016829 | lyase activity | molecular_function | 6 | 442 | 32 | *Seita.2G229000;Seita.2G232900;Seita.6G182600;Seita.6G182700;Seita.6G234900;Seita.8G247600* | | | | |
| GO:0005992 | trehalose biosynthetic process | biological_process | 5 | 442 | 23 | *Seita.1G262000;Seita.1G347300;Seita.2G197800;Seita.5G318400;Seita.6G144600* | | | | |
| GO:0000978 | RNA polymerase II proximal promoter sequence-specific DNA binding | molecular_function | 6 | 442 | 42 | *Seita.1G025800;Seita.6G236100;Seita.7G202900;Seita.7G288200;Seita.8G123100;Seita.9G525000* | | | | |
| GO:0045549 | 9-cis-epoxycarotenoid dioxygenase activity | molecular_function | 3 | 442 | 7 | *Seita.1G288400;Seita.2G035400;Seita.9G156500* | | | | |
| GO:0010436 | carotenoid dioxygenase activity | molecular_function | 3 | 442 | 10 | *Seita.1G288400;Seita.2G035400;Seita.9G156500* | | | | |
| GO:0016121 | carotene catabolic process | biological_process | 3 | 442 | 10 | *Seita.1G288400;Seita.2G035400;Seita.9G156500* | | | | |
| GO:0009738 | abscisic acid-activated signaling pathway | biological_process | 13 | 442 | 221 | *Seita.1G239600;Seita.1G331600;Seita.2G140900;Seita.2G222900;Seita.3G076200;Seita.3G160800;Seita.3G193800;Seita.3G368200;Seita.5G284300;Seita.9G170900;Seita.9G437300;Seita.9G440300;Seita.9G474600* | | | | |
| GO:0009814 | defense response, incompatible interaction | biological_process | 4 | 442 | 24 | *Seita.1G186400;Seita.1G327300;Seita.2G238400;Seita.6G186400* | | | | |
| GO:0016036 | cellular response to phosphate starvation | biological_process | 5 | 442 | 40 | *Seita.2G289600;Seita.4G222700;Seita.6G159400;Seita.9G253000;Seita.9G369800* | | | | |
| GO:0003677 | DNA binding | molecular_function | 48 | 442 | 1401 | *Seita.1G040800;Seita.1G236100;Seita.1G259000;Seita.1G271000;Seita.1G331600;Seita.2G086600;Seita.2G242400;Seita.2G280000;Seita.2G280100;Seita.2G280200;Seita.2G280300;Seita.2G280400;Seita.3G009900;Seita.3G031600;Seita.3G046400;Seita.3G055700;Seita.3G124300;Seita.3G158300;Seita.3G191900;Seita.3G225500;Seita.3G242400;Seita.3G370000;Seita.3G379700;Seita.4G067200;Seita.4G187300;Seita.4G263400;Seita.5G096900;Seita.5G204500;Seita.5G280700;Seita.5G336800;Seita.5G348000;Seita.5G348100;Seita.5G391200;Seita.5G394300;Seita.6G032700;Seita.6G161100;Seita.6G239700;Seita.7G149900;Seita.7G169600;Seita.7G188700;Seita.7G205200;Seita.7G212900;Seita.7G262800;Seita.9G067200;Seita.9G112800;Seita.9G453400;Seita.9G511700;Seita.9G525000* | | | | |
| GO:0048527 | lateral root development | biological_process | 5 | 442 | 44 | *Seita.3G124200;Seita.4G263400;Seita.6G032700;Seita.7G267900;Seita.8G123100* | | | | |
| GO:0010017 | red or far-red light signaling pathway | biological_process | 3 | 442 | 14 | *Seita.1G043800;Seita.1G067400;Seita.4G188900* | | | | |
| GO:0007165 | signal transduction | biological_process | 6 | 442 | 69 | *Seita.2G206200;Seita.2G381600;Seita.2G432000;Seita.3G284500;Seita.6G163200;Seita.7G300300* | | | | |
| GO:0046345 | abscisic acid catabolic process | biological_process | 2 | 442 | 5 | *Seita.1G288100;Seita.2G229900* | | | | |
| GO:0009816 | defense response to bacterium, incompatible interaction | biological_process | 5 | 442 | 52 | *Seita.1G186400;Seita.1G327300;Seita.2G238400;Seita.4G067200;Seita.6G186400* | | | | |
| GO:0005930 | axoneme | cellular_component | 2 | 442 | 6 | *Seita.1G007800;Seita.2G352400* | | | | |
| GO:0030286 | dynein complex | cellular_component | 2 | 442 | 6 | *Seita.1G007800;Seita.2G352400* | | | | |
| GO:0044458 | motile cilium assembly | biological_process | 2 | 442 | 6 | *Seita.1G007800;Seita.2G352400* | | | | |
| GO:0090229 | negative regulation of red or far-red light signaling pathway | biological_process | 2 | 442 | 6 | *Seita.3G055700;Seita.9G067200* | | | | |
| GO:0097014 | ciliary plasm | cellular_component | 2 | 442 | 6 | *Seita.1G007800;Seita.2G352400* | | | | |
| GO:0016787 | hydrolase activity | molecular_function | 11 | 442 | 205 | *Seita.1G310800;Seita.2G229000;Seita.2G232900;Seita.3G340200;Seita.3G386800;Seita.6G014300;Seita.6G182600;Seita.6G182700;Seita.7G209000;Seita.8G247600;Seita.9G500200* | | | | |
| GO:0045892 | negative regulation of transcription, DNA-templated | biological_process | 10 | 442 | 178 | *Seita.1G067400;Seita.2G381600;Seita.3G078700;Seita.3G124200;Seita.4G188900;Seita.5G010800;Seita.6G023700;Seita.6G023800;Seita.7G232100;Seita.9G525000* | | | | |
| GO:0009734 | auxin-activated signaling pathway | biological_process | 13 | 442 | 267 | *Seita.1G317400;Seita.1G356800;Seita.2G295000;Seita.2G302500;Seita.3G379500;Seita.4G263400;Seita.4G266000;Seita.5G312700;Seita.6G032700;Seita.7G169600;Seita.7G212900;Seita.7G230900;Seita.9G512800* | | | | |
| GO:0009607 | response to biotic stimulus | biological_process | 4 | 442 | 37 | *Seita.1G198500;Seita.1G345000;Seita.3G338000;Seita.9G555000* | | | | |
| GO:0030307 | positive regulation of cell growth | biological_process | 3 | 442 | 20 | *Seita.3G124200;Seita.6G170000;Seita.6G170200* | | | | |
| GO:0004540 | ribonuclease activity | molecular_function | 2 | 442 | 7 | *Seita.2G289600;Seita.6G159400* | | | | |
| GO:0019897 | extrinsic component of plasma membrane | cellular_component | 2 | 442 | 7 | *Seita.6G170000;Seita.6G170200* | | | | |
| GO:0033897 | ribonuclease T2 activity | molecular_function | 2 | 442 | 7 | *Seita.2G289600;Seita.6G159400* | | | | |
| GO:0034605 | cellular response to heat | biological_process | 5 | 442 | 59 | *Seita.1G025800;Seita.5G315500;Seita.5G376100;Seita.6G236100;Seita.7G202900* | | | | |
| GO:0009688 | abscisic acid biosynthetic process | biological_process | 3 | 442 | 21 | *Seita.1G288400;Seita.2G035400;Seita.9G156500* | | | | |
| GO:0010427 | abscisic acid binding | molecular_function | 2 | 442 | 8 | *Seita.3G076200;Seita.9G437300* | | | | |
| GO:0031514 | motile cilium | cellular_component | 2 | 442 | 8 | *Seita.1G007800;Seita.2G352400* | | | | |
| GO:0080163 | regulation of protein serine/threonine phosphatase activity | biological_process | 2 | 442 | 8 | *Seita.3G076200;Seita.9G437300* | | | | |
| GO:0009846 | pollen germination | biological_process | 5 | 442 | 64 | *Seita.1G315700;Seita.1G349700;Seita.3G373500;Seita.4G105600;Seita.7G140200* | | | | |
| GO:0016311 | dephosphorylation | biological_process | 3 | 442 | 24 | *Seita.1G347300;Seita.5G318400;Seita.6G144600* | | | | |
| GO:0019538 | protein metabolic process | biological_process | 3 | 442 | 24 | *Seita.5G315500;Seita.7G296600;Seita.8G037700* | | | | |
| GO:0030674 | protein binding, bridging | molecular_function | 3 | 442 | 24 | *Seita.2G325500;Seita.8G049400;Seita.9G552100* | | | | |
| GO:0000095 | S-adenosyl-L-methionine transmembrane transporter activity | molecular_function | 2 | 442 | 9 | *Seita.9G254900;Seita.9G520900* | | | | |
| GO:0010438 | cellular response to sulfur starvation | biological_process | 2 | 442 | 9 | *Seita.1G099500;Seita.3G186600* | | | | |
| GO:0009737 | response to abscisic acid | biological_process | 15 | 442 | 358 | *Seita.1G186400;Seita.1G348600;Seita.2G140900;Seita.3G124200;Seita.3G160800;Seita.3G193800;Seita.4G097900;Seita.4G227700;Seita.5G284300;Seita.6G023700;Seita.6G023800;Seita.7G232100;Seita.9G050000;Seita.9G238100;Seita.9G440300* | | | | |
| GO:0004722 | protein serine/threonine phosphatase activity | molecular_function | 6 | 442 | 91 | *Seita.1G203100;Seita.2G177500;Seita.3G139000;Seita.3G218800;Seita.5G379400;Seita.9G440500* | | | | |
| GO:0051087 | chaperone binding | molecular_function | 4 | 442 | 46 | *Seita.3G147500;Seita.4G100500;Seita.8G128400;Seita.9G440100* | | | | |
| GO:0019760 | glucosinolate metabolic process | biological_process | 3 | 442 | 26 | *Seita.3G368200;Seita.9G170900;Seita.9G474600* | | | | |
| GO:0004575 | sucrose alpha-glucosidase activity | molecular_function | 2 | 442 | 10 | *Seita.1G004600;Seita.5G157600* | | | | |
| GO:0019139 | cytokinin dehydrogenase activity | molecular_function | 2 | 442 | 10 | *Seita.6G174400;Seita.9G217200* | | | | |
| GO:0009788 | negative regulation of abscisic acid-activated signaling pathway | biological_process | 4 | 442 | 47 | *Seita.2G222900;Seita.3G368200;Seita.9G170900;Seita.9G474600* | | | | |
| GO:0009733 | response to auxin | biological_process | 9 | 442 | 180 | *Seita.1G117500;Seita.1G356800;Seita.2G140900;Seita.2G302500;Seita.3G124200;Seita.5G034500;Seita.5G312700;Seita.6G170000;Seita.6G170200* | | | | |
| GO:0009785 | blue light signaling pathway | biological_process | 2 | 442 | 11 | *Seita.1G067400;Seita.4G188900* | | | | |
| GO:0010072 | primary shoot apical meristem specification | biological_process | 2 | 442 | 11 | *Seita.4G263400;Seita.6G032700* | | | | |
| GO:0061408 | positive regulation of transcription from RNA polymerase II promoter in response to heat stress | biological_process | 3 | 442 | 28 | *Seita.1G025800;Seita.6G236100;Seita.7G202900* | | | | |
| GO:0007623 | circadian rhythm | biological_process | 5 | 442 | 73 | *Seita.1G117500;Seita.1G236100;Seita.1G301300;Seita.5G067800;Seita.7G212900* | | | | |
| GO:0004709 | MAP kinase kinase kinase activity | molecular_function | 3 | 442 | 29 | *Seita.3G160800;Seita.5G284300;Seita.9G440300* | | | | |
| GO:0015035 | protein disulfide oxidoreductase activity | molecular_function | 5 | 442 | 75 | *Seita.3G344000;Seita.5G137500;Seita.5G167100;Seita.8G219700;Seita.9G051600* | | | | |
| GO:0004805 | trehalose-phosphatase activity | molecular_function | 2 | 442 | 12 | *Seita.1G262000;Seita.2G197800* | | | | |
| GO:0005199 | structural constituent of cell wall | molecular_function | 2 | 442 | 12 | *Seita.2G050800;Seita.3G105100* | | | | |
| GO:0005504 | fatty acid binding | molecular_function | 2 | 442 | 12 | *Seita.1G339600;Seita.2G106500* | | | | |
| GO:0009696 | salicylic acid metabolic process | biological_process | 2 | 442 | 12 | *Seita.5G443100;Seita.5G443200* | | | | |
| GO:0047501 | (+)-neomenthol dehydrogenase activity | molecular_function | 2 | 442 | 12 | *Seita.7G179400;Seita.9G042400* | | | | |
| GO:0051787 | misfolded protein binding | molecular_function | 2 | 442 | 12 | *Seita.2G401300;Seita.5G376100* | | | | |
| GO:0071482 | cellular response to light stimulus | biological_process | 2 | 442 | 12 | *Seita.3G124300;Seita.5G067800* | | | | |
| GO:0080032 | methyl jasmonate esterase activity | molecular_function | 2 | 442 | 12 | *Seita.5G443100;Seita.5G443200* | | | | |
| GO:0045454 | cell redox homeostasis | biological_process | 7 | 442 | 132 | *Seita.2G327500;Seita.2G436600;Seita.3G344000;Seita.5G137500;Seita.5G167100;Seita.8G219700;Seita.9G051600* | | | | |
| GO:0004190 | aspartic-type endopeptidase activity | molecular_function | 6 | 442 | 104 | *Seita.3G123800;Seita.3G183000;Seita.3G330700;Seita.4G040400;Seita.5G214300;Seita.9G461800* | | | | |
| GO:0009414 | response to water deprivation | biological_process | 12 | 442 | 289 | *Seita.1G331600;Seita.2G140900;Seita.2G291500;Seita.3G007200;Seita.3G076200;Seita.3G193800;Seita.4G070400;Seita.4G227700;Seita.5G336800;Seita.6G023700;Seita.6G023800;Seita.9G050000* | | | | |
| GO:0080031 | methyl salicylate esterase activity | molecular_function | 2 | 442 | 13 | *Seita.5G443100;Seita.5G443200* | | | | |
| GO:0019005 | SCF ubiquitin ligase complex | cellular_component | 4 | 442 | 55 | *Seita.1G182300;Seita.4G070400;Seita.4G226700;Seita.9G512800* | | | | |
| GO:0004864 | protein phosphatase inhibitor activity | molecular_function | 2 | 442 | 14 | *Seita.3G076200;Seita.9G437300* | | | | |
| GO:0008356 | asymmetric cell division | biological_process | 2 | 442 | 14 | *Seita.4G197200;Seita.9G355300* | | | | |
| GO:0009685 | gibberellin metabolic process | biological_process | 2 | 442 | 14 | *Seita.4G015600;Seita.5G147400* | | | | |
| GO:0009834 | plant-type secondary cell wall biogenesis | biological_process | 4 | 442 | 57 | *Seita.1G259000;Seita.7G188700;Seita.7G274700;Seita.8G122400* | | | | |
| GO:0070417 | cellular response to cold | biological_process | 3 | 442 | 34 | *Seita.4G222700;Seita.9G253000;Seita.9G369800* | | | | |
| GO:0000302 | response to reactive oxygen species | biological_process | 4 | 442 | 58 | *Seita.1G117500;Seita.1G342400;Seita.5G092600;Seita.6G210000* | | | | |
| GO:0005985 | sucrose metabolic process | biological_process | 2 | 442 | 15 | *Seita.1G004600;Seita.9G436600* | | | | |
| GO:0080030 | methyl indole-3-acetate esterase activity | molecular_function | 2 | 442 | 15 | *Seita.5G443100;Seita.5G443200* | | | | |
| GO:0042546 | cell wall biogenesis | biological_process | 4 | 442 | 61 | *Seita.1G294300;Seita.2G313800;Seita.4G246200;Seita.9G005800* | | | | |
| GO:0009690 | cytokinin metabolic process | biological_process | 2 | 442 | 16 | *Seita.6G174400;Seita.9G217200* | | | | |
| GO:0010214 | seed coat development | biological_process | 2 | 442 | 16 | *Seita.5G034500;Seita.9G081900* | | | | |
| GO:0016762 | xyloglucan:xyloglucosyl transferase activity | molecular_function | 3 | 442 | 37 | *Seita.2G313800;Seita.4G246200;Seita.9G005800* | | | | |
| GO:0051085 | chaperone cofactor-dependent protein refolding | biological_process | 3 | 442 | 37 | *Seita.3G147500;Seita.5G376100;Seita.9G440100* | | | | |
| GO:0008233 | peptidase activity | molecular_function | 3 | 442 | 38 | *Seita.1G362500;Seita.9G137200;Seita.9G461800* | | | | |
| GO:0009718 | anthocyanin-containing compound biosynthetic process | biological_process | 3 | 442 | 38 | *Seita.2G289600;Seita.6G159400;Seita.7G319100* | | | | |
| GO:0002229 | defense response to oomycetes | biological_process | 2 | 442 | 17 | *Seita.2G001400;Seita.3G047600* | | | | |
| GO:0038023 | signaling receptor activity | molecular_function | 2 | 442 | 17 | *Seita.3G076200;Seita.9G437300* | | | | |
| GO:0009416 | response to light stimulus | biological_process | 7 | 442 | 152 | *Seita.1G239600;Seita.1G362000;Seita.4G070400;Seita.4G225400;Seita.5G027400;Seita.7G267900;Seita.9G325200* | | | | |
| GO:0010026 | trichome differentiation | biological_process | 2 | 442 | 18 | *Seita.2G222900;Seita.2G236200* | | | | |
| GO:0031969 | chloroplast membrane | cellular_component | 7 | 442 | 156 | *Seita.2G053300;Seita.2G116100;Seita.5G058800;Seita.6G110100;Seita.7G209000;Seita.9G254900;Seita.9G520900* | | | | |
| GO:0016125 | sterol metabolic process | biological_process | 3 | 442 | 41 | *Seita.2G094000;Seita.2G229900;Seita.8G027000* | | | | |
| GO:0004521 | endoribonuclease activity | molecular_function | 2 | 442 | 19 | *Seita.2G289600;Seita.6G159400* | | | | |
| GO:0045491 | xylan metabolic process | biological_process | 2 | 442 | 19 | *Seita.7G274700;Seita.8G122400* | | | | |
| GO:0048579 | negative regulation of long-day photoperiodism, flowering | biological_process | 3 | 442 | 42 | *Seita.1G301300;Seita.3G370000;Seita.6G045900* | | | | |
| GO:0003774 | motor activity | molecular_function | 2 | 442 | 20 | *Seita.1G007800;Seita.2G352400* | | | | |
| GO:0005354 | galactose transmembrane transporter activity | molecular_function | 2 | 442 | 20 | *Seita.9G504800;Seita.9G505000* | | | | |
| GO:0005365 | myo-inositol transmembrane transporter activity | molecular_function | 2 | 442 | 20 | *Seita.9G504800;Seita.9G505000* | | | | |
| GO:0015148 | D-xylose transmembrane transporter activity | molecular_function | 2 | 442 | 20 | *Seita.9G504800;Seita.9G505000* | | | | |
| GO:0015168 | glycerol transmembrane transporter activity | molecular_function | 2 | 442 | 20 | *Seita.9G504800;Seita.9G505000* | | | | |
| GO:0015575 | mannitol transmembrane transporter activity | molecular_function | 2 | 442 | 20 | *Seita.9G504800;Seita.9G505000* | | | | |
| GO:0015576 | sorbitol transmembrane transporter activity | molecular_function | 2 | 442 | 20 | *Seita.9G504800;Seita.9G505000* | | | | |
| GO:0015591 | D-ribose transmembrane transporter activity | molecular_function | 2 | 442 | 20 | *Seita.9G504800;Seita.9G505000* | | | | |
| GO:0015145 | monosaccharide transmembrane transporter activity | molecular_function | 2 | 442 | 21 | *Seita.9G504800;Seita.9G505000* | | | | |
| GO:0032586 | protein storage vacuole membrane | cellular_component | 2 | 442 | 21 | *Seita.5G442200;Seita.7G014100* | | | | |
| GO:0004553 | hydrolase activity, hydrolyzing O-glycosyl compounds | molecular_function | 3 | 442 | 46 | *Seita.2G313800;Seita.4G246200;Seita.9G005800* | | | | |
| GO:0006071 | glycerol metabolic process | biological_process | 2 | 442 | 22 | *Seita.1G171900;Seita.5G327000* | | | | |
| GO:0031098 | stress-activated protein kinase signaling cascade | biological_process | 3 | 442 | 47 | *Seita.3G160800;Seita.5G284300;Seita.9G440300* | | | | |
| GO:0042542 | response to hydrogen peroxide | biological_process | 4 | 442 | 75 | *Seita.1G117500;Seita.1G342400;Seita.4G067200;Seita.5G092600* | | | | |
| GO:0006817 | phosphate ion transport | biological_process | 2 | 442 | 23 | *Seita.1G216200;Seita.7G032900* | | | | |
| GO:0007017 | microtubule-based process | biological_process | 2 | 442 | 23 | *Seita.1G007800;Seita.2G352400* | | | | |
| GO:0016998 | cell wall macromolecule catabolic process | biological_process | 2 | 442 | 23 | *Seita.1G225300;Seita.2G313800* | | | | |
| GO:0005618 | cell wall | cellular_component | 15 | 442 | 456 | *Seita.1G045800;Seita.2G050800;Seita.2G289600;Seita.2G313800;Seita.3G028500;Seita.3G105100;Seita.3G225200;Seita.4G003100;Seita.4G176600;Seita.4G246200;Seita.6G159400;Seita.8G028400;Seita.8G056100;Seita.9G005800;Seita.9G081900* | | | | |
| GO:0009651 | response to salt stress | biological_process | 14 | 442 | 420 | *Seita.1G099500;Seita.1G248300;Seita.1G262000;Seita.1G331600;Seita.1G342400;Seita.2G140900;Seita.3G076200;Seita.3G124200;Seita.3G193800;Seita.4G015600;Seita.4G138500;Seita.5G092600;Seita.9G050000;Seita.9G238100* | | | | |
| GO:0009640 | photomorphogenesis | biological_process | 3 | 442 | 49 | *Seita.1G067400;Seita.3G097900;Seita.4G188900* | | | | |
| GO:0045893 | positive regulation of transcription, DNA-templated | biological_process | 10 | 442 | 278 | *Seita.1G248300;Seita.2G228700;Seita.4G067200;Seita.4G227700;Seita.5G394300;Seita.6G045900;Seita.7G179600;Seita.8G008100;Seita.9G067200;Seita.9G325200* | | | | |
| GO:0055114 | oxidation-reduction process | biological_process | 6 | 442 | 143 | *Seita.2G094000;Seita.2G229900;Seita.2G436600;Seita.3G339800;Seita.8G027000;Seita.9G042400* | | | | |
| GO:0006829 | zinc ion transport | biological_process | 1 | 442 | 5 | *Seita.2G090600* | | | | |
| GO:0010018 | far-red light signaling pathway | biological_process | 1 | 442 | 5 | *Seita.7G179600* | | | | |
| GO:0010482 | regulation of epidermal cell division | biological_process | 1 | 442 | 5 | *Seita.2G236200* | | | | |
| GO:0010601 | positive regulation of auxin biosynthetic process | biological_process | 1 | 442 | 5 | *Seita.3G048500* | | | | |
| GO:0031210 | phosphatidylcholine binding | molecular_function | 1 | 442 | 5 | *Seita.9G306400* | | | | |
| GO:0045038 | protein import into chloroplast thylakoid membrane | biological_process | 1 | 442 | 5 | *Seita.9G552100* | | | | |
| GO:0048317 | seed morphogenesis | biological_process | 1 | 442 | 5 | *Seita.8G008100* | | | | |
| GO:0048531 | beta-1,3-galactosyltransferase activity | molecular_function | 1 | 442 | 5 | *Seita.1G294300* | | | | |
| GO:0080006 | internode patterning | biological_process | 1 | 442 | 5 | *Seita.9G067200* | | | | |
| GO:0080132 | fatty acid alpha-hydroxylase activity | molecular_function | 1 | 442 | 5 | *Seita.8G027000* | | | | |
| GO:0098807 | chloroplast thylakoid membrane protein complex | cellular_component | 1 | 442 | 5 | *Seita.5G067800* | | | | |
| GO:1901430 | positive regulation of syringal lignin biosynthetic process | biological_process | 1 | 442 | 5 | *Seita.4G176600* | | | | |
| GO:1905183 | negative regulation of protein serine/threonine phosphatase activity | biological_process | 1 | 442 | 5 | *Seita.3G076200* | | | | |
| GO:0006401 | RNA catabolic process | biological_process | 2 | 442 | 25 | *Seita.2G289600;Seita.6G159400* | | | | |
| GO:0010431 | seed maturation | biological_process | 2 | 442 | 25 | *Seita.6G210000;Seita.9G050000* | | | | |
| GO:0031349 | positive regulation of defense response | biological_process | 2 | 442 | 25 | *Seita.1G246000;Seita.5G058800* | | | | |
| GO:1902584 | positive regulation of response to water deprivation | biological_process | 2 | 442 | 25 | *Seita.4G070400;Seita.9G039200* | | | | |
| GO:0042744 | hydrogen peroxide catabolic process | biological_process | 7 | 442 | 179 | *Seita.1G022500;Seita.1G117500;Seita.4G176600;Seita.6G210000;Seita.9G298200;Seita.9G562600;Seita.J003100* | | | | |
| GO:0023014 | signal transduction by protein phosphorylation | biological_process | 3 | 442 | 52 | *Seita.3G160800;Seita.5G284300;Seita.9G440300* | | | | |
| GO:0004674 | protein serine/threonine kinase activity | molecular_function | 17 | 442 | 544 | *Seita.1G239600;Seita.1G246000;Seita.2G206200;Seita.2G432000;Seita.3G053800;Seita.3G083300;Seita.3G160800;Seita.3G284500;Seita.3G380200;Seita.4G232700;Seita.5G203200;Seita.5G281400;Seita.5G284300;Seita.6G163200;Seita.7G300300;Seita.8G022900;Seita.9G440300* | | | | |
| GO:0051453 | regulation of intracellular pH | biological_process | 2 | 442 | 26 | *Seita.2G251700;Seita.4G138500* | | | | |
| GO:0020037 | heme binding | molecular_function | 16 | 442 | 508 | *Seita.1G022500;Seita.1G117500;Seita.1G288100;Seita.2G094000;Seita.2G229900;Seita.2G404300;Seita.3G339800;Seita.4G176600;Seita.6G023100;Seita.6G210000;Seita.7G323700;Seita.8G027000;Seita.9G298200;Seita.9G469300;Seita.9G562600;Seita.J003100* | | | | |
| GO:0071555 | cell wall organization | biological_process | 12 | 442 | 360 | *Seita.1G045800;Seita.1G054000;Seita.1G268900;Seita.1G294300;Seita.2G050800;Seita.2G313800;Seita.3G105100;Seita.3G225200;Seita.4G246200;Seita.5G203400;Seita.5G303500;Seita.9G005800* | | | | |
| GO:0051082 | unfolded protein binding | molecular_function | 6 | 442 | 149 | *Seita.1G342400;Seita.3G147500;Seita.5G092600;Seita.5G376100;Seita.6G200300;Seita.9G440100* | | | | |
| GO:0048544 | recognition of pollen | biological_process | 2 | 442 | 27 | *Seita.3G083300;Seita.4G232700* | | | | |
| GO:0008270 | zinc ion binding | molecular_function | 17 | 442 | 552 | *Seita.1G067400;Seita.1G301300;Seita.1G308800;Seita.2G106500;Seita.2G155300;Seita.2G242400;Seita.3G097900;Seita.3G124200;Seita.4G116600;Seita.4G188900;Seita.6G189200;Seita.6G226200;Seita.6G235000;Seita.7G267900;Seita.9G234200;Seita.9G325200;Seita.9G465600* | | | | |
| GO:0002215 | defense response to nematode | biological_process | 1 | 442 | 6 | *Seita.9G298200* | | | | |
| GO:0004345 | glucose-6-phosphate dehydrogenase activity | molecular_function | 1 | 442 | 6 | *Seita.9G365500* | | | | |
| GO:0004435 | phosphatidylinositol phospholipase C activity | molecular_function | 1 | 442 | 6 | *Seita.3G048500* | | | | |
| GO:0005816 | spindle pole body | cellular_component | 1 | 442 | 6 | *Seita.2G335500* | | | | |
| GO:0010500 | transmitting tissue development | biological_process | 1 | 442 | 6 | *Seita.2G228700* | | | | |
| GO:0016987 | sigma factor activity | molecular_function | 1 | 442 | 6 | *Seita.3G124300* | | | | |
| GO:0030010 | establishment of cell polarity | biological_process | 1 | 442 | 6 | *Seita.6G198600* | | | | |
| GO:0043433 | negative regulation of DNA-binding transcription factor activity | biological_process | 1 | 442 | 6 | *Seita.3G176500* | | | | |
| GO:0047701 | beta-L-arabinosidase activity | molecular_function | 1 | 442 | 6 | *Seita.2G415300* | | | | |
| GO:0051512 | positive regulation of unidimensional cell growth | biological_process | 1 | 442 | 6 | *Seita.6G045900* | | | | |
| GO:0051567 | histone H3-K9 methylation | biological_process | 1 | 442 | 6 | *Seita.2G236200* | | | | |
| GO:0098869 | cellular oxidant detoxification | biological_process | 1 | 442 | 6 | *Seita.1G099500* | | | | |
| GO:0032147 | activation of protein kinase activity | biological_process | 3 | 442 | 55 | *Seita.3G160800;Seita.5G284300;Seita.9G440300* | | | | |
| GO:0006457 | protein folding | biological_process | 6 | 442 | 151 | *Seita.1G342400;Seita.4G100500;Seita.5G092600;Seita.6G184400;Seita.6G200300;Seita.8G128400* | | | | |
| GO:0008061 | chitin binding | molecular_function | 2 | 442 | 28 | *Seita.1G225300;Seita.8G152800* | | | | |
| GO:0010029 | regulation of seed germination | biological_process | 2 | 442 | 28 | *Seita.3G193800;Seita.4G097900* | | | | |
| GO:0051259 | protein complex oligomerization | biological_process | 2 | 442 | 28 | *Seita.1G342400;Seita.5G092600* | | | | |
| GO:0010411 | xyloglucan metabolic process | biological_process | 3 | 442 | 57 | *Seita.2G313800;Seita.4G246200;Seita.9G005800* | | | | |
| GO:0009686 | gibberellin biosynthetic process | biological_process | 2 | 442 | 29 | *Seita.4G015600;Seita.5G147400* | | | | |
| GO:0009694 | jasmonic acid metabolic process | biological_process | 2 | 442 | 30 | *Seita.5G443100;Seita.5G443200* | | | | |
| GO:0010268 | brassinosteroid homeostasis | biological_process | 2 | 442 | 30 | *Seita.2G094000;Seita.8G027000* | | | | |
| GO:0016132 | brassinosteroid biosynthetic process | biological_process | 2 | 442 | 30 | *Seita.2G094000;Seita.8G027000* | | | | |
| GO:0000822 | inositol hexakisphosphate binding | molecular_function | 1 | 442 | 7 | *Seita.9G512800* | | | | |
| GO:0003883 | CTP synthase activity | molecular_function | 1 | 442 | 7 | *Seita.3G138700* | | | | |
| GO:0004096 | catalase activity | molecular_function | 1 | 442 | 7 | *Seita.1G117500* | | | | |
| GO:0006656 | phosphatidylcholine biosynthetic process | biological_process | 1 | 442 | 7 | *Seita.9G306400* | | | | |
| GO:0008265 | Mo-molybdopterin cofactor sulfurase activity | molecular_function | 1 | 442 | 7 | *Seita.6G234900* | | | | |
| GO:0009942 | longitudinal axis specification | biological_process | 1 | 442 | 7 | *Seita.6G198600* | | | | |
| GO:0010011 | auxin binding | molecular_function | 1 | 442 | 7 | *Seita.9G512800* | | | | |
| GO:0010187 | negative regulation of seed germination | biological_process | 1 | 442 | 7 | *Seita.4G070400* | | | | |
| GO:0015114 | phosphate ion transmembrane transporter activity | molecular_function | 1 | 442 | 7 | *Seita.1G231800* | | | | |
| GO:0035627 | ceramide transport | biological_process | 1 | 442 | 7 | *Seita.7G105000* | | | | |
| GO:0044210 | 'de novo' CTP biosynthetic process | biological_process | 1 | 442 | 7 | *Seita.3G138700* | | | | |
| GO:0047274 | galactinol-sucrose galactosyltransferase activity | molecular_function | 1 | 442 | 7 | *Seita.6G195300* | | | | |
| GO:0051300 | spindle pole body organization | biological_process | 1 | 442 | 7 | *Seita.3G196600* | | | | |
| GO:0071629 | cytoplasm protein quality control by the ubiquitin-proteasome system | biological_process | 1 | 442 | 7 | *Seita.2G401300* | | | | |
| GO:0080060 | integument development | biological_process | 1 | 442 | 7 | *Seita.8G008100* | | | | |
| GO:0080079 | cellobiose glucosidase activity | molecular_function | 1 | 442 | 7 | *Seita.2G415300* | | | | |
| GO:0102867 | molybdenum cofactor sulfurtransferase activity | molecular_function | 1 | 442 | 7 | *Seita.6G234900* | | | | |
| GO:0120009 | intermembrane lipid transfer | biological_process | 1 | 442 | 7 | *Seita.7G105000* | | | | |
| GO:0120013 | intermembrane lipid transfer activity | molecular_function | 1 | 442 | 7 | *Seita.7G105000* | | | | |
| GO:1900036 | positive regulation of cellular response to heat | biological_process | 1 | 442 | 7 | *Seita.1G248300* | | | | |
| GO:1902387 | ceramide 1-phosphate binding | molecular_function | 1 | 442 | 7 | *Seita.7G105000* | | | | |
| GO:1902388 | ceramide 1-phosphate transporter activity | molecular_function | 1 | 442 | 7 | *Seita.7G105000* | | | | |
| GO:0016722 | oxidoreductase activity, oxidizing metal ions | molecular_function | 3 | 442 | 60 | *Seita.4G003100;Seita.5G382800;Seita.8G212900* | | | | |
| GO:0005315 | inorganic phosphate transmembrane transporter activity | molecular_function | 2 | 442 | 31 | *Seita.1G216200;Seita.7G032900* | | | | |
| GO:0010089 | xylem development | biological_process | 2 | 442 | 31 | *Seita.4G176600;Seita.5G034500* | | | | |
| GO:0102336 | 3-oxo-arachidoyl-CoA synthase activity | molecular_function | 2 | 442 | 31 | *Seita.1G362000;Seita.4G225400* | | | | |
| GO:0102337 | 3-oxo-cerotoyl-CoA synthase activity | molecular_function | 2 | 442 | 31 | *Seita.1G362000;Seita.4G225400* | | | | |
| GO:0102338 | 3-oxo-lignoceronyl-CoA synthase activity | molecular_function | 2 | 442 | 31 | *Seita.1G362000;Seita.4G225400* | | | | |
| GO:0102756 | very-long-chain 3-ketoacyl-CoA synthase activity | molecular_function | 2 | 442 | 31 | *Seita.1G362000;Seita.4G225400* | | | | |
| GO:0035556 | intracellular signal transduction | biological_process | 5 | 442 | 127 | *Seita.1G239600;Seita.3G048500;Seita.3G053800;Seita.3G145200;Seita.3G380200* | | | | |
| GO:0006417 | regulation of translation | biological_process | 3 | 442 | 62 | *Seita.5G312700;Seita.9G004600;Seita.9G461500* | | | | |
| GO:0003978 | UDP-glucose 4-epimerase activity | molecular_function | 1 | 442 | 8 | *Seita.2G282600* | | | | |
| GO:0004792 | thiosulfate sulfurtransferase activity | molecular_function | 1 | 442 | 8 | *Seita.1G001900* | | | | |
| GO:0005471 | ATP:ADP antiporter activity | molecular_function | 1 | 442 | 8 | *Seita.3G164400* | | | | |
| GO:0008515 | sucrose transmembrane transporter activity | molecular_function | 1 | 442 | 8 | *Seita.9G436600* | | | | |
| GO:0009956 | radial pattern formation | biological_process | 1 | 442 | 8 | *Seita.9G355300* | | | | |
| GO:0015140 | malate transmembrane transporter activity | molecular_function | 1 | 442 | 8 | *Seita.2G251700* | | | | |
| GO:0015175 | neutral amino acid transmembrane transporter activity | molecular_function | 1 | 442 | 8 | *Seita.1G268000* | | | | |
| GO:0016208 | AMP binding | molecular_function | 1 | 442 | 8 | *Seita.2G428900* | | | | |
| GO:0018685 | alkane 1-monooxygenase activity | molecular_function | 1 | 442 | 8 | *Seita.7G323700* | | | | |
| GO:0019344 | cysteine biosynthetic process | biological_process | 1 | 442 | 8 | *Seita.2G327500* | | | | |
| GO:0030100 | regulation of endocytosis | biological_process | 1 | 442 | 8 | *Seita.2G434600* | | | | |
| GO:0033926 | glycopeptide alpha-N-acetylgalactosaminidase activity | molecular_function | 1 | 442 | 8 | *Seita.5G157600* | | | | |
| GO:0034613 | cellular protein localization | biological_process | 1 | 442 | 8 | *Seita.2G335500* | | | | |
| GO:0044209 | AMP salvage | biological_process | 1 | 442 | 8 | *Seita.1G231800* | | | | |
| GO:0052634 | C-19 gibberellin 2-beta-dioxygenase activity | molecular_function | 1 | 442 | 8 | *Seita.5G147400* | | | | |
| GO:0090697 | post-embryonic plant organ morphogenesis | biological_process | 1 | 442 | 8 | *Seita.3G124200* | | | | |
| GO:0102229 | amylopectin maltohydrolase activity | molecular_function | 1 | 442 | 8 | *Seita.9G229700* | | | | |
| GO:1901601 | strigolactone biosynthetic process | biological_process | 1 | 442 | 8 | *Seita.9G500200* | | | | |
| GO:2000469 | negative regulation of peroxidase activity | biological_process | 1 | 442 | 8 | *Seita.3G124200* | | | | |
| GO:0004842 | ubiquitin-protein transferase activity | molecular_function | 10 | 442 | 310 | *Seita.1G186200;Seita.1G186400;Seita.1G327300;Seita.2G238400;Seita.3G007200;Seita.3G205500;Seita.5G363900;Seita.6G186400;Seita.7G095100;Seita.9G238100* | | | | |
| GO:0045492 | xylan biosynthetic process | biological_process | 2 | 442 | 33 | *Seita.7G274700;Seita.8G122400* | | | | |
| GO:0006633 | fatty acid biosynthetic process | biological_process | 4 | 442 | 96 | *Seita.1G362000;Seita.3G196200;Seita.4G031400;Seita.4G225400* | | | | |
| GO:0016757 | transferase activity, transferring glycosyl groups | molecular_function | 4 | 442 | 97 | *Seita.1G347300;Seita.5G318400;Seita.6G144600;Seita.9G542400* | | | | |
| GO:0051865 | protein autoubiquitination | biological_process | 2 | 442 | 34 | *Seita.2G401300;Seita.3G007200* | | | | |
| GO:0009926 | auxin polar transport | biological_process | 3 | 442 | 65 | *Seita.1G317400;Seita.2G295000;Seita.4G070400* | | | | |
| GO:0000285 | 1-phosphatidylinositol-3-phosphate 5-kinase activity | molecular_function | 1 | 442 | 9 | *Seita.2G200700* | | | | |
| GO:0005775 | vacuolar lumen | cellular_component | 1 | 442 | 9 | *Seita.1G004600* | | | | |
| GO:0006552 | leucine catabolic process | biological_process | 1 | 442 | 9 | *Seita.3G382100* | | | | |
| GO:0007338 | single fertilization | biological_process | 1 | 442 | 9 | *Seita.1G349700* | | | | |
| GO:0009266 | response to temperature stimulus | biological_process | 1 | 442 | 9 | *Seita.3G370000* | | | | |
| GO:0009509 | chromoplast | cellular_component | 1 | 442 | 9 | *Seita.4G075200* | | | | |
| GO:0010016 | shoot system morphogenesis | biological_process | 1 | 442 | 9 | *Seita.4G070400* | | | | |
| GO:0015385 | sodium:proton antiporter activity | molecular_function | 1 | 442 | 9 | *Seita.4G138500* | | | | |
| GO:0016297 | acyl-[acyl-carrier-protein] hydrolase activity | molecular_function | 1 | 442 | 9 | *Seita.4G031400* | | | | |
| GO:0016688 | L-ascorbate peroxidase activity | molecular_function | 1 | 442 | 9 | *Seita.6G210000* | | | | |
| GO:0016985 | mannan endo-1,4-beta-mannosidase activity | molecular_function | 1 | 442 | 9 | *Seita.5G260400* | | | | |
| GO:0045145 | single-stranded DNA 5'-3' exodeoxyribonuclease activity | molecular_function | 1 | 442 | 9 | *Seita.3G046400* | | | | |
| GO:0046355 | mannan catabolic process | biological_process | 1 | 442 | 9 | *Seita.5G260400* | | | | |
| GO:0048830 | adventitious root development | biological_process | 1 | 442 | 9 | *Seita.1G317400* | | | | |
| GO:0052739 | phosphatidylserine 1-acylhydrolase activity | molecular_function | 1 | 442 | 9 | *Seita.5G058800* | | | | |
| GO:0052740 | 1-acyl-2-lysophosphatidylserine acylhydrolase activity | molecular_function | 1 | 442 | 9 | *Seita.5G058800* | | | | |
| GO:0098719 | sodium ion import across plasma membrane | biological_process | 1 | 442 | 9 | *Seita.4G138500* | | | | |
| GO:0030001 | metal ion transport | biological_process | 3 | 442 | 66 | *Seita.3G037000;Seita.8G028400;Seita.9G341900* | | | | |
| GO:0048046 | apoplast | cellular_component | 11 | 442 | 357 | *Seita.2G106500;Seita.2G313800;Seita.3G028500;Seita.4G003100;Seita.4G176600;Seita.4G246200;Seita.5G382800;Seita.8G212900;Seita.8G215100;Seita.9G005800;Seita.9G081900* | | | | |
| GO:0030163 | protein catabolic process | biological_process | 4 | 442 | 100 | *Seita.3G123800;Seita.3G183000;Seita.5G214300;Seita.9G461800* | | | | |
| GO:0010252 | auxin homeostasis | biological_process | 2 | 442 | 36 | *Seita.1G317400;Seita.3G124200* | | | | |
| GO:0031410 | cytoplasmic vesicle | cellular_component | 2 | 442 | 36 | *Seita.2G325500;Seita.8G049400* | | | | |
| GO:0006508 | proteolysis | biological_process | 4 | 442 | 101 | *Seita.3G123800;Seita.3G183000;Seita.5G214300;Seita.9G461800* | | | | |
| GO:0032541 | cortical endoplasmic reticulum | cellular_component | 2 | 442 | 37 | *Seita.1G292500;Seita.5G281400* | | | | |
| GO:0000036 | acyl carrier activity | molecular_function | 1 | 442 | 10 | *Seita.4G031400* | | | | |
| GO:0009247 | glycolipid biosynthetic process | biological_process | 1 | 442 | 10 | *Seita.6G110100* | | | | |
| GO:0009934 | regulation of meristem structural organization | biological_process | 1 | 442 | 10 | *Seita.4G070400* | | | | |
| GO:0016682 | oxidoreductase activity, acting on diphenols and related substances as donors, oxygen as acceptor | molecular_function | 1 | 442 | 10 | *Seita.5G444000* | | | | |
| GO:0033907 | beta-D-fucosidase activity | molecular_function | 1 | 442 | 10 | *Seita.2G415300* | | | | |
| GO:0035435 | phosphate ion transmembrane transport | biological_process | 1 | 442 | 10 | *Seita.1G216200* | | | | |
| GO:0044732 | mitotic spindle pole body | cellular_component | 1 | 442 | 10 | *Seita.2G335500* | | | | |
| GO:0045486 | naringenin 3-dioxygenase activity | molecular_function | 1 | 442 | 10 | *Seita.9G561500* | | | | |
| GO:0047259 | glucomannan 4-beta-mannosyltransferase activity | molecular_function | 1 | 442 | 10 | *Seita.1G054000* | | | | |
| GO:0047714 | galactolipase activity | molecular_function | 1 | 442 | 10 | *Seita.5G058800* | | | | |
| GO:0051502 | diterpene phytoalexin biosynthetic process | biological_process | 1 | 442 | 10 | *Seita.3G339800* | | | | |
| GO:1902456 | regulation of stomatal opening | biological_process | 1 | 442 | 10 | *Seita.9G234200* | | | | |
| GO:1902476 | chloride transmembrane transport | biological_process | 1 | 442 | 10 | *Seita.3G300700* | | | | |
| GO:2000762 | regulation of phenylpropanoid metabolic process | biological_process | 1 | 442 | 10 | *Seita.4G226700* | | | | |
| GO:0004497 | monooxygenase activity | molecular_function | 7 | 442 | 216 | *Seita.1G288100;Seita.2G094000;Seita.2G229900;Seita.2G404300;Seita.3G339800;Seita.8G027000;Seita.9G536500* | | | | |
| GO:0031347 | regulation of defense response | biological_process | 2 | 442 | 39 | *Seita.4G067200;Seita.4G097900* | | | | |
| GO:0045489 | pectin biosynthetic process | biological_process | 2 | 442 | 39 | *Seita.1G294300;Seita.5G303500* | | | | |
| GO:0002239 | response to oomycetes | biological_process | 1 | 442 | 11 | *Seita.9G536500* | | | | |
| GO:0004124 | cysteine synthase activity | molecular_function | 1 | 442 | 11 | *Seita.4G075200* | | | | |
| GO:0004338 | glucan exo-1,3-beta-glucosidase activity | molecular_function | 1 | 442 | 11 | *Seita.2G415300* | | | | |
| GO:0005786 | signal recognition particle, endoplasmic reticulum targeting | cellular_component | 1 | 442 | 11 | *Seita.9G552100* | | | | |
| GO:0006821 | chloride transport | biological_process | 1 | 442 | 11 | *Seita.3G300700* | | | | |
| GO:0009815 | 1-aminocyclopropane-1-carboxylate oxidase activity | molecular_function | 1 | 442 | 11 | *Seita.3G037900* | | | | |
| GO:0010099 | regulation of photomorphogenesis | biological_process | 1 | 442 | 11 | *Seita.1G043800* | | | | |
| GO:0016161 | beta-amylase activity | molecular_function | 1 | 442 | 11 | *Seita.9G229700* | | | | |
| GO:0016629 | 12-oxophytodienoate reductase activity | molecular_function | 1 | 442 | 11 | *Seita.5G169500* | | | | |
| GO:0045487 | gibberellin catabolic process | biological_process | 1 | 442 | 11 | *Seita.5G147400* | | | | |
| GO:0045543 | gibberellin 2-beta-dioxygenase activity | molecular_function | 1 | 442 | 11 | *Seita.5G147400* | | | | |
| GO:0048359 | mucilage metabolic process involved in seed coat development | biological_process | 1 | 442 | 11 | *Seita.9G081900* | | | | |
| GO:0090332 | stomatal closure | biological_process | 1 | 442 | 11 | *Seita.2G200700* | | | | |
| GO:0042973 | glucan endo-1,3-beta-D-glucosidase activity | molecular_function | 3 | 442 | 72 | *Seita.1G045800;Seita.3G225200;Seita.5G203400* | | | | |
| GO:0007275 | multicellular organism development | biological_process | 12 | 442 | 411 | *Seita.1G348600;Seita.2G094000;Seita.2G222900;Seita.2G295000;Seita.4G070400;Seita.4G266000;Seita.6G170000;Seita.6G170200;Seita.7G230900;Seita.8G027000;Seita.9G188100;Seita.9G436000* | | | | |
| GO:0000122 | negative regulation of transcription by RNA polymerase II | biological_process | 2 | 442 | 40 | *Seita.3G124200;Seita.8G123100* | | | | |
| GO:0009585 | red, far-red light phototransduction | biological_process | 2 | 442 | 41 | *Seita.1G043800;Seita.7G179600* | | | | |
| GO:0031418 | L-ascorbic acid binding | molecular_function | 2 | 442 | 41 | *Seita.3G037900;Seita.9G561500* | | | | |
| GO:0004564 | beta-fructofuranosidase activity | molecular_function | 1 | 442 | 12 | *Seita.3G028500* | | | | |
| GO:0005247 | voltage-gated chloride channel activity | molecular_function | 1 | 442 | 12 | *Seita.3G300700* | | | | |
| GO:0006012 | galactose metabolic process | biological_process | 1 | 442 | 12 | *Seita.2G282600* | | | | |
| GO:0006986 | response to unfolded protein | biological_process | 1 | 442 | 12 | *Seita.5G376100* | | | | |
| GO:0009862 | systemic acquired resistance, salicylic acid mediated signaling pathway | biological_process | 1 | 442 | 12 | *Seita.2G106500* | | | | |
| GO:0010439 | regulation of glucosinolate biosynthetic process | biological_process | 1 | 442 | 12 | *Seita.3G186600* | | | | |
| GO:0034707 | chloride channel complex | cellular_component | 1 | 442 | 12 | *Seita.3G300700* | | | | |
| GO:0042623 | ATPase activity, coupled | molecular_function | 1 | 442 | 12 | *Seita.5G376100* | | | | |
| GO:0043562 | cellular response to nitrogen levels | biological_process | 1 | 442 | 12 | *Seita.1G186400* | | | | |
| GO:0048262 | determination of dorsal/ventral asymmetry | biological_process | 1 | 442 | 12 | *Seita.3G225500* | | | | |
| GO:1905582 | response to mannose | biological_process | 1 | 442 | 12 | *Seita.1G276500* | | | | |
| GO:0102483 | scopolin beta-glucosidase activity | molecular_function | 2 | 442 | 42 | *Seita.2G415300;Seita.4G134400* | | | | |
| GO:0005887 | integral component of plasma membrane | cellular_component | 6 | 442 | 186 | *Seita.1G216200;Seita.6G193400;Seita.8G025400;Seita.9G436600;Seita.9G504800;Seita.9G505000* | | | | |
| GO:0071949 | FAD binding | molecular_function | 3 | 442 | 76 | *Seita.6G174400;Seita.9G217200;Seita.9G536500* | | | | |
| GO:0009644 | response to high light intensity | biological_process | 2 | 442 | 43 | *Seita.1G342400;Seita.9G552100* | | | | |
| GO:0000062 | fatty-acyl-CoA binding | molecular_function | 1 | 442 | 13 | *Seita.5G027400* | | | | |
| GO:0002213 | defense response to insect | biological_process | 1 | 442 | 13 | *Seita.5G058800* | | | | |
| GO:0008889 | glycerophosphodiester phosphodiesterase activity | molecular_function | 1 | 442 | 13 | *Seita.1G171900* | | | | |
| GO:0031176 | endo-1,4-beta-xylanase activity | molecular_function | 1 | 442 | 13 | *Seita.9G472200* | | | | |
| GO:0046898 | response to cycloheximide | biological_process | 1 | 442 | 13 | *Seita.1G239600* | | | | |
| GO:0071944 | cell periphery | cellular_component | 1 | 442 | 13 | *Seita.4G197200* | | | | |
| GO:0006970 | response to osmotic stress | biological_process | 7 | 442 | 228 | *Seita.1G326600;Seita.3G076200;Seita.3G160800;Seita.3G193800;Seita.5G284300;Seita.6G136200;Seita.9G440300* | | | | |
| GO:0022857 | transmembrane transporter activity | molecular_function | 4 | 442 | 116 | *Seita.3G068600;Seita.4G274900;Seita.5G351800;Seita.9G277500* | | | | |
| GO:0008422 | beta-glucosidase activity | molecular_function | 2 | 442 | 45 | *Seita.2G415300;Seita.4G134400* | | | | |
| GO:0048510 | regulation of timing of transition from vegetative to reproductive phase | biological_process | 2 | 442 | 45 | *Seita.5G394300;Seita.9G525000* | | | | |
| GO:0006098 | pentose-phosphate shunt | biological_process | 1 | 442 | 14 | *Seita.9G365500* | | | | |
| GO:0010183 | pollen tube guidance | biological_process | 1 | 442 | 14 | *Seita.9G079200* | | | | |
| GO:0010325 | raffinose family oligosaccharide biosynthetic process | biological_process | 1 | 442 | 14 | *Seita.6G195300* | | | | |
| GO:0015386 | potassium:proton antiporter activity | molecular_function | 1 | 442 | 14 | *Seita.4G138500* | | | | |
| GO:0030151 | molybdenum ion binding | molecular_function | 1 | 442 | 14 | *Seita.6G234900* | | | | |
| GO:0036297 | interstrand cross-link repair | biological_process | 1 | 442 | 14 | *Seita.3G046400* | | | | |
| GO:0048437 | floral organ development | biological_process | 1 | 442 | 14 | *Seita.3G048500* | | | | |
| GO:0055075 | potassium ion homeostasis | biological_process | 1 | 442 | 14 | *Seita.4G138500* | | | | |
| GO:0080001 | mucilage extrusion from seed coat | biological_process | 1 | 442 | 14 | *Seita.9G081900* | | | | |
| GO:0004601 | peroxidase activity | molecular_function | 6 | 442 | 194 | *Seita.1G022500;Seita.4G176600;Seita.6G210000;Seita.9G298200;Seita.9G562600;Seita.J003100* | | | | |
| GO:0009055 | electron transfer activity | molecular_function | 5 | 442 | 156 | *Seita.3G344000;Seita.5G137500;Seita.5G167100;Seita.6G070700;Seita.8G219700* | | | | |
| GO:2000022 | regulation of jasmonic acid mediated signaling pathway | biological_process | 2 | 442 | 46 | *Seita.2G140900;Seita.4G067200* | | | | |
| GO:0005506 | iron ion binding | molecular_function | 10 | 442 | 354 | *Seita.1G288100;Seita.2G094000;Seita.2G229900;Seita.2G404300;Seita.3G339800;Seita.6G023100;Seita.7G209000;Seita.7G323700;Seita.8G027000;Seita.9G469300* | | | | |
| GO:0009753 | response to jasmonic acid | biological_process | 4 | 442 | 119 | *Seita.2G140900;Seita.3G124200;Seita.5G027400;Seita.8G008100* | | | | |
| GO:0004518 | nuclease activity | molecular_function | 1 | 442 | 15 | *Seita.6G122900* | | | | |
| GO:0006535 | cysteine biosynthetic process from serine | biological_process | 1 | 442 | 15 | *Seita.4G075200* | | | | |
| GO:0006777 | Mo-molybdopterin cofactor biosynthetic process | biological_process | 1 | 442 | 15 | *Seita.6G234900* | | | | |
| GO:0010105 | negative regulation of ethylene-activated signaling pathway | biological_process | 1 | 442 | 15 | *Seita.7G232100* | | | | |
| GO:0010929 | positive regulation of auxin mediated signaling pathway | biological_process | 1 | 442 | 15 | *Seita.2G140900* | | | | |
| GO:0012501 | programmed cell death | biological_process | 1 | 442 | 15 | *Seita.9G461800* | | | | |
| GO:0016102 | diterpenoid biosynthetic process | biological_process | 1 | 442 | 15 | *Seita.3G339800* | | | | |
| GO:0046961 | proton-transporting ATPase activity, rotational mechanism | molecular_function | 1 | 442 | 15 | *Seita.5G017200* | | | | |
| GO:0050829 | defense response to Gram-negative bacterium | biological_process | 1 | 442 | 15 | *Seita.6G165800* | | | | |
| GO:0098754 | detoxification | biological_process | 1 | 442 | 15 | *Seita.2G275500* | | | | |
| GO:0000272 | polysaccharide catabolic process | biological_process | 2 | 442 | 48 | *Seita.1G225300;Seita.9G229700* | | | | |
| GO:0031146 | SCF-dependent proteasomal ubiquitin-dependent protein catabolic process | biological_process | 2 | 442 | 48 | *Seita.4G070400;Seita.9G512800* | | | | |
| GO:0004089 | carbonate dehydratase activity | molecular_function | 1 | 442 | 16 | *Seita.6G189200* | | | | |
| GO:0004857 | enzyme inhibitor activity | molecular_function | 1 | 442 | 16 | *Seita.9G196800* | | | | |
| GO:0006352 | DNA-templated transcription, initiation | biological_process | 1 | 442 | 16 | *Seita.3G124300* | | | | |
| GO:0009932 | cell tip growth | biological_process | 1 | 442 | 16 | *Seita.4G003100* | | | | |
| GO:0010629 | negative regulation of gene expression | biological_process | 1 | 442 | 16 | *Seita.1G236100* | | | | |
| GO:0015996 | chlorophyll catabolic process | biological_process | 1 | 442 | 16 | *Seita.2G134400* | | | | |
| GO:0045995 | regulation of embryonic development | biological_process | 1 | 442 | 16 | *Seita.8G008100* | | | | |
| GO:0048765 | root hair cell differentiation | biological_process | 1 | 442 | 16 | *Seita.2G236200* | | | | |
| GO:0004867 | serine-type endopeptidase inhibitor activity | molecular_function | 2 | 442 | 50 | *Seita.3G311700;Seita.9G030600* | | | | |
| GO:0005516 | calmodulin binding | molecular_function | 5 | 442 | 164 | *Seita.1G198500;Seita.4G100500;Seita.8G025400;Seita.8G128400;Seita.9G555000* | | | | |
| GO:0080167 | response to karrikin | biological_process | 3 | 442 | 87 | *Seita.3G123800;Seita.5G214300;Seita.7G090200* | | | | |
| GO:0005987 | sucrose catabolic process | biological_process | 1 | 442 | 17 | *Seita.5G157600* | | | | |
| GO:0009231 | riboflavin biosynthetic process | biological_process | 1 | 442 | 17 | *Seita.3G216400* | | | | |
| GO:0009410 | response to xenobiotic stimulus | biological_process | 1 | 442 | 17 | *Seita.1G117500* | | | | |
| GO:0009864 | induced systemic resistance, jasmonic acid mediated signaling pathway | biological_process | 1 | 442 | 17 | *Seita.7G232100* | | | | |
| GO:0010274 | hydrotropism | biological_process | 1 | 442 | 17 | *Seita.1G292500* | | | | |
| GO:0016597 | amino acid binding | molecular_function | 1 | 442 | 17 | *Seita.7G076400* | | | | |
| GO:0071577 | zinc ion transmembrane transport | biological_process | 1 | 442 | 17 | *Seita.2G090600* | | | | |
| GO:0015293 | symporter activity | molecular_function | 3 | 442 | 89 | *Seita.1G216200;Seita.7G032900;Seita.9G436600* | | | | |
| GO:0005975 | carbohydrate metabolic process | biological_process | 8 | 442 | 290 | *Seita.1G045800;Seita.2G415300;Seita.3G028500;Seita.3G196200;Seita.3G225200;Seita.3G263800;Seita.4G134400;Seita.5G203400* | | | | |
| GO:0002183 | cytoplasmic translational initiation | biological_process | 1 | 442 | 18 | *Seita.3G147500* | | | | |
| GO:0016604 | nuclear body | cellular_component | 1 | 442 | 18 | *Seita.7G232100* | | | | |
| GO:0034620 | cellular response to unfolded protein | biological_process | 1 | 442 | 18 | *Seita.5G376100* | | | | |
| GO:0010311 | lateral root formation | biological_process | 2 | 442 | 54 | *Seita.9G504800;Seita.9G505000* | | | | |
| GO:0046274 | lignin catabolic process | biological_process | 2 | 442 | 54 | *Seita.5G382800;Seita.8G212900* | | | | |
| GO:0052716 | hydroquinone:oxygen oxidoreductase activity | molecular_function | 2 | 442 | 54 | *Seita.5G382800;Seita.8G212900* | | | | |
| GO:0006207 | 'de novo' pyrimidine nucleobase biosynthetic process | biological_process | 1 | 442 | 19 | *Seita.3G138700* | | | | |
| GO:0008324 | cation transmembrane transporter activity | molecular_function | 1 | 442 | 19 | *Seita.9G478600* | | | | |
| GO:0051289 | protein homotetramerization | biological_process | 1 | 442 | 19 | *Seita.1G117500* | | | | |
| GO:0061077 | chaperone-mediated protein folding | biological_process | 1 | 442 | 19 | *Seita.5G065300* | | | | |
| GO:0070300 | phosphatidic acid binding | molecular_function | 1 | 442 | 19 | *Seita.9G417000* | | | | |
| GO:0043531 | ADP binding | molecular_function | 10 | 442 | 379 | *Seita.2G056400;Seita.2G057000;Seita.3G076300;Seita.7G131700;Seita.7G241600;Seita.8G242600;Seita.8G242800;Seita.8G243800;Seita.8G244000;Seita.9G198200* | | | | |
| GO:0003680 | AT DNA binding | molecular_function | 1 | 442 | 20 | *Seita.6G185100* | | | | |
| GO:0004806 | triglyceride lipase activity | molecular_function | 1 | 442 | 20 | *Seita.5G327000* | | | | |
| GO:0005385 | zinc ion transmembrane transporter activity | molecular_function | 1 | 442 | 20 | *Seita.2G090600* | | | | |
| GO:0005388 | calcium-transporting ATPase activity | molecular_function | 1 | 442 | 20 | *Seita.8G025400* | | | | |
| GO:0006006 | glucose metabolic process | biological_process | 1 | 442 | 20 | *Seita.9G365500* | | | | |
| GO:0015743 | malate transport | biological_process | 1 | 442 | 20 | *Seita.2G251700* | | | | |
| GO:0016759 | cellulose synthase activity | molecular_function | 1 | 442 | 20 | *Seita.1G268900* | | | | |
| GO:0030234 | enzyme regulator activity | molecular_function | 1 | 442 | 20 | *Seita.3G196600* | | | | |
| GO:0070647 | protein modification by small protein conjugation or removal | biological_process | 1 | 442 | 20 | *Seita.2G155300* | | | | |
| GO:0071805 | potassium ion transmembrane transport | biological_process | 1 | 442 | 20 | *Seita.4G138500* | | | | |
| GO:0009740 | gibberellic acid mediated signaling pathway | biological_process | 2 | 442 | 57 | *Seita.2G222900;Seita.3G370000* | | | | |
| GO:0016758 | transferase activity, transferring hexosyl groups | molecular_function | 2 | 442 | 57 | *Seita.2G275500;Seita.3G190000* | | | | |
| GO:0016567 | protein ubiquitination | biological_process | 17 | 442 | 680 | *Seita.1G327300;Seita.2G238400;Seita.2G401300;Seita.3G007200;Seita.3G088000;Seita.3G227200;Seita.4G042500;Seita.4G070400;Seita.6G136200;Seita.6G186400;Seita.8G016000;Seita.9G234200;Seita.9G349000;Seita.9G373200;Seita.9G497900;Seita.9G512800;Seita.9G567400* | | | | |
| GO:0031966 | mitochondrial membrane | cellular_component | 2 | 442 | 58 | *Seita.6G235000;Seita.9G050000* | | | | |
| GO:0009505 | plant-type cell wall | cellular_component | 7 | 442 | 261 | *Seita.3G123800;Seita.3G183000;Seita.4G003100;Seita.5G203400;Seita.5G214300;Seita.9G081900;Seita.J003100* | | | | |
| GO:0046982 | protein heterodimerization activity | molecular_function | 4 | 442 | 138 | *Seita.3G368200;Seita.6G045900;Seita.9G112800;Seita.9G170900* | | | | |
| GO:0006520 | cellular amino acid metabolic process | biological_process | 1 | 442 | 21 | *Seita.9G216200* | | | | |
| GO:0010167 | response to nitrate | biological_process | 1 | 442 | 21 | *Seita.3G300700* | | | | |
| GO:0015112 | nitrate transmembrane transporter activity | molecular_function | 1 | 442 | 21 | *Seita.3G300700* | | | | |
| GO:0090351 | seedling development | biological_process | 1 | 442 | 21 | *Seita.3G076200* | | | | |
| GO:0016491 | oxidoreductase activity | molecular_function | 6 | 442 | 223 | *Seita.1G349400;Seita.3G020400;Seita.3G020600;Seita.3G297700;Seita.7G209000;Seita.9G436000* | | | | |
| GO:0009845 | seed germination | biological_process | 2 | 442 | 60 | *Seita.3G076200;Seita.6G210000* | | | | |
| GO:0004707 | MAP kinase activity | molecular_function | 1 | 442 | 22 | *Seita.3G145200* | | | | |
| GO:0048653 | anther development | biological_process | 1 | 442 | 22 | *Seita.3G164400* | | | | |
| GO:1990578 | perinuclear endoplasmic reticulum membrane | cellular_component | 1 | 442 | 22 | *Seita.5G281400* | | | | |
| GO:0016740 | transferase activity | molecular_function | 5 | 442 | 183 | *Seita.1G349700;Seita.3G227200;Seita.4G042500;Seita.9G234200;Seita.9G373200* | | | | |
| GO:0051213 | dioxygenase activity | molecular_function | 3 | 442 | 101 | *Seita.4G015600;Seita.5G147400;Seita.5G444000* | | | | |
| GO:0000155 | phosphorelay sensor kinase activity | molecular_function | 1 | 442 | 23 | *Seita.5G132900* | | | | |
| GO:0000976 | transcription regulatory region sequence-specific DNA binding | molecular_function | 1 | 442 | 23 | *Seita.3G124200* | | | | |
| GO:0006413 | translational initiation | biological_process | 1 | 442 | 23 | *Seita.3G147500* | | | | |
| GO:0006814 | sodium ion transport | biological_process | 1 | 442 | 23 | *Seita.2G251700* | | | | |
| GO:0009693 | ethylene biosynthetic process | biological_process | 1 | 442 | 23 | *Seita.3G037900* | | | | |
| GO:0010193 | response to ozone | biological_process | 1 | 442 | 23 | *Seita.4G067200* | | | | |
| GO:0048366 | leaf development | biological_process | 2 | 442 | 62 | *Seita.3G124200;Seita.9G355300* | | | | |
| GO:0046983 | protein dimerization activity | molecular_function | 8 | 442 | 312 | *Seita.2G228700;Seita.2G263000;Seita.3G055700;Seita.5G204500;Seita.5G336800;Seita.7G149900;Seita.9G067200;Seita.9G511700* | | | | |
| GO:0046658 | anchored component of plasma membrane | cellular_component | 5 | 442 | 186 | *Seita.1G045800;Seita.3G225200;Seita.4G003100;Seita.5G203400;Seita.6G070700* | | | | |
| GO:0010150 | leaf senescence | biological_process | 4 | 442 | 145 | *Seita.3G124200;Seita.4G067200;Seita.5G233100;Seita.5G444000* | | | | |
| GO:0005623 | cell | cellular_component | 1 | 442 | 24 | *Seita.2G436600* | | | | |
| GO:0005938 | cell cortex | cellular_component | 1 | 442 | 24 | *Seita.4G197200* | | | | |
| GO:0008283 | cell proliferation | biological_process | 1 | 442 | 24 | *Seita.4G097900* | | | | |
| GO:0009556 | microsporogenesis | biological_process | 1 | 442 | 24 | *Seita.3G048500* | | | | |
| GO:0009639 | response to red or far red light | biological_process | 1 | 442 | 24 | *Seita.7G179600* | | | | |
| GO:0015991 | ATP hydrolysis coupled proton transport | biological_process | 1 | 442 | 24 | *Seita.5G017200* | | | | |
| GO:0042026 | protein refolding | biological_process | 1 | 442 | 24 | *Seita.5G376100* | | | | |
| GO:0042594 | response to starvation | biological_process | 1 | 442 | 24 | *Seita.1G276500* | | | | |
| GO:0055072 | iron ion homeostasis | biological_process | 1 | 442 | 24 | *Seita.9G355300* | | | | |
| GO:2000070 | regulation of response to water deprivation | biological_process | 1 | 442 | 24 | *Seita.1G248300* | | | | |
| GO:0042742 | defense response to bacterium | biological_process | 8 | 442 | 317 | *Seita.2G134400;Seita.2G140900;Seita.3G047600;Seita.3G048500;Seita.4G067200;Seita.5G203200;Seita.7G131700;Seita.9G497900* | | | | |
| GO:0009408 | response to heat | biological_process | 4 | 442 | 148 | *Seita.1G326600;Seita.1G342400;Seita.4G227700;Seita.5G092600* | | | | |
| GO:0009835 | fruit ripening | biological_process | 1 | 442 | 25 | *Seita.3G037900* | | | | |
| GO:0009863 | salicylic acid mediated signaling pathway | biological_process | 1 | 442 | 25 | *Seita.2G434600* | | | | |
| GO:0010154 | fruit development | biological_process | 1 | 442 | 25 | *Seita.9G050000* | | | | |
| GO:0030544 | Hsp70 protein binding | molecular_function | 1 | 442 | 25 | *Seita.3G147500* | | | | |
| GO:0034765 | regulation of ion transmembrane transport | biological_process | 1 | 442 | 25 | *Seita.3G300700* | | | | |
| GO:0044183 | protein binding involved in protein folding | molecular_function | 1 | 442 | 25 | *Seita.5G376100* | | | | |
| GO:0050734 | hydroxycinnamoyltransferase activity | molecular_function | 1 | 442 | 25 | *Seita.2G293600* | | | | |
| GO:0004565 | beta-galactosidase activity | molecular_function | 1 | 442 | 26 | *Seita.2G415300* | | | | |
| GO:0006541 | glutamine metabolic process | biological_process | 1 | 442 | 26 | *Seita.3G138700* | | | | |
| GO:0008970 | phospholipase A1 activity | molecular_function | 1 | 442 | 26 | *Seita.5G058800* | | | | |
| GO:0009881 | photoreceptor activity | molecular_function | 1 | 442 | 26 | *Seita.5G132900* | | | | |
| GO:0010229 | inflorescence development | biological_process | 1 | 442 | 26 | *Seita.4G097900* | | | | |
| GO:0009739 | response to gibberellin | biological_process | 3 | 442 | 109 | *Seita.2G140900;Seita.3G124200;Seita.4G097900* | | | | |
| GO:0005737 | cytoplasm | cellular_component | 56 | 442 | 2424 | *Seita.1G001900;Seita.1G045800;Seita.1G067400;Seita.1G239600;Seita.1G248300;Seita.1G292500;Seita.1G310800;Seita.1G315700;Seita.1G342400;Seita.1G347300;Seita.2G197800;Seita.2G295000;Seita.2G401300;Seita.3G053800;Seita.3G076200;Seita.3G138700;Seita.3G145200;Seita.3G160800;Seita.3G205500;Seita.3G225200;Seita.3G344000;Seita.3G373500;Seita.3G380200;Seita.4G105600;Seita.4G188900;Seita.4G197200;Seita.4G227700;Seita.4G266000;Seita.5G027400;Seita.5G092600;Seita.5G137500;Seita.5G167100;Seita.5G284300;Seita.5G318400;Seita.5G319300;Seita.5G363900;Seita.5G376100;Seita.6G045900;Seita.6G144600;Seita.6G170000;Seita.6G170200;Seita.6G200300;Seita.6G235000;Seita.7G140200;Seita.7G202900;Seita.7G230900;Seita.8G219700;Seita.9G042400;Seita.9G051600;Seita.9G064500;Seita.9G253000;Seita.9G369800;Seita.9G437300;Seita.9G440300;Seita.9G461500;Seita.9G500200* | | | | |
| GO:0001228 | DNA-binding transcription activator activity, RNA polymerase II-specific | molecular_function | 1 | 442 | 27 | *Seita.7G149900* | | | | |
| GO:0003713 | transcription coactivator activity | molecular_function | 1 | 442 | 27 | *Seita.4G227700* | | | | |
| GO:0006662 | glycerol ether metabolic process | biological_process | 1 | 442 | 27 | *Seita.9G051600* | | | | |
| GO:0009646 | response to absence of light | biological_process | 1 | 442 | 27 | *Seita.3G124200* | | | | |
| GO:0015250 | water channel activity | molecular_function | 1 | 442 | 27 | *Seita.2G291500* | | | | |
| GO:0016117 | carotenoid biosynthetic process | biological_process | 1 | 442 | 27 | *Seita.7G209000* | | | | |
| GO:0046854 | phosphatidylinositol phosphorylation | biological_process | 1 | 442 | 27 | *Seita.2G200700* | | | | |
| GO:0047262 | polygalacturonate 4-alpha-galacturonosyltransferase activity | molecular_function | 1 | 442 | 27 | *Seita.5G303500* | | | | |
| GO:0008194 | UDP-glycosyltransferase activity | molecular_function | 3 | 442 | 111 | *Seita.2G275500;Seita.9G086500;Seita.9G239700* | | | | |
| GO:0016709 | oxidoreductase activity,acting on paired donors, with incorporation or reduction of molecular oxygen, NAD(P)H as one donor, and incorporation of one atom of oxygen | molecular_function | 3 | 442 | 111 | *Seita.3G339800;Seita.6G023100;Seita.9G469300* | | | | |
| GO:0061630 | ubiquitin protein ligase activity | molecular_function | 5 | 442 | 197 | *Seita.2G155300;Seita.2G401300;Seita.3G088000;Seita.9G238100;Seita.9G311400* | | | | |
| GO:0016887 | ATPase activity | molecular_function | 5 | 442 | 199 | *Seita.2G213800;Seita.5G376100;Seita.8G215500;Seita.9G050000;Seita.9G190200* | | | | |
| GO:0009751 | response to salicylic acid | biological_process | 4 | 442 | 156 | *Seita.2G140900;Seita.3G124200;Seita.4G067200;Seita.7G179600* | | | | |
| GO:0009695 | jasmonic acid biosynthetic process | biological_process | 1 | 442 | 28 | *Seita.5G169500* | | | | |
| GO:0010218 | response to far red light | biological_process | 1 | 442 | 28 | *Seita.1G043800* | | | | |
| GO:0046621 | negative regulation of organ growth | biological_process | 1 | 442 | 28 | *Seita.2G295000* | | | | |
| GO:0009501 | amyloplast | cellular_component | 1 | 442 | 29 | *Seita.2G229000* | | | | |
| GO:0016705 | oxidoreductase activity, acting on paired donors, with incorporation or reduction of molecular oxygen | molecular_function | 4 | 442 | 159 | *Seita.1G288100;Seita.2G094000;Seita.2G404300;Seita.8G027000* | | | | |
| GO:0009744 | response to sucrose | biological_process | 2 | 442 | 72 | *Seita.1G276500;Seita.3G124200* | | | | |
| GO:0009860 | pollen tube growth | biological_process | 2 | 442 | 73 | *Seita.1G349700;Seita.2G410400* | | | | |
| GO:0010468 | regulation of gene expression | biological_process | 2 | 442 | 73 | *Seita.2G228700;Seita.3G145200* | | | | |
| GO:0009723 | response to ethylene | biological_process | 4 | 442 | 161 | *Seita.2G140900;Seita.3G124200;Seita.5G027400;Seita.7G232100* | | | | |
| GO:0004675 | transmembrane receptor protein serine/threonine kinase activity | molecular_function | 1 | 442 | 30 | *Seita.3G047600* | | | | |
| GO:0010315 | auxin efflux | biological_process | 1 | 442 | 30 | *Seita.1G317400* | | | | |
| GO:0005351 | carbohydrate:proton symporter activity | molecular_function | 2 | 442 | 74 | *Seita.9G504800;Seita.9G505000* | | | | |
| GO:0005355 | glucose transmembrane transporter activity | molecular_function | 2 | 442 | 74 | *Seita.9G504800;Seita.9G505000* | | | | |
| GO:0008645 | hexose transmembrane transport | biological_process | 2 | 442 | 74 | *Seita.9G504800;Seita.9G505000* | | | | |
| GO:0009833 | plant-type primary cell wall biogenesis | biological_process | 1 | 442 | 31 | *Seita.1G268900* | | | | |
| GO:0010223 | secondary shoot formation | biological_process | 1 | 442 | 31 | *Seita.9G500200* | | | | |
| GO:0042335 | cuticle development | biological_process | 1 | 442 | 31 | *Seita.4G070400* | | | | |
| GO:0042538 | hyperosmotic salinity response | biological_process | 1 | 442 | 31 | *Seita.9G427100* | | | | |
| GO:0003333 | amino acid transmembrane transport | biological_process | 2 | 442 | 75 | *Seita.1G054800;Seita.1G268000* | | | | |
| GO:0006869 | lipid transport | biological_process | 2 | 442 | 75 | *Seita.2G106500;Seita.5G027400* | | | | |
| GO:1900150 | regulation of defense response to fungus | biological_process | 2 | 442 | 76 | *Seita.2G140900;Seita.2G434600* | | | | |
| GO:0004568 | chitinase activity | molecular_function | 1 | 442 | 32 | *Seita.1G225300* | | | | |
| GO:0031977 | thylakoid lumen | cellular_component | 1 | 442 | 32 | *Seita.5G099500* | | | | |
| GO:0034599 | cellular response to oxidative stress | biological_process | 1 | 442 | 32 | *Seita.6G210000* | | | | |
| GO:0052689 | carboxylic ester hydrolase activity | molecular_function | 2 | 442 | 77 | *Seita.2G410400;Seita.5G109600* | | | | |
| GO:0009611 | response to wounding | biological_process | 4 | 442 | 168 | *Seita.2G289600;Seita.3G311700;Seita.6G159400;Seita.7G323700* | | | | |
| GO:0009631 | cold acclimation | biological_process | 1 | 442 | 33 | *Seita.1G117500* | | | | |
| GO:0016760 | cellulose synthase (UDP-forming) activity | molecular_function | 1 | 442 | 33 | *Seita.1G268900* | | | | |
| GO:0030659 | cytoplasmic vesicle membrane | cellular_component | 1 | 442 | 33 | *Seita.2G094000* | | | | |
| GO:0009627 | systemic acquired resistance | biological_process | 1 | 442 | 34 | *Seita.2G106500* | | | | |
| GO:0010114 | response to red light | biological_process | 1 | 442 | 34 | *Seita.1G043800* | | | | |
| GO:0010228 | vegetative to reproductive phase transition of meristem | biological_process | 2 | 442 | 80 | *Seita.3G193800;Seita.9G525000* | | | | |
| GO:0015171 | amino acid transmembrane transporter activity | molecular_function | 2 | 442 | 81 | *Seita.1G054800;Seita.1G268000* | | | | |
| GO:0051539 | 4 iron, 4 sulfur cluster binding | molecular_function | 2 | 442 | 81 | *Seita.2G327500;Seita.3G046400* | | | | |
| GO:0080043 | quercetin 3-O-glucosyltransferase activity | molecular_function | 2 | 442 | 81 | *Seita.9G086500;Seita.9G239700* | | | | |
| GO:0080044 | quercetin 7-O-glucosyltransferase activity | molecular_function | 2 | 442 | 81 | *Seita.9G086500;Seita.9G239700* | | | | |
| GO:0006032 | chitin catabolic process | biological_process | 1 | 442 | 35 | *Seita.1G225300* | | | | |
| GO:0010329 | auxin efflux transmembrane transporter activity | molecular_function | 1 | 442 | 35 | *Seita.1G317400* | | | | |
| GO:0045493 | xylan catabolic process | biological_process | 1 | 442 | 35 | *Seita.9G472200* | | | | |
| GO:0009749 | response to glucose | biological_process | 1 | 442 | 37 | *Seita.1G276500* | | | | |
| GO:0031072 | heat shock protein binding | molecular_function | 1 | 442 | 37 | *Seita.5G376100* | | | | |
| GO:2000031 | regulation of salicylic acid mediated signaling pathway | biological_process | 1 | 442 | 37 | *Seita.2G140900* | | | | |
| GO:0009570 | chloroplast stroma | cellular_component | 10 | 442 | 452 | *Seita.1G288400;Seita.1G339600;Seita.2G035400;Seita.4G075200;Seita.4G281200;Seita.5G058800;Seita.5G067800;Seita.6G189200;Seita.9G004600;Seita.9G156500* | | | | |
| GO:0043161 | proteasome-mediated ubiquitin-dependent protein catabolic process | biological_process | 2 | 442 | 85 | *Seita.2G155300;Seita.5G287300* | | | | |
| GO:0010087 | phloem or xylem histogenesis | biological_process | 1 | 442 | 38 | *Seita.5G034500* | | | | |
| GO:0005576 | extracellular region | cellular_component | 21 | 442 | 964 | *Seita.1G022500;Seita.1G045800;Seita.1G345000;Seita.2G038200;Seita.2G050800;Seita.2G289600;Seita.3G075300;Seita.3G105100;Seita.3G225200;Seita.3G330700;Seita.4G040400;Seita.5G203400;Seita.5G260400;Seita.6G014300;Seita.6G159400;Seita.8G056100;Seita.9G030600;Seita.9G081900;Seita.9G298200;Seita.9G562600;Seita.J003100* | | | | |
| GO:0019722 | calcium-mediated signaling | biological_process | 1 | 442 | 41 | *Seita.3G196600* | | | | |
| GO:0031408 | oxylipin biosynthetic process | biological_process | 1 | 442 | 41 | *Seita.5G169500* | | | | |
| GO:1901002 | positive regulation of response to salt stress | biological_process | 1 | 442 | 41 | *Seita.9G039200* | | | | |
| GO:0035673 | oligopeptide transmembrane transporter activity | molecular_function | 2 | 442 | 91 | *Seita.3G093400;Seita.6G193400* | | | | |
| GO:0000209 | protein polyubiquitination | biological_process | 1 | 442 | 42 | *Seita.2G401300* | | | | |
| GO:0004683 | calmodulin-dependent protein kinase activity | molecular_function | 1 | 442 | 42 | *Seita.1G239600* | | | | |
| GO:0009931 | calcium-dependent protein serine/threonine kinase activity | molecular_function | 1 | 442 | 42 | *Seita.1G239600* | | | | |
| GO:0032482 | Rab protein signal transduction | biological_process | 1 | 442 | 42 | *Seita.2G335500* | | | | |
| GO:0009908 | flower development | biological_process | 5 | 442 | 236 | *Seita.3G225500;Seita.3G370000;Seita.6G045900;Seita.9G298200;Seita.9G525000* | | | | |
| GO:0030170 | pyridoxal phosphate binding | molecular_function | 2 | 442 | 93 | *Seita.6G234900;Seita.9G216200* | | | | |
| GO:0010181 | FMN binding | molecular_function | 1 | 442 | 43 | *Seita.5G169500* | | | | |
| GO:0009909 | regulation of flower development | biological_process | 2 | 442 | 95 | *Seita.1G067400;Seita.4G188900* | | | | |
| GO:0000160 | phosphorelay signal transduction system | biological_process | 1 | 442 | 45 | *Seita.1G236100* | | | | |
| GO:0030244 | cellulose biosynthetic process | biological_process | 1 | 442 | 45 | *Seita.1G268900* | | | | |
| GO:0030154 | cell differentiation | biological_process | 4 | 442 | 194 | *Seita.5G025200;Seita.5G034500;Seita.7G179600;Seita.9G432200* | | | | |
| GO:0006979 | response to oxidative stress | biological_process | 6 | 442 | 291 | *Seita.1G022500;Seita.1G117500;Seita.4G176600;Seita.9G298200;Seita.9G562600;Seita.J003100* | | | | |
| GO:0043231 | intracellular membrane-bounded organelle | cellular_component | 3 | 442 | 148 | *Seita.7G323700;Seita.9G086500;Seita.9G239700* | | | | |
| GO:0010119 | regulation of stomatal movement | biological_process | 1 | 442 | 47 | *Seita.5G034500* | | | | |
| GO:0005215 | transporter activity | molecular_function | 1 | 442 | 48 | *Seita.1G317400* | | | | |
| GO:0016791 | phosphatase activity | molecular_function | 1 | 442 | 48 | *Seita.8G074800* | | | | |
| GO:2000012 | regulation of auxin polar transport | biological_process | 1 | 442 | 48 | *Seita.2G295000* | | | | |
| GO:0000079 | regulation of cyclin-dependent protein serine/threonine kinase activity | biological_process | 1 | 442 | 49 | *Seita.3G251400* | | | | |
| GO:0009543 | chloroplast thylakoid lumen | cellular_component | 1 | 442 | 49 | *Seita.5G099500* | | | | |
| GO:0042128 | nitrate assimilation | biological_process | 1 | 442 | 49 | *Seita.3G196200* | | | | |
| GO:0016042 | lipid catabolic process | biological_process | 5 | 442 | 251 | *Seita.3G048500;Seita.3G075300;Seita.3G340200;Seita.3G386800;Seita.5G058800* | | | | |
| GO:0019900 | kinase binding | molecular_function | 1 | 442 | 51 | *Seita.1G276500* | | | | |
| GO:0043621 | protein self-association | molecular_function | 3 | 442 | 158 | *Seita.1G342400;Seita.2G106500;Seita.5G092600* | | | | |
| GO:0000166 | nucleotide binding | molecular_function | 1 | 442 | 53 | *Seita.2G275500* | | | | |
| GO:0009789 | positive regulation of abscisic acid-activated signaling pathway | biological_process | 1 | 442 | 53 | *Seita.9G238100* | | | | |
| GO:0015706 | nitrate transport | biological_process | 1 | 442 | 53 | *Seita.3G300700* | | | | |
| GO:0030433 | ubiquitin-dependent ERAD pathway | biological_process | 1 | 442 | 54 | *Seita.2G155300* | | | | |
| GO:0009735 | response to cytokinin | biological_process | 2 | 442 | 110 | *Seita.4G015600;Seita.4G097900* | | | | |
| GO:1900426 | positive regulation of defense response to bacterium | biological_process | 1 | 442 | 55 | *Seita.1G246000* | | | | |
| GO:0055085 | transmembrane transport | biological_process | 2 | 442 | 111 | *Seita.1G271500;Seita.2G302500* | | | | |
| GO:0009409 | response to cold | biological_process | 5 | 442 | 265 | *Seita.1G262000;Seita.1G362000;Seita.3G242400;Seita.4G225400;Seita.9G050000* | | | | |
| GO:0009630 | gravitropism | biological_process | 1 | 442 | 56 | *Seita.3G088000* | | | | |
| GO:0051537 | 2 iron, 2 sulfur cluster binding | molecular_function | 1 | 442 | 56 | *Seita.5G137500* | | | | |
| GO:0005622 | intracellular | cellular_component | 2 | 442 | 114 | *Seita.3G048500;Seita.3G300700* | | | | |
| GO:0005874 | microtubule | cellular_component | 3 | 442 | 168 | *Seita.1G007800;Seita.2G352400;Seita.8G215500* | | | | |
| GO:0004721 | phosphoprotein phosphatase activity | molecular_function | 1 | 442 | 59 | *Seita.8G074800* | | | | |
| GO:0016747 | transferase activity, transferring acyl groups other than amino-acyl groups | molecular_function | 1 | 442 | 59 | *Seita.5G126800* | | | | |
| GO:0009553 | embryo sac development | biological_process | 1 | 442 | 60 | *Seita.3G048500* | | | | |
| GO:0005871 | kinesin complex | cellular_component | 1 | 442 | 61 | *Seita.8G215500* | | | | |
| GO:0018298 | protein-chromophore linkage | biological_process | 1 | 442 | 61 | *Seita.5G132900* | | | | |
| GO:0003777 | microtubule motor activity | molecular_function | 1 | 442 | 62 | *Seita.8G215500* | | | | |
| GO:0007018 | microtubule-based movement | biological_process | 1 | 442 | 62 | *Seita.8G215500* | | | | |
| GO:0045292 | mRNA cis splicing, via spliceosome | biological_process | 1 | 442 | 62 | *Seita.1G239000* | | | | |
| GO:0005507 | copper ion binding | molecular_function | 3 | 442 | 175 | *Seita.4G003100;Seita.5G382800;Seita.8G212900* | | | | |
| GO:0005743 | mitochondrial inner membrane | cellular_component | 3 | 442 | 175 | *Seita.3G164400;Seita.9G254900;Seita.9G520900* | | | | |
| GO:0008168 | methyltransferase activity | molecular_function | 1 | 442 | 64 | *Seita.8G122400* | | | | |
| GO:0018105 | peptidyl-serine phosphorylation | biological_process | 1 | 442 | 64 | *Seita.1G239600* | | | | |
| GO:0042752 | regulation of circadian rhythm | biological_process | 1 | 442 | 64 | *Seita.1G376200* | | | | |
| GO:0030145 | manganese ion binding | molecular_function | 1 | 442 | 65 | *Seita.8G074800* | | | | |
| GO:0006855 | drug transmembrane transport | biological_process | 1 | 442 | 66 | *Seita.2G001400* | | | | |
| GO:1990904 | ribonucleoprotein complex | cellular_component | 1 | 442 | 66 | *Seita.1G239000* | | | | |
| GO:0007049 | cell cycle | biological_process | 2 | 442 | 126 | *Seita.1G348600;Seita.3G251400* | | | | |
| GO:0004672 | protein kinase activity | molecular_function | 2 | 442 | 127 | *Seita.5G203200;Seita.8G152800* | | | | |
| GO:0005509 | calcium ion binding | molecular_function | 4 | 442 | 237 | *Seita.3G196600;Seita.3G261400;Seita.9G427100;Seita.9G456100* | | | | |
| GO:0006468 | protein phosphorylation | biological_process | 5 | 442 | 290 | *Seita.3G053800;Seita.3G083300;Seita.3G380200;Seita.4G232700;Seita.5G203200* | | | | |
| GO:0000786 | nucleosome | cellular_component | 1 | 442 | 68 | *Seita.9G112800* | | | | |
| GO:0015238 | drug transmembrane transporter activity | molecular_function | 1 | 442 | 68 | *Seita.2G001400* | | | | |
| GO:0008289 | lipid binding | molecular_function | 2 | 442 | 129 | *Seita.5G027400;Seita.7G105000* | | | | |
| GO:0032580 | Golgi cisterna membrane | cellular_component | 1 | 442 | 69 | *Seita.9G542400* | | | | |
| GO:0009536 | plastid | cellular_component | 3 | 442 | 186 | *Seita.1G171900;Seita.4G031400;Seita.4G281200* | | | | |
| GO:0006511 | ubiquitin-dependent protein catabolic process | biological_process | 4 | 442 | 241 | *Seita.2G155300;Seita.4G070400;Seita.9G238100;Seita.9G311400* | | | | |
| GO:0009620 | response to fungus | biological_process | 1 | 442 | 70 | *Seita.2G434600* | | | | |
| GO:0009736 | cytokinin-activated signaling pathway | biological_process | 1 | 442 | 70 | *Seita.2G222900* | | | | |
| GO:0009809 | lignin biosynthetic process | biological_process | 1 | 442 | 70 | *Seita.4G176600* | | | | |
| GO:0045087 | innate immune response | biological_process | 1 | 442 | 70 | *Seita.8G152800* | | | | |
| GO:0009706 | chloroplast inner membrane | cellular_component | 1 | 442 | 71 | *Seita.1G216200* | | | | |
| GO:0019901 | protein kinase binding | molecular_function | 1 | 442 | 71 | *Seita.3G251400* | | | | |
| GO:0003899 | DNA-directed 5'-3' RNA polymerase activity | molecular_function | 1 | 442 | 72 | *Seita.3G124300* | | | | |
| GO:0009941 | chloroplast envelope | cellular_component | 5 | 442 | 298 | *Seita.1G216200;Seita.5G067800;Seita.9G254900;Seita.9G520900;Seita.9G552100* | | | | |
| GO:0016020 | membrane | cellular_component | 16 | 442 | 850 | *Seita.1G182300;Seita.1G186400;Seita.1G292500;Seita.1G308800;Seita.1G317400;Seita.1G362000;Seita.3G123800;Seita.3G339800;Seita.4G003100;Seita.4G116600;Seita.5G214300;Seita.6G023100;Seita.6G165800;Seita.6G193400;Seita.7G105000;Seita.9G469300* | | | | |
| GO:0046872 | metal ion binding | molecular_function | 51 | 442 | 2504 | *Seita.1G022500;Seita.1G117500;Seita.1G186400;Seita.1G203100;Seita.1G276500;Seita.1G288400;Seita.1G310800;Seita.1G327300;Seita.2G035400;Seita.2G177500;Seita.2G222900;Seita.2G238400;Seita.2G327500;Seita.2G401300;Seita.3G037000;Seita.3G037900;Seita.3G046400;Seita.3G088000;Seita.3G139000;Seita.3G216400;Seita.3G218800;Seita.3G227200;Seita.3G368200;Seita.3G382100;Seita.4G015600;Seita.4G042500;Seita.4G176600;Seita.5G137500;Seita.5G147400;Seita.5G379400;Seita.5G444000;Seita.6G070700;Seita.6G161100;Seita.6G186400;Seita.6G198600;Seita.6G210000;Seita.8G025400;Seita.8G028400;Seita.9G156500;Seita.9G170900;Seita.9G238100;Seita.9G298200;Seita.9G311400;Seita.9G341900;Seita.9G373200;Seita.9G440500;Seita.9G474600;Seita.9G525000;Seita.9G561500;Seita.9G562600;Seita.J003100* | | | | |
| GO:0005615 | extracellular space | cellular_component | 2 | 442 | 136 | *Seita.6G174400;Seita.9G217200* | | | | |
| GO:0040008 | regulation of growth | biological_process | 3 | 442 | 194 | *Seita.2G263000;Seita.4G266000;Seita.7G230900* | | | | |
| GO:0042803 | protein homodimerization activity | molecular_function | 3 | 442 | 195 | *Seita.8G152800;Seita.9G039200;Seita.9G437300* | | | | |
| GO:0005886 | plasma membrane | cellular_component | 48 | 442 | 2374 | *Seita.1G014900;Seita.1G198500;Seita.1G202200;Seita.1G231800;Seita.1G246000;Seita.1G268900;Seita.1G317400;Seita.1G327300;Seita.2G090600;Seita.2G238400;Seita.2G289600;Seita.2G291500;Seita.2G428900;Seita.2G434600;Seita.3G047600;Seita.3G048500;Seita.3G068600;Seita.3G078700;Seita.3G083300;Seita.3G088000;Seita.3G093400;Seita.3G196200;Seita.4G003100;Seita.4G042500;Seita.4G138500;Seita.4G232700;Seita.4G274900;Seita.5G010800;Seita.5G203200;Seita.5G203400;Seita.5G281400;Seita.5G351800;Seita.5G442200;Seita.6G136800;Seita.6G159400;Seita.6G165800;Seita.6G186400;Seita.6G235000;Seita.7G014100;Seita.8G022900;Seita.8G027000;Seita.8G152800;Seita.9G277500;Seita.9G373200;Seita.9G437300;Seita.9G456100;Seita.9G504800;Seita.9G505000* | | | | |
| GO:0006749 | glutathione metabolic process | biological_process | 1 | 442 | 77 | *Seita.9G064500* | | | | |
| GO:0031225 | anchored component of membrane | cellular_component | 3 | 442 | 200 | *Seita.4G003100;Seita.5G203400;Seita.6G070700* | | | | |
| GO:0006897 | endocytosis | biological_process | 1 | 442 | 79 | *Seita.2G434600* | | | | |
| GO:0050661 | NADP binding | molecular_function | 1 | 442 | 80 | *Seita.9G365500* | | | | |
| GO:0009624 | response to nematode | biological_process | 1 | 442 | 82 | *Seita.3G093400* | | | | |
| GO:0016607 | nuclear speck | cellular_component | 1 | 442 | 82 | *Seita.7G179600* | | | | |
| GO:0005777 | peroxisome | cellular_component | 2 | 442 | 147 | *Seita.3G020400;Seita.3G020600* | | | | |
| GO:0009658 | chloroplast organization | biological_process | 2 | 442 | 148 | *Seita.1G292500;Seita.9G525000* | | | | |
| GO:0015333 | peptide:proton symporter activity | molecular_function | 1 | 442 | 84 | *Seita.3G093400* | | | | |
| GO:0045735 | nutrient reservoir activity | molecular_function | 1 | 442 | 84 | *Seita.3G110500* | | | | |
| GO:1904680 | peptide transmembrane transporter activity | molecular_function | 1 | 442 | 84 | *Seita.3G093400* | | | | |
| GO:0009813 | flavonoid biosynthetic process | biological_process | 1 | 442 | 87 | *Seita.9G561500* | | | | |
| GO:0009534 | chloroplast thylakoid | cellular_component | 1 | 442 | 90 | *Seita.5G099500* | | | | |
| GO:0048364 | root development | biological_process | 2 | 442 | 157 | *Seita.3G088000;Seita.5G034500* | | | | |
| GO:0010008 | endosome membrane | cellular_component | 1 | 442 | 94 | *Seita.2G200700* | | | | |
| GO:0005759 | mitochondrial matrix | cellular_component | 1 | 442 | 96 | *Seita.3G382100* | | | | |
| GO:0005773 | vacuole | cellular_component | 6 | 442 | 408 | *Seita.1G054800;Seita.2G038200;Seita.2G134400;Seita.2G251700;Seita.2G291500;Seita.9G440100* | | | | |
| GO:0009579 | thylakoid | cellular_component | 1 | 442 | 103 | *Seita.5G099500* | | | | |
| GO:0005768 | endosome | cellular_component | 3 | 442 | 237 | *Seita.2G434600;Seita.8G122400;Seita.9G456100* | | | | |
| GO:0015297 | antiporter activity | molecular_function | 1 | 442 | 104 | *Seita.2G001400* | | | | |
| GO:0005802 | trans-Golgi network | cellular_component | 3 | 442 | 239 | *Seita.1G268900;Seita.8G122400;Seita.9G456100* | | | | |
| GO:0009870 | defense response signaling pathway, resistance gene-dependent | biological_process | 1 | 442 | 105 | *Seita.7G131700* | | | | |
| GO:0046686 | response to cadmium ion | biological_process | 2 | 442 | 179 | *Seita.2G140900;Seita.3G124200* | | | | |
| GO:0004364 | glutathione transferase activity | molecular_function | 1 | 442 | 108 | *Seita.9G064500* | | | | |
| GO:0005774 | vacuolar membrane | cellular_component | 6 | 442 | 420 | *Seita.1G054800;Seita.1G268000;Seita.2G251700;Seita.4G003100;Seita.4G138500;Seita.9G478600* | | | | |
| GO:0009617 | response to bacterium | biological_process | 1 | 442 | 110 | *Seita.4G067200* | | | | |
| GO:0005789 | endoplasmic reticulum membrane | cellular_component | 9 | 442 | 594 | *Seita.1G246000;Seita.1G292500;Seita.2G302500;Seita.5G095900;Seita.5G281400;Seita.5G442200;Seita.6G184400;Seita.7G014100;Seita.7G323700* | | | | |
| GO:0004252 | serine-type endopeptidase activity | molecular_function | 1 | 442 | 115 | *Seita.9G081900* | | | | |
| GO:0009555 | pollen development | biological_process | 1 | 442 | 121 | *Seita.6G198600* | | | | |
| GO:0009506 | plasmodesma | cellular_component | 11 | 442 | 724 | *Seita.2G106500;Seita.2G227900;Seita.2G291500;Seita.3G105100;Seita.4G003100;Seita.4G100500;Seita.6G165800;Seita.6G235000;Seita.8G128400;Seita.9G456100;Seita.J003100* | | | | |
| GO:0050832 | defense response to fungus | biological_process | 3 | 442 | 265 | *Seita.2G134400;Seita.2G140900;Seita.9G079200* | | | | |
| GO:0046777 | protein autophosphorylation | biological_process | 1 | 442 | 125 | *Seita.1G246000* | | | | |
| GO:0042631 | cellular response to water deprivation | biological_process | 1 | 442 | 126 | *Seita.6G136200* | | | | |
| GO:0006486 | protein glycosylation | biological_process | 1 | 442 | 127 | *Seita.9G542400* | | | | |
| GO:0019843 | rRNA binding | molecular_function | 1 | 442 | 128 | *Seita.9G004600* | | | | |
| GO:0016788 | hydrolase activity, acting on ester bonds | molecular_function | 1 | 442 | 131 | *Seita.3G075300* | | | | |
| GO:0000139 | Golgi membrane | cellular_component | 6 | 442 | 464 | *Seita.1G054000;Seita.1G268900;Seita.1G294300;Seita.5G303500;Seita.7G274700;Seita.8G122400* | | | | |
| GO:0042802 | identical protein binding | molecular_function | 2 | 442 | 217 | *Seita.6G136200;Seita.9G552100* | | | | |
| GO:0071472 | cellular response to salt stress | biological_process | 1 | 442 | 139 | *Seita.6G136200* | | | | |
| GO:0030246 | carbohydrate binding | molecular_function | 1 | 442 | 140 | *Seita.3G047600* | | | | |
| GO:0009705 | plant-type vacuole membrane | cellular_component | 1 | 442 | 142 | *Seita.3G300700* | | | | |
| GO:0009535 | chloroplast thylakoid membrane | cellular_component | 4 | 442 | 358 | *Seita.5G067800;Seita.5G099500;Seita.9G344400;Seita.9G552100* | | | | |
| GO:0000287 | magnesium ion binding | molecular_function | 1 | 442 | 145 | *Seita.1G171900* | | | | |
| GO:0008017 | microtubule binding | molecular_function | 1 | 442 | 147 | *Seita.8G215500* | | | | |
| GO:0009626 | plant-type hypersensitive response | biological_process | 1 | 442 | 153 | *Seita.7G131700* | | | | |
| GO:0016021 | integral component of membrane | cellular_component | 75 | 442 | 4005 | *Seita.1G004600;Seita.1G014900;Seita.1G054000;Seita.1G054800;Seita.1G186400;Seita.1G198500;Seita.1G202200;Seita.1G246000;Seita.1G268000;Seita.1G268900;Seita.1G271500;Seita.1G288100;Seita.1G294300;Seita.1G317400;Seita.1G327300;Seita.2G001400;Seita.2G053300;Seita.2G090600;Seita.2G094000;Seita.2G116100;Seita.2G229900;Seita.2G238400;Seita.2G251700;Seita.2G291500;Seita.2G302500;Seita.2G404300;Seita.3G047600;Seita.3G068600;Seita.3G083300;Seita.3G093400;Seita.3G164400;Seita.3G227200;Seita.3G339800;Seita.4G042500;Seita.4G138500;Seita.4G194900;Seita.4G225400;Seita.4G232700;Seita.4G274900;Seita.5G033400;Seita.5G033500;Seita.5G095900;Seita.5G203200;Seita.5G281400;Seita.5G303500;Seita.5G327000;Seita.5G351800;Seita.5G379400;Seita.5G442200;Seita.6G023100;Seita.6G165800;Seita.6G184400;Seita.6G186400;Seita.7G014100;Seita.7G032900;Seita.7G209000;Seita.7G274700;Seita.7G323700;Seita.8G022900;Seita.8G027000;Seita.8G122400;Seita.8G152800;Seita.9G050000;Seita.9G190200;Seita.9G254900;Seita.9G277500;Seita.9G311400;Seita.9G344400;Seita.9G373200;Seita.9G440500;Seita.9G469300;Seita.9G478600;Seita.9G520900;Seita.9G542400;Seita.9G555000* | | | | |
| GO:0005794 | Golgi apparatus | cellular_component | 10 | 442 | 767 | *Seita.1G231800;Seita.1G268900;Seita.1G294300;Seita.4G176600;Seita.4G246200;Seita.6G165800;Seita.7G274700;Seita.8G122400;Seita.9G298200;Seita.9G456100* | | | | |
| GO:0003924 | GTPase activity | molecular_function | 1 | 442 | 186 | *Seita.2G335500* | | | | |
| GO:0005525 | GTP binding | molecular_function | 2 | 442 | 273 | *Seita.2G335500;Seita.3G216400* | | | | |
| GO:0009507 | chloroplast | cellular_component | 25 | 442 | 1615 | *Seita.1G171900;Seita.1G216200;Seita.1G288400;Seita.1G349400;Seita.2G035400;Seita.2G232900;Seita.2G327500;Seita.3G046400;Seita.3G124300;Seita.3G144600;Seita.3G216400;Seita.4G031400;Seita.4G067200;Seita.4G281200;Seita.5G058800;Seita.5G065300;Seita.5G099500;Seita.6G182600;Seita.6G182700;Seita.8G247600;Seita.9G079200;Seita.9G156500;Seita.9G229700;Seita.9G344400;Seita.9G365500* | | | | |
| GO:0005829 | cytosol | cellular_component | 27 | 442 | 1737 | *Seita.1G231800;Seita.1G347300;Seita.1G349700;Seita.2G217500;Seita.2G236200;Seita.2G401300;Seita.3G007200;Seita.3G076200;Seita.3G138700;Seita.3G147500;Seita.4G176600;Seita.4G226700;Seita.5G027400;Seita.5G287300;Seita.5G315500;Seita.5G318400;Seita.6G210000;Seita.6G235000;Seita.7G105000;Seita.8G074800;Seita.9G079200;Seita.9G137200;Seita.9G216200;Seita.9G238100;Seita.9G417000;Seita.9G427100;Seita.9G440100* | | | | |
| GO:0009793 | embryo development ending in seed dormancy | biological_process | 1 | 442 | 210 | *Seita.9G079200* | | | | |
| GO:0006886 | intracellular protein transport | biological_process | 1 | 442 | 217 | *Seita.2G335500* | | | | |
| GO:0005783 | endoplasmic reticulum | cellular_component | 8 | 442 | 725 | *Seita.1G292500;Seita.1G317400;Seita.1G362000;Seita.2G106500;Seita.9G050000;Seita.9G234200;Seita.9G440100;Seita.9G461800* | | | | |
| GO:0005524 | ATP binding | molecular_function | 42 | 442 | 2580 | *Seita.1G239600;Seita.1G246000;Seita.2G056400;Seita.2G057000;Seita.2G200700;Seita.2G206200;Seita.2G213800;Seita.2G432000;Seita.3G047600;Seita.3G053800;Seita.3G076300;Seita.3G083300;Seita.3G138700;Seita.3G145200;Seita.3G160800;Seita.3G196200;Seita.3G284500;Seita.3G380200;Seita.3G382100;Seita.4G232700;Seita.5G203200;Seita.5G281400;Seita.5G284300;Seita.5G315500;Seita.5G376100;Seita.6G163200;Seita.6G200300;Seita.7G131700;Seita.7G241600;Seita.7G300300;Seita.8G022900;Seita.8G025400;Seita.8G152800;Seita.8G215500;Seita.8G242600;Seita.8G242800;Seita.8G243800;Seita.8G244000;Seita.9G050000;Seita.9G190200;Seita.9G198200;Seita.9G440300* | | | | |
| GO:0003729 | mRNA binding | molecular_function | 1 | 442 | 244 | *Seita.9G417000* | | | | |
| GO:0005739 | mitochondrion | cellular_component | 10 | 442 | 898 | *Seita.1G117500;Seita.1G310800;Seita.1G326600;Seita.1G347300;Seita.4G100500;Seita.5G095900;Seita.5G157600;Seita.8G128400;Seita.9G050000;Seita.9G216200* | | | | |
| GO:0051301 | cell division | biological_process | 1 | 442 | 253 | *Seita.3G251400* | | | | |
| GO:0015031 | protein transport | biological_process | 1 | 442 | 287 | *Seita.6G193400* | | | | |
| GO:0005730 | nucleolus | cellular_component | 1 | 442 | 409 | *Seita.4G227700* | | | | |
| GO:0003723 | RNA binding | molecular_function | 3 | 442 | 654 | *Seita.1G239000;Seita.2G289600;Seita.6G159400* | | | | |
|  |  |  |  |  |  |  |  |  |  |  |

| Table S6. GO enrichment analysis of differentially expressed genes in foxtail millet after 7 days of PET nanoplastics treatment | | | | | | |
| --- | --- | --- | --- | --- | --- | --- |
| id | Term | Category | ListHits | ListTotal | PopHits | geneID |
| GO:0003700 | DNA-binding transcription factor activity | molecular_function | 32 | 148 | 1254 | *Seita.1G062100;Seita.1G217800;Seita.1G270800;Seita.1G271000;Seita.1G290700;Seita.1G341100;Seita.2G140900;Seita.2G280000;Seita.2G280100;Seita.2G280200;Seita.2G280400;Seita.3G108500;Seita.3G164900;Seita.3G206900;Seita.3G225400;Seita.4G016400;Seita.4G093200;Seita.4G227700;Seita.5G022000;Seita.5G132800;Seita.5G294500;Seita.5G391200;Seita.6G023700;Seita.6G023800;Seita.7G112000;Seita.7G124900;Seita.7G205200;Seita.8G123100;Seita.9G069700;Seita.9G320700;Seita.9G323300;Seita.9G509400* |
| GO:0010200 | response to chitin | biological_process | 9 | 148 | 128 | *Seita.1G062100;Seita.1G186200;Seita.1G186400;Seita.1G327300;Seita.2G140900;Seita.3G066800;Seita.5G363900;Seita.7G112000;Seita.9G320700* |
| GO:0009873 | ethylene-activated signaling pathway | biological_process | 11 | 148 | 215 | *Seita.1G270800;Seita.1G341100;Seita.4G227700;Seita.5G022000;Seita.6G023700;Seita.6G023800;Seita.6G170000;Seita.6G170200;Seita.9G320700;Seita.9G323300;Seita.9G509400* |
| GO:0009867 | jasmonic acid mediated signaling pathway | biological_process | 4 | 148 | 37 | *Seita.3G108500;Seita.3G164900;Seita.5G132800;Seita.5G294500* |
| GO:0043565 | sequence-specific DNA binding | molecular_function | 16 | 148 | 757 | *Seita.1G062100;Seita.1G217800;Seita.1G239400;Seita.1G270800;Seita.2G140900;Seita.3G108500;Seita.3G164900;Seita.3G206900;Seita.4G093200;Seita.4G227700;Seita.5G132800;Seita.5G294500;Seita.6G023700;Seita.6G023800;Seita.7G112000;Seita.9G509400* |
| GO:0045489 | pectin biosynthetic process | biological_process | 4 | 148 | 39 | *Seita.1G314700;Seita.1G336700;Seita.4G108400;Seita.8G008500* |
| GO:0044212 | transcription regulatory region DNA binding | molecular_function | 9 | 148 | 271 | *Seita.1G217800;Seita.1G239400;Seita.1G270800;Seita.2G140900;Seita.4G093200;Seita.6G023700;Seita.6G023800;Seita.8G123100;Seita.9G323300* |
| GO:0050832 | defense response to fungus | biological_process | 8 | 148 | 265 | *Seita.1G270800;Seita.2G140900;Seita.3G108500;Seita.3G164900;Seita.4G154100;Seita.5G132800;Seita.5G294500;Seita.8G143300* |
| GO:0047262 | polygalacturonate 4-alpha-galacturonosyltransferase activity | molecular_function | 3 | 148 | 27 | *Seita.1G314700;Seita.4G108400;Seita.8G008500* |
| GO:0019897 | extrinsic component of plasma membrane | cellular_component | 2 | 148 | 7 | *Seita.6G170000;Seita.6G170200* |
| GO:0051300 | spindle pole body organization | biological_process | 2 | 148 | 7 | *Seita.3G135500;Seita.3G380800* |
| GO:0009414 | response to water deprivation | biological_process | 8 | 148 | 289 | *Seita.1G062100;Seita.2G140900;Seita.3G066800;Seita.4G227700;Seita.6G023700;Seita.6G023800;Seita.9G327900;Seita.9G328000* |
| GO:0009737 | response to abscisic acid | biological_process | 9 | 148 | 358 | *Seita.1G062100;Seita.1G186400;Seita.2G069800;Seita.2G140900;Seita.3G066800;Seita.3G206900;Seita.4G227700;Seita.6G023700;Seita.6G023800* |
| GO:0071555 | cell wall organization | biological_process | 9 | 148 | 360 | *Seita.1G314700;Seita.3G105100;Seita.3G110000;Seita.4G008800;Seita.4G108400;Seita.5G331000;Seita.6G052500;Seita.7G090400;Seita.8G008500* |
| GO:0005509 | calcium ion binding | molecular_function | 7 | 148 | 237 | *Seita.3G135500;Seita.3G261400;Seita.3G380800;Seita.4G255900;Seita.5G451500;Seita.5G456800;Seita.9G456100* |
| GO:0051753 | mannan synthase activity | molecular_function | 2 | 148 | 10 | *Seita.4G008800;Seita.6G052500* |
| GO:0009815 | 1-aminocyclopropane-1-carboxylate oxidase activity | molecular_function | 2 | 148 | 11 | *Seita.2G225500;Seita.3G037900* |
| GO:0005199 | structural constituent of cell wall | molecular_function | 2 | 148 | 12 | *Seita.3G105100;Seita.3G110000* |
| GO:0009827 | plant-type cell wall modification | biological_process | 2 | 148 | 12 | *Seita.1G336700;Seita.2G418500* |
| GO:0030244 | cellulose biosynthetic process | biological_process | 3 | 148 | 45 | *Seita.1G336700;Seita.4G008800;Seita.6G052500* |
| GO:0009816 | defense response to bacterium, incompatible interaction | biological_process | 3 | 148 | 52 | *Seita.1G186400;Seita.1G327300;Seita.3G008300* |
| GO:0009938 | negative regulation of gibberellic acid mediated signaling pathway | biological_process | 2 | 148 | 17 | *Seita.1G062100;Seita.3G206900* |
| GO:0015706 | nitrate transport | biological_process | 3 | 148 | 53 | *Seita.5G326900;Seita.9G327900;Seita.9G328000* |
| GO:0009635 | response to herbicide | biological_process | 2 | 148 | 18 | *Seita.9G327900;Seita.9G328000* |
| GO:0009506 | plasmodesma | cellular_component | 12 | 148 | 724 | *Seita.1G170200;Seita.3G008300;Seita.3G105100;Seita.3G110000;Seita.3G406500;Seita.4G022900;Seita.6G093100;Seita.6G165800;Seita.7G239900;Seita.7G296300;Seita.9G434000;Seita.9G456100* |
| GO:0005768 | endosome | cellular_component | 6 | 148 | 237 | *Seita.1G336700;Seita.2G434600;Seita.7G239900;Seita.8G008500;Seita.9G434000;Seita.9G456100* |
| GO:0004842 | ubiquitin-protein transferase activity | molecular_function | 7 | 148 | 310 | *Seita.1G186200;Seita.1G186400;Seita.1G197500;Seita.1G327300;Seita.3G066800;Seita.5G363900;Seita.8G143300* |
| GO:0009739 | response to gibberellin | biological_process | 4 | 148 | 109 | *Seita.1G062100;Seita.2G140900;Seita.3G206900;Seita.8G143300* |
| GO:0003677 | DNA binding | molecular_function | 19 | 148 | 1401 | *Seita.1G271000;Seita.1G290700;Seita.1G341100;Seita.2G086600;Seita.2G280000;Seita.2G280100;Seita.2G280200;Seita.2G280400;Seita.3G225400;Seita.4G016400;Seita.4G148800;Seita.5G022000;Seita.5G391200;Seita.7G124900;Seita.7G205200;Seita.9G069700;Seita.9G080500;Seita.9G169700;Seita.9G320700* |
| GO:0005802 | trans-Golgi network | cellular_component | 6 | 148 | 239 | *Seita.1G336700;Seita.4G008800;Seita.6G052500;Seita.8G008500;Seita.9G434000;Seita.9G456100* |
| GO:0030234 | enzyme regulator activity | molecular_function | 2 | 148 | 20 | *Seita.3G135500;Seita.3G380800* |
| GO:0030307 | positive regulation of cell growth | biological_process | 2 | 148 | 20 | *Seita.6G170000;Seita.6G170200* |
| GO:0008194 | UDP-glycosyltransferase activity | molecular_function | 4 | 148 | 111 | *Seita.2G276500;Seita.8G153700;Seita.9G086100;Seita.9G086800* |
| GO:0016709 | oxidoreductase activity,acting on paired donors, with incorporation or reduction of molecular oxygen, NAD(P)H as one donor, and incorporation of one atom of oxygen | molecular_function | 4 | 148 | 111 | *Seita.6G003900;Seita.6G004000;Seita.6G004100;Seita.6G023100* |
| GO:0042742 | defense response to bacterium | biological_process | 7 | 148 | 317 | *Seita.2G140900;Seita.3G008300;Seita.3G047600;Seita.3G164900;Seita.5G294500;Seita.7G239900;Seita.8G143300* |
| GO:0010167 | response to nitrate | biological_process | 2 | 148 | 21 | *Seita.9G327900;Seita.9G328000* |
| GO:0015112 | nitrate transmembrane transporter activity | molecular_function | 2 | 148 | 21 | *Seita.9G327900;Seita.9G328000* |
| GO:0009733 | response to auxin | biological_process | 5 | 148 | 180 | *Seita.2G140900;Seita.4G246400;Seita.4G255900;Seita.6G170000;Seita.6G170200* |
| GO:0005992 | trehalose biosynthetic process | biological_process | 2 | 148 | 23 | *Seita.2G188200;Seita.9G069700* |
| GO:0009693 | ethylene biosynthetic process | biological_process | 2 | 148 | 23 | *Seita.2G225500;Seita.3G037900* |
| GO:0046658 | anchored component of plasma membrane | cellular_component | 5 | 148 | 186 | *Seita.2G242700;Seita.3G008300;Seita.7G090400;Seita.7G296300;Seita.9G434000* |
| GO:0005794 | Golgi apparatus | cellular_component | 12 | 148 | 767 | *Seita.1G314700;Seita.1G336700;Seita.3G008300;Seita.4G008800;Seita.4G108400;Seita.4G246400;Seita.6G052500;Seita.6G165800;Seita.7G227400;Seita.8G008500;Seita.9G434000;Seita.9G456100* |
| GO:0009814 | defense response, incompatible interaction | biological_process | 2 | 148 | 24 | *Seita.1G186400;Seita.1G327300* |
| GO:0009835 | fruit ripening | biological_process | 2 | 148 | 25 | *Seita.2G225500;Seita.3G037900* |
| GO:0020037 | heme binding | molecular_function | 9 | 148 | 508 | *Seita.1G170200;Seita.3G004800;Seita.5G009900;Seita.6G003900;Seita.6G004000;Seita.6G004100;Seita.6G023100;Seita.7G128200;Seita.7G206600* |
| GO:0031225 | anchored component of membrane | cellular_component | 5 | 148 | 200 | *Seita.2G242700;Seita.2G249000;Seita.2G249200;Seita.3G077900;Seita.9G434000* |
| GO:0051607 | defense response to virus | biological_process | 2 | 148 | 28 | *Seita.3G008300;Seita.7G296300* |
| GO:0009833 | plant-type primary cell wall biogenesis | biological_process | 2 | 148 | 31 | *Seita.4G008800;Seita.6G052500* |
| GO:0005618 | cell wall | cellular_component | 8 | 148 | 456 | *Seita.2G242700;Seita.3G105100;Seita.3G110000;Seita.4G246400;Seita.5G346700;Seita.6G159400;Seita.7G090400;Seita.7G227400* |
| GO:0080043 | quercetin 3-O-glucosyltransferase activity | molecular_function | 3 | 148 | 81 | *Seita.8G153700;Seita.9G086100;Seita.9G086800* |
| GO:0080044 | quercetin 7-O-glucosyltransferase activity | molecular_function | 3 | 148 | 81 | *Seita.8G153700;Seita.9G086100;Seita.9G086800* |
| GO:0016760 | cellulose synthase (UDP-forming) activity | molecular_function | 2 | 148 | 33 | *Seita.4G008800;Seita.6G052500* |
| GO:0006952 | defense response | biological_process | 13 | 148 | 939 | *Seita.1G062100;Seita.2G434600;Seita.3G047600;Seita.5G377300;Seita.6G017500;Seita.6G023100;Seita.6G023700;Seita.6G023800;Seita.6G048500;Seita.7G090400;Seita.7G094400;Seita.8G042800;Seita.9G471800* |
| GO:0015293 | symporter activity | molecular_function | 3 | 148 | 89 | *Seita.3G024600;Seita.9G327900;Seita.9G328000* |
| GO:0035251 | UDP-glucosyltransferase activity | molecular_function | 2 | 148 | 36 | *Seita.1G208100;Seita.8G153700* |
| GO:0016762 | xyloglucan:xyloglucosyl transferase activity | molecular_function | 2 | 148 | 37 | *Seita.4G246400;Seita.7G227400* |
| GO:0009718 | anthocyanin-containing compound biosynthetic process | biological_process | 2 | 148 | 38 | *Seita.6G159400;Seita.9G069700* |
| GO:0005886 | plasma membrane | cellular_component | 26 | 148 | 2374 | *Seita.1G197500;Seita.1G327300;Seita.2G242700;Seita.2G249000;Seita.2G249200;Seita.2G418500;Seita.2G434600;Seita.3G008300;Seita.3G024600;Seita.3G047600;Seita.3G048400;Seita.3G077900;Seita.3G406500;Seita.4G008800;Seita.4G154100;Seita.5G326900;Seita.5G377300;Seita.5G381400;Seita.6G052500;Seita.6G159400;Seita.6G165800;Seita.7G227400;Seita.7G239900;Seita.9G327900;Seita.9G328000;Seita.9G456100* |
| GO:0010067 | procambium histogenesis | biological_process | 1 | 148 | 5 | *Seita.1G290700* |
| GO:0040020 | regulation of meiotic nuclear division | biological_process | 1 | 148 | 5 | *Seita.1G217800* |
| GO:0046513 | ceramide biosynthetic process | biological_process | 1 | 148 | 5 | *Seita.7G177500* |
| GO:0090448 | glucosinolate:proton symporter activity | molecular_function | 1 | 148 | 5 | *Seita.3G406500* |
| GO:0090449 | phloem glucosinolate loading | biological_process | 1 | 148 | 5 | *Seita.3G406500* |
| GO:1901349 | glucosinolate transport | biological_process | 1 | 148 | 5 | *Seita.3G406500* |
| GO:0019722 | calcium-mediated signaling | biological_process | 2 | 148 | 41 | *Seita.3G135500;Seita.3G380800* |
| GO:0031418 | L-ascorbic acid binding | molecular_function | 2 | 148 | 41 | *Seita.2G225500;Seita.3G037900* |
| GO:0030163 | protein catabolic process | biological_process | 3 | 148 | 100 | *Seita.2G249000;Seita.2G249200;Seita.5G378700* |
| GO:0006508 | proteolysis | biological_process | 3 | 148 | 101 | *Seita.2G249000;Seita.2G249200;Seita.5G378700* |
| GO:0000304 | response to singlet oxygen | biological_process | 1 | 148 | 6 | *Seita.2G069800* |
| GO:0005930 | axoneme | cellular_component | 1 | 148 | 6 | *Seita.2G352400* |
| GO:0005968 | Rab-protein geranylgeranyltransferase complex | cellular_component | 1 | 148 | 6 | *Seita.6G128200* |
| GO:0006561 | proline biosynthetic process | biological_process | 1 | 148 | 6 | *Seita.9G069700* |
| GO:0030286 | dynein complex | cellular_component | 1 | 148 | 6 | *Seita.2G352400* |
| GO:0044458 | motile cilium assembly | biological_process | 1 | 148 | 6 | *Seita.2G352400* |
| GO:0052544 | defense response by callose deposition in cell wall | biological_process | 1 | 148 | 6 | *Seita.7G239900* |
| GO:0097014 | ciliary plasm | cellular_component | 1 | 148 | 6 | *Seita.2G352400* |
| GO:0004190 | aspartic-type endopeptidase activity | molecular_function | 3 | 148 | 104 | *Seita.2G249000;Seita.2G249200;Seita.5G378700* |
| GO:0004553 | hydrolase activity, hydrolyzing O-glycosyl compounds | molecular_function | 2 | 148 | 46 | *Seita.4G246400;Seita.7G227400* |
| GO:0009617 | response to bacterium | biological_process | 3 | 148 | 110 | *Seita.1G062100;Seita.3G008300;Seita.9G471800* |
| GO:0000077 | DNA damage checkpoint | biological_process | 1 | 148 | 7 | *Seita.1G217800* |
| GO:0003883 | CTP synthase activity | molecular_function | 1 | 148 | 7 | *Seita.3G138700* |
| GO:0004540 | ribonuclease activity | molecular_function | 1 | 148 | 7 | *Seita.6G159400* |
| GO:0009612 | response to mechanical stimulus | biological_process | 1 | 148 | 7 | *Seita.4G246400* |
| GO:0010048 | vernalization response | biological_process | 1 | 148 | 7 | *Seita.4G093200* |
| GO:0018344 | protein geranylgeranylation | biological_process | 1 | 148 | 7 | *Seita.6G128200* |
| GO:0033897 | ribonuclease T2 activity | molecular_function | 1 | 148 | 7 | *Seita.6G159400* |
| GO:0044210 | 'de novo' CTP biosynthetic process | biological_process | 1 | 148 | 7 | *Seita.3G138700* |
| GO:0046488 | phosphatidylinositol metabolic process | biological_process | 1 | 148 | 7 | *Seita.2G418500* |
| GO:0047938 | glucose-6-phosphate 1-epimerase activity | molecular_function | 1 | 148 | 7 | *Seita.5G251100* |
| GO:0080054 | low-affinity nitrate transmembrane transporter activity | molecular_function | 1 | 148 | 7 | *Seita.5G326900* |
| GO:0102960 | momilactone-A synthase activity | molecular_function | 1 | 148 | 7 | *Seita.2G375000* |
| GO:0016021 | integral component of membrane | cellular_component | 39 | 148 | 4005 | *Seita.1G025100;Seita.1G186400;Seita.1G197500;Seita.1G270500;Seita.1G314500;Seita.1G314700;Seita.1G327300;Seita.1G336700;Seita.2G069800;Seita.3G008300;Seita.3G024600;Seita.3G047600;Seita.3G406500;Seita.4G004900;Seita.4G008800;Seita.4G108400;Seita.4G154100;Seita.4G194900;Seita.4G225400;Seita.5G009900;Seita.5G326900;Seita.5G331000;Seita.6G003900;Seita.6G004000;Seita.6G004100;Seita.6G023100;Seita.6G052500;Seita.6G165800;Seita.7G177500;Seita.7G204900;Seita.7G206600;Seita.7G216000;Seita.7G239900;Seita.7G296300;Seita.8G008500;Seita.9G190200;Seita.9G224200;Seita.9G327900;Seita.9G328000* |
| GO:0005506 | iron ion binding | molecular_function | 6 | 148 | 354 | *Seita.5G009900;Seita.6G003900;Seita.6G004000;Seita.6G004100;Seita.6G023100;Seita.7G206600* |
| GO:0042128 | nitrate assimilation | biological_process | 2 | 148 | 49 | *Seita.9G327900;Seita.9G328000* |
| GO:0010332 | response to gamma radiation | biological_process | 1 | 148 | 8 | *Seita.1G217800* |
| GO:0017101 | aminoacyl-tRNA synthetase multienzyme complex | cellular_component | 1 | 148 | 8 | *Seita.6G093100* |
| GO:0030100 | regulation of endocytosis | biological_process | 1 | 148 | 8 | *Seita.2G434600* |
| GO:0031514 | motile cilium | cellular_component | 1 | 148 | 8 | *Seita.2G352400* |
| GO:0034059 | response to anoxia | biological_process | 1 | 148 | 8 | *Seita.5G022000* |
| GO:0043069 | negative regulation of programmed cell death | biological_process | 1 | 148 | 8 | *Seita.8G143300* |
| GO:0052634 | C-19 gibberellin 2-beta-dioxygenase activity | molecular_function | 1 | 148 | 8 | *Seita.3G148100* |
| GO:0005634 | nucleus | cellular_component | 46 | 148 | 4924 | *Seita.1G062100;Seita.1G217800;Seita.1G239400;Seita.1G270800;Seita.1G271000;Seita.1G290700;Seita.1G341100;Seita.2G086600;Seita.2G140900;Seita.2G280000;Seita.2G280100;Seita.2G280200;Seita.2G280400;Seita.3G066800;Seita.3G108500;Seita.3G135500;Seita.3G163300;Seita.3G164900;Seita.3G206900;Seita.3G225400;Seita.3G380800;Seita.4G016400;Seita.4G093200;Seita.4G148800;Seita.5G022000;Seita.5G132800;Seita.5G294500;Seita.5G363900;Seita.5G391200;Seita.6G023700;Seita.6G023800;Seita.6G170000;Seita.6G170200;Seita.7G090400;Seita.7G094400;Seita.7G112000;Seita.7G124900;Seita.7G205200;Seita.8G123100;Seita.8G143300;Seita.9G069700;Seita.9G080500;Seita.9G169700;Seita.9G320700;Seita.9G323300;Seita.9G509400* |
| GO:0010120 | camalexin biosynthetic process | biological_process | 1 | 148 | 9 | *Seita.9G069700* |
| GO:0016308 | 1-phosphatidylinositol-4-phosphate 5-kinase activity | molecular_function | 1 | 148 | 9 | *Seita.2G418500* |
| GO:0009740 | gibberellic acid mediated signaling pathway | biological_process | 2 | 148 | 57 | *Seita.1G062100;Seita.3G206900* |
| GO:0010411 | xyloglucan metabolic process | biological_process | 2 | 148 | 57 | *Seita.4G246400;Seita.7G227400* |
| GO:0016324 | apical plasma membrane | cellular_component | 1 | 148 | 10 | *Seita.2G418500* |
| GO:1990841 | promoter-specific chromatin binding | molecular_function | 1 | 148 | 10 | *Seita.1G062100* |
| GO:0042546 | cell wall biogenesis | biological_process | 2 | 148 | 61 | *Seita.4G246400;Seita.7G227400* |
| GO:0007264 | small GTPase mediated signal transduction | biological_process | 1 | 148 | 11 | *Seita.6G128200* |
| GO:0010333 | terpene synthase activity | molecular_function | 1 | 148 | 11 | *Seita.6G048500* |
| GO:0045487 | gibberellin catabolic process | biological_process | 1 | 148 | 11 | *Seita.3G148100* |
| GO:0045543 | gibberellin 2-beta-dioxygenase activity | molecular_function | 1 | 148 | 11 | *Seita.3G148100* |
| GO:0050505 | hydroquinone glucosyltransferase activity | molecular_function | 1 | 148 | 11 | *Seita.8G153700* |
| GO:0004805 | trehalose-phosphatase activity | molecular_function | 1 | 148 | 12 | *Seita.2G188200* |
| GO:0006898 | receptor-mediated endocytosis | biological_process | 1 | 148 | 12 | *Seita.7G239900* |
| GO:0043562 | cellular response to nitrogen levels | biological_process | 1 | 148 | 12 | *Seita.1G186400* |
| GO:0006855 | drug transmembrane transport | biological_process | 2 | 148 | 66 | *Seita.1G270500;Seita.7G204900* |
| GO:0030001 | metal ion transport | biological_process | 2 | 148 | 66 | *Seita.5G299000;Seita.9G537500* |
| GO:0015238 | drug transmembrane transporter activity | molecular_function | 2 | 148 | 68 | *Seita.1G270500;Seita.7G204900* |
| GO:0009620 | response to fungus | biological_process | 2 | 148 | 70 | *Seita.1G062100;Seita.2G434600* |
| GO:0009685 | gibberellin metabolic process | biological_process | 1 | 148 | 14 | *Seita.3G148100* |
| GO:0102452 | bisdemethoxycurcumin synthase activity | molecular_function | 1 | 148 | 14 | *Seita.9G142900* |
| GO:0043231 | intracellular membrane-bounded organelle | cellular_component | 3 | 148 | 148 | *Seita.8G153700;Seita.9G086100;Seita.9G086800* |
| GO:0005544 | calcium-dependent phospholipid binding | molecular_function | 1 | 148 | 15 | *Seita.5G377300* |
| GO:0009623 | response to parasitic fungus | biological_process | 1 | 148 | 15 | *Seita.1G062100* |
| GO:0010215 | cellulose microfibril organization | biological_process | 1 | 148 | 15 | *Seita.9G434000* |
| GO:0010929 | positive regulation of auxin mediated signaling pathway | biological_process | 1 | 148 | 15 | *Seita.2G140900* |
| GO:0012501 | programmed cell death | biological_process | 1 | 148 | 15 | *Seita.5G378700* |
| GO:0050829 | defense response to Gram-negative bacterium | biological_process | 1 | 148 | 15 | *Seita.6G165800* |
| GO:0098754 | detoxification | biological_process | 1 | 148 | 15 | *Seita.2G276500* |
| GO:1900150 | regulation of defense response to fungus | biological_process | 2 | 148 | 76 | *Seita.2G140900;Seita.2G434600* |
| GO:0004857 | enzyme inhibitor activity | molecular_function | 1 | 148 | 16 | *Seita.9G196800* |
| GO:0015267 | channel activity | molecular_function | 1 | 148 | 16 | *Seita.1G025100* |
| GO:0016853 | isomerase activity | molecular_function | 1 | 148 | 16 | *Seita.6G048500* |
| GO:0009751 | response to salicylic acid | biological_process | 3 | 148 | 156 | *Seita.1G062100;Seita.2G140900;Seita.3G008300* |
| GO:0005576 | extracellular region | cellular_component | 11 | 148 | 964 | *Seita.1G170200;Seita.2G038200;Seita.3G004800;Seita.3G105100;Seita.3G110000;Seita.5G346700;Seita.6G159400;Seita.7G090400;Seita.7G099400;Seita.7G128200;Seita.8G033400* |
| GO:0006897 | endocytosis | biological_process | 2 | 148 | 79 | *Seita.2G418500;Seita.2G434600* |
| GO:0002229 | defense response to oomycetes | biological_process | 1 | 148 | 17 | *Seita.3G047600* |
| GO:0010152 | pollen maturation | biological_process | 1 | 148 | 17 | *Seita.4G093200* |
| GO:0080092 | regulation of pollen tube growth | biological_process | 1 | 148 | 17 | *Seita.4G093200* |
| GO:1900056 | negative regulation of leaf senescence | biological_process | 1 | 148 | 17 | *Seita.9G069700* |
| GO:0009723 | response to ethylene | biological_process | 3 | 148 | 161 | *Seita.1G062100;Seita.2G140900;Seita.9G471800* |
| GO:0016114 | terpenoid biosynthetic process | biological_process | 1 | 148 | 18 | *Seita.6G048500* |
| GO:0004521 | endoribonuclease activity | molecular_function | 1 | 148 | 19 | *Seita.6G159400* |
| GO:0006207 | 'de novo' pyrimidine nucleobase biosynthetic process | biological_process | 1 | 148 | 19 | *Seita.3G138700* |
| GO:0005874 | microtubule | cellular_component | 3 | 148 | 168 | *Seita.2G352400;Seita.3G163300;Seita.7G248700* |
| GO:0009611 | response to wounding | biological_process | 3 | 148 | 168 | *Seita.1G062100;Seita.3G008300;Seita.6G159400* |
| GO:0048046 | apoplast | cellular_component | 5 | 148 | 357 | *Seita.2G242700;Seita.4G022900;Seita.4G246400;Seita.7G227400;Seita.9G340400* |
| GO:0009505 | plant-type cell wall | cellular_component | 4 | 148 | 261 | *Seita.1G170200;Seita.3G110000;Seita.4G022900;Seita.7G227400* |
| GO:0003774 | motor activity | molecular_function | 1 | 148 | 20 | *Seita.2G352400* |
| GO:0080167 | response to karrikin | biological_process | 2 | 148 | 87 | *Seita.3G406500;Seita.4G022900* |
| GO:0000139 | Golgi membrane | cellular_component | 6 | 148 | 464 | *Seita.1G314700;Seita.4G008800;Seita.4G108400;Seita.5G331000;Seita.6G052500;Seita.8G008500* |
| GO:0009409 | response to cold | biological_process | 4 | 148 | 265 | *Seita.1G062100;Seita.4G225400;Seita.4G246400;Seita.9G224200* |
| GO:0006865 | amino acid transport | biological_process | 1 | 148 | 21 | *Seita.3G024600* |
| GO:0045892 | negative regulation of transcription, DNA-templated | biological_process | 3 | 148 | 178 | *Seita.1G062100;Seita.6G023700;Seita.6G023800* |
| GO:0005345 | purine nucleobase transmembrane transporter activity | molecular_function | 1 | 148 | 22 | *Seita.7G216000* |
| GO:0006863 | purine nucleobase transport | biological_process | 1 | 148 | 22 | *Seita.7G216000* |
| GO:0009044 | xylan 1,4-beta-xylosidase activity | molecular_function | 1 | 148 | 22 | *Seita.7G128200* |
| GO:0042744 | hydrogen peroxide catabolic process | biological_process | 3 | 148 | 179 | *Seita.1G170200;Seita.3G004800;Seita.7G128200* |
| GO:0002238 | response to molecule of fungal origin | biological_process | 1 | 148 | 23 | *Seita.1G062100* |
| GO:0007017 | microtubule-based process | biological_process | 1 | 148 | 23 | *Seita.2G352400* |
| GO:0009664 | plant-type cell wall organization | biological_process | 1 | 148 | 23 | *Seita.4G246400* |
| GO:0043067 | regulation of programmed cell death | biological_process | 1 | 148 | 23 | *Seita.8G143300* |
| GO:0006633 | fatty acid biosynthetic process | biological_process | 2 | 148 | 96 | *Seita.4G225400;Seita.7G177500* |
| GO:0006401 | RNA catabolic process | biological_process | 1 | 148 | 25 | *Seita.6G159400* |
| GO:0009863 | salicylic acid mediated signaling pathway | biological_process | 1 | 148 | 25 | *Seita.2G434600* |
| GO:0030544 | Hsp70 protein binding | molecular_function | 1 | 148 | 25 | *Seita.6G170300* |
| GO:0005975 | carbohydrate metabolic process | biological_process | 4 | 148 | 290 | *Seita.4G004900;Seita.5G251100;Seita.5G448400;Seita.7G090400* |
| GO:0006541 | glutamine metabolic process | biological_process | 1 | 148 | 26 | *Seita.3G138700* |
| GO:0009741 | response to brassinosteroid | biological_process | 1 | 148 | 26 | *Seita.4G246400* |
| GO:0019760 | glucosinolate metabolic process | biological_process | 1 | 148 | 26 | *Seita.1G270800* |
| GO:0051225 | spindle assembly | biological_process | 1 | 148 | 26 | *Seita.7G248700* |
| GO:0097167 | circadian regulation of translation | biological_process | 1 | 148 | 26 | *Seita.1G170200* |
| GO:2000280 | regulation of root development | biological_process | 1 | 148 | 26 | *Seita.5G022000* |
| GO:0004601 | peroxidase activity | molecular_function | 3 | 148 | 194 | *Seita.1G170200;Seita.3G004800;Seita.7G128200* |
| GO:0003713 | transcription coactivator activity | molecular_function | 1 | 148 | 27 | *Seita.4G227700* |
| GO:0006662 | glycerol ether metabolic process | biological_process | 1 | 148 | 27 | *Seita.3G089400* |
| GO:0015297 | antiporter activity | molecular_function | 2 | 148 | 104 | *Seita.1G270500;Seita.7G204900* |
| GO:0009636 | response to toxic substance | biological_process | 1 | 148 | 28 | *Seita.8G153700* |
| GO:0010118 | stomatal movement | biological_process | 1 | 148 | 28 | *Seita.2G418500* |
| GO:0016887 | ATPase activity | molecular_function | 3 | 148 | 199 | *Seita.2G069800;Seita.7G248700;Seita.9G190200* |
| GO:0016020 | membrane | cellular_component | 9 | 148 | 850 | *Seita.1G186400;Seita.3G024600;Seita.6G003900;Seita.6G004000;Seita.6G004100;Seita.6G023100;Seita.6G093100;Seita.6G165800;Seita.7G216000* |
| GO:0009686 | gibberellin biosynthetic process | biological_process | 1 | 148 | 29 | *Seita.3G148100* |
| GO:0004675 | transmembrane receptor protein serine/threonine kinase activity | molecular_function | 1 | 148 | 30 | *Seita.3G047600* |
| GO:0002237 | response to molecule of bacterial origin | biological_process | 1 | 148 | 31 | *Seita.1G062100* |
| GO:0042538 | hyperosmotic salinity response | biological_process | 1 | 148 | 31 | *Seita.9G069700* |
| GO:0102336 | 3-oxo-arachidoyl-CoA synthase activity | molecular_function | 1 | 148 | 31 | *Seita.4G225400* |
| GO:0102337 | 3-oxo-cerotoyl-CoA synthase activity | molecular_function | 1 | 148 | 31 | *Seita.4G225400* |
| GO:0102338 | 3-oxo-lignoceronyl-CoA synthase activity | molecular_function | 1 | 148 | 31 | *Seita.4G225400* |
| GO:0102756 | very-long-chain 3-ketoacyl-CoA synthase activity | molecular_function | 1 | 148 | 31 | *Seita.4G225400* |
| GO:0004252 | serine-type endopeptidase activity | molecular_function | 2 | 148 | 115 | *Seita.5G346700;Seita.7G099400* |
| GO:0048367 | shoot system development | biological_process | 1 | 148 | 33 | *Seita.2G242700* |
| GO:0017137 | Rab GTPase binding | molecular_function | 1 | 148 | 34 | *Seita.6G128200* |
| GO:0032922 | circadian regulation of gene expression | biological_process | 1 | 148 | 34 | *Seita.1G170200* |
| GO:0051865 | protein autoubiquitination | biological_process | 1 | 148 | 34 | *Seita.3G066800* |
| GO:0009753 | response to jasmonic acid | biological_process | 2 | 148 | 119 | *Seita.1G062100;Seita.2G140900* |
| GO:0009738 | abscisic acid-activated signaling pathway | biological_process | 3 | 148 | 221 | *Seita.2G140900;Seita.3G066800;Seita.3G206900* |
| GO:0090406 | pollen tube | cellular_component | 1 | 148 | 36 | *Seita.2G418500* |
| GO:0051085 | chaperone cofactor-dependent protein refolding | biological_process | 1 | 148 | 37 | *Seita.6G170300* |
| GO:2000031 | regulation of salicylic acid mediated signaling pathway | biological_process | 1 | 148 | 37 | *Seita.2G140900* |
| GO:0008233 | peptidase activity | molecular_function | 1 | 148 | 38 | *Seita.5G378700* |
| GO:0010087 | phloem or xylem histogenesis | biological_process | 1 | 148 | 38 | *Seita.1G290700* |
| GO:0031347 | regulation of defense response | biological_process | 1 | 148 | 39 | *Seita.1G062100* |
| GO:0000122 | negative regulation of transcription by RNA polymerase II | biological_process | 1 | 148 | 40 | *Seita.8G123100* |
| GO:0016036 | cellular response to phosphate starvation | biological_process | 1 | 148 | 40 | *Seita.6G159400* |
| GO:0000978 | RNA polymerase II proximal promoter sequence-specific DNA binding | molecular_function | 1 | 148 | 42 | *Seita.8G123100* |
| GO:0005615 | extracellular space | cellular_component | 2 | 148 | 136 | *Seita.4G022900;Seita.9G340400* |
| GO:0016874 | ligase activity | molecular_function | 1 | 148 | 44 | *Seita.9G358800* |
| GO:0048527 | lateral root development | biological_process | 1 | 148 | 44 | *Seita.8G123100* |
| GO:0030246 | carbohydrate binding | molecular_function | 2 | 148 | 140 | *Seita.3G047600;Seita.5G251100* |
| GO:0004650 | polygalacturonase activity | molecular_function | 1 | 148 | 45 | *Seita.4G004900* |
| GO:2000022 | regulation of jasmonic acid mediated signaling pathway | biological_process | 1 | 148 | 46 | *Seita.2G140900* |
| GO:0000982 | transcription factor activity, RNA polymerase II proximal promoter sequence-specific DNA binding | molecular_function | 1 | 148 | 47 | *Seita.4G093200* |
| GO:0009788 | negative regulation of abscisic acid-activated signaling pathway | biological_process | 1 | 148 | 47 | *Seita.3G206900* |
| GO:0008017 | microtubule binding | molecular_function | 2 | 148 | 147 | *Seita.3G163300;Seita.7G248700* |
| GO:0009408 | response to heat | biological_process | 2 | 148 | 148 | *Seita.4G227700;Seita.4G246400* |
| GO:0000166 | nucleotide binding | molecular_function | 1 | 148 | 53 | *Seita.2G276500* |
| GO:0048364 | root development | biological_process | 2 | 148 | 157 | *Seita.1G197500;Seita.2G242700* |
| GO:0016705 | oxidoreductase activity, acting on paired donors, with incorporation or reduction of molecular oxygen | molecular_function | 2 | 148 | 159 | *Seita.5G009900;Seita.7G206600* |
| GO:0016758 | transferase activity, transferring hexosyl groups | molecular_function | 1 | 148 | 57 | *Seita.2G276500* |
| GO:0045893 | positive regulation of transcription, DNA-templated | biological_process | 3 | 148 | 278 | *Seita.1G290700;Seita.3G206900;Seita.4G227700* |
| GO:0003993 | acid phosphatase activity | molecular_function | 1 | 148 | 58 | *Seita.8G033400* |
| GO:0005871 | kinesin complex | cellular_component | 1 | 148 | 61 | *Seita.7G248700* |
| GO:0003777 | microtubule motor activity | molecular_function | 1 | 148 | 62 | *Seita.7G248700* |
| GO:0007018 | microtubule-based movement | biological_process | 1 | 148 | 62 | *Seita.7G248700* |
| GO:0006979 | response to oxidative stress | biological_process | 3 | 148 | 291 | *Seita.1G170200;Seita.3G004800;Seita.7G128200* |
| GO:0009846 | pollen germination | biological_process | 1 | 148 | 64 | *Seita.2G418500* |
| GO:0042752 | regulation of circadian rhythm | biological_process | 1 | 148 | 64 | *Seita.1G170200* |
| GO:0016413 | O-acetyltransferase activity | molecular_function | 1 | 148 | 66 | *Seita.1G336700* |
| GO:0000977 | RNA polymerase II regulatory region sequence-specific DNA binding | molecular_function | 1 | 148 | 67 | *Seita.4G093200* |
| GO:0009809 | lignin biosynthetic process | biological_process | 1 | 148 | 70 | *Seita.8G153700* |
| GO:0042973 | glucan endo-1,3-beta-D-glucosidase activity | molecular_function | 1 | 148 | 72 | *Seita.7G090400* |
| GO:0008134 | transcription factor binding | molecular_function | 1 | 148 | 73 | *Seita.4G093200* |
| GO:0009860 | pollen tube growth | biological_process | 1 | 148 | 73 | *Seita.2G418500* |
| GO:0003333 | amino acid transmembrane transport | biological_process | 1 | 148 | 75 | *Seita.3G024600* |
| GO:0015035 | protein disulfide oxidoreductase activity | molecular_function | 1 | 148 | 75 | *Seita.3G089400* |
| GO:0061630 | ubiquitin protein ligase activity | molecular_function | 2 | 148 | 197 | *Seita.1G197500;Seita.3G066800* |
| GO:0005096 | GTPase activator activity | molecular_function | 1 | 148 | 79 | *Seita.6G128200* |
| GO:0015171 | amino acid transmembrane transporter activity | molecular_function | 1 | 148 | 81 | *Seita.3G024600* |
| GO:0015333 | peptide:proton symporter activity | molecular_function | 1 | 148 | 84 | *Seita.5G326900* |
| GO:1904680 | peptide transmembrane transporter activity | molecular_function | 1 | 148 | 84 | *Seita.5G326900* |
| GO:0043161 | proteasome-mediated ubiquitin-dependent protein catabolic process | biological_process | 1 | 148 | 85 | *Seita.8G143300* |
| GO:0009813 | flavonoid biosynthetic process | biological_process | 1 | 148 | 87 | *Seita.9G142900* |
| GO:0004497 | monooxygenase activity | molecular_function | 2 | 148 | 216 | *Seita.5G009900;Seita.7G206600* |
| GO:0042802 | identical protein binding | molecular_function | 2 | 148 | 217 | *Seita.7G239900;Seita.9G142900* |
| GO:0050660 | flavin adenine dinucleotide binding | molecular_function | 1 | 148 | 90 | *Seita.9G224200* |
| GO:0004722 | protein serine/threonine phosphatase activity | molecular_function | 1 | 148 | 91 | *Seita.9G082200* |
| GO:0035673 | oligopeptide transmembrane transporter activity | molecular_function | 1 | 148 | 91 | *Seita.5G326900* |
| GO:0009909 | regulation of flower development | biological_process | 1 | 148 | 95 | *Seita.4G093200* |
| GO:0016757 | transferase activity, transferring glycosyl groups | molecular_function | 1 | 148 | 97 | *Seita.5G331000* |
| GO:0051213 | dioxygenase activity | molecular_function | 1 | 148 | 101 | *Seita.3G148100* |
| GO:0009870 | defense response signaling pathway, resistance gene-dependent | biological_process | 1 | 148 | 105 | *Seita.7G094400* |
| GO:0006355 | regulation of transcription, DNA-templated | biological_process | 6 | 148 | 792 | *Seita.1G290700;Seita.2G086600;Seita.2G140900;Seita.6G023700;Seita.6G023800;Seita.7G112000* |
| GO:0009555 | pollen development | biological_process | 1 | 148 | 121 | *Seita.4G093200* |
| GO:0009734 | auxin-activated signaling pathway | biological_process | 2 | 148 | 267 | *Seita.9G327900;Seita.9G328000* |
| GO:0007275 | multicellular organism development | biological_process | 3 | 148 | 411 | *Seita.4G093200;Seita.6G170000;Seita.6G170200* |
| GO:0046777 | protein autophosphorylation | biological_process | 1 | 148 | 125 | *Seita.7G239900* |
| GO:0004672 | protein kinase activity | molecular_function | 1 | 148 | 127 | *Seita.7G239900* |
| GO:0045944 | positive regulation of transcription by RNA polymerase II | biological_process | 1 | 148 | 127 | *Seita.4G093200* |
| GO:0009651 | response to salt stress | biological_process | 3 | 148 | 420 | *Seita.1G062100;Seita.2G140900;Seita.8G153700* |
| GO:0046872 | metal ion binding | molecular_function | 18 | 148 | 2504 | *Seita.1G170200;Seita.1G186400;Seita.1G197500;Seita.1G290700;Seita.1G327300;Seita.2G225500;Seita.3G004800;Seita.3G037900;Seita.3G066800;Seita.3G148100;Seita.5G299000;Seita.7G128200;Seita.8G033400;Seita.8G143300;Seita.9G080500;Seita.9G082200;Seita.9G153500;Seita.9G169700* |
| GO:0045454 | cell redox homeostasis | biological_process | 1 | 148 | 132 | *Seita.3G089400* |
| GO:0055114 | oxidation-reduction process | biological_process | 1 | 148 | 143 | *Seita.9G224200* |
| GO:0000287 | magnesium ion binding | molecular_function | 1 | 148 | 145 | *Seita.6G048500* |
| GO:0010150 | leaf senescence | biological_process | 1 | 148 | 145 | *Seita.9G069700* |
| GO:0009416 | response to light stimulus | biological_process | 1 | 148 | 152 | *Seita.4G225400* |
| GO:0005516 | calmodulin binding | molecular_function | 1 | 148 | 164 | *Seita.3G163300* |
| GO:0005507 | copper ion binding | molecular_function | 1 | 148 | 175 | *Seita.9G537500* |
| GO:0046686 | response to cadmium ion | biological_process | 1 | 148 | 179 | *Seita.2G140900* |
| GO:0016740 | transferase activity | molecular_function | 1 | 148 | 183 | *Seita.9G153500* |
| GO:0003924 | GTPase activity | molecular_function | 1 | 148 | 186 | *Seita.5G381400* |
| GO:0016567 | protein ubiquitination | biological_process | 4 | 148 | 680 | *Seita.1G197500;Seita.1G327300;Seita.3G066800;Seita.9G153500* |
| GO:0030154 | cell differentiation | biological_process | 1 | 148 | 194 | *Seita.1G239400* |
| GO:0040008 | regulation of growth | biological_process | 1 | 148 | 194 | *Seita.5G346700* |
| GO:0043531 | ADP binding | molecular_function | 2 | 148 | 379 | *Seita.6G017500;Seita.8G042800* |
| GO:0006886 | intracellular protein transport | biological_process | 1 | 148 | 217 | *Seita.6G128200* |
| GO:0006511 | ubiquitin-dependent protein catabolic process | biological_process | 1 | 148 | 241 | *Seita.1G197500* |
| GO:0051301 | cell division | biological_process | 1 | 148 | 253 | *Seita.1G197500* |
| GO:0005525 | GTP binding | molecular_function | 1 | 148 | 273 | *Seita.5G381400* |
| GO:0005737 | cytoplasm | cellular_component | 14 | 148 | 2424 | *Seita.3G066800;Seita.3G089400;Seita.3G138700;Seita.3G163300;Seita.4G227700;Seita.5G363900;Seita.5G377300;Seita.6G048500;Seita.6G093100;Seita.6G128200;Seita.6G170000;Seita.6G170200;Seita.7G090400;Seita.7G227400* |
| GO:0046983 | protein dimerization activity | molecular_function | 1 | 148 | 312 | *Seita.4G093200* |
| GO:0005773 | vacuole | cellular_component | 1 | 148 | 408 | *Seita.2G038200* |
| GO:0005730 | nucleolus | cellular_component | 1 | 148 | 409 | *Seita.4G227700* |
| GO:0005774 | vacuolar membrane | cellular_component | 1 | 148 | 420 | *Seita.3G008300* |
| GO:0005783 | endoplasmic reticulum | cellular_component | 2 | 148 | 725 | *Seita.2G069800;Seita.5G378700* |
| GO:0004674 | protein serine/threonine kinase activity | molecular_function | 1 | 148 | 544 | *Seita.7G239900* |
| GO:0008270 | zinc ion binding | molecular_function | 1 | 148 | 552 | *Seita.2G195200* |
| GO:0005789 | endoplasmic reticulum membrane | cellular_component | 1 | 148 | 594 | *Seita.7G177500* |
| GO:0005829 | cytosol | cellular_component | 6 | 148 | 1737 | *Seita.2G418500;Seita.3G066800;Seita.3G138700;Seita.6G093100;Seita.6G128200;Seita.6G170300* |
| GO:0003723 | RNA binding | molecular_function | 1 | 148 | 654 | *Seita.6G159400* |
| GO:0005524 | ATP binding | molecular_function | 10 | 148 | 2580 | *Seita.2G069800;Seita.2G418500;Seita.3G047600;Seita.3G138700;Seita.6G017500;Seita.6G093100;Seita.7G239900;Seita.7G248700;Seita.8G042800;Seita.9G190200* |
| GO:0009507 | chloroplast | cellular_component | 2 | 148 | 1615 | *Seita.3G048400;Seita.7G296300* |
|  |  |  |  |  |  |  |

| Table S7. KEGG enrichment analysis of differentially expressed genes in foxtail millet after 3 days of PET nanoplastics treatment | | | | | | | | | | | |
| --- | --- | --- | --- | --- | --- | --- | --- | --- | --- | --- | --- |
| id | Term | Classification_level1 | Classification_level2 | ListHits | ListTotal | PopHits | PopTotal | p-value | q-value | Enrichment_score | geneID |
| ko04144 | Endocytosis | Cellular Processes | Transport and catabolism | 4 | 93 | 174 | 5633 | 0.32350323 | 0.623581956 | 1.392411321 | *Seita.3G216900;Seita.5G067800;Seita.5G376100;Seita.9G451900* |
| ko04145 | Phagosome | Cellular Processes | Transport and catabolism | 2 | 93 | 78 | 5633 | 0.370361076 | 0.623581956 | 1.553074166 | *Seita.2G200700;Seita.5G017200* |
| ko04146 | Peroxisome | Cellular Processes | Transport and catabolism | 1 | 93 | 109 | 5633 | 0.839965573 | 0.872271941 | 0.555687087 | *Seita.1G117500* |
| ko04016 | MAPK signaling pathway - plant | Environmental Information Processing | Signal transduction | 12 | 93 | 172 | 5633 | 2.17E-05 | 0.000607535 | 4.225806452 | *Seita.1G117500;Seita.2G177500;Seita.3G076200;Seita.3G139000;Seita.3G160800;Seita.3G196600;Seita.3G218800;Seita.5G233100;Seita.5G284300;Seita.5G379400;Seita.9G427100;Seita.9G437300* |
| ko04070 | Phosphatidylinositol signaling system | Environmental Information Processing | Signal transduction | 4 | 93 | 71 | 5633 | 0.029202337 | 0.210748369 | 3.412388308 | *Seita.2G200700;Seita.3G048500;Seita.3G196600;Seita.9G427100* |
| ko04075 | Plant hormone signal transduction | Environmental Information Processing | Signal transduction | 14 | 93 | 273 | 5633 | 0.000132525 | 0.002385453 | 3.106148332 | *Seita.1G331600;Seita.1G356800;Seita.2G177500;Seita.2G217500;Seita.3G055700;Seita.3G076200;Seita.3G139000;Seita.3G218800;Seita.3G379500;Seita.5G312700;Seita.5G379400;Seita.7G230900;Seita.9G067200;Seita.9G437300* |
| ko03040 | Spliceosome | Genetic Information Processing | Transcription | 3 | 93 | 199 | 5633 | 0.644452519 | 0.774933826 | 0.913113957 | *Seita.3G216900;Seita.5G376100;Seita.9G451900* |
| ko04120 | Ubiquitin mediated proteolysis | Genetic Information Processing | Folding, sorting and degradation | 1 | 93 | 152 | 5633 | 0.923101986 | 0.940519005 | 0.398486135 | *Seita.9G234200* |
| ko04130 | SNARE interactions in vesicular transport | Genetic Information Processing | Folding, sorting and degradation | 1 | 93 | 37 | 5633 | 0.46095533 | 0.691432995 | 1.637024121 | *Seita.7G077700* |
| ko04141 | Protein processing in endoplasmic reticulum | Genetic Information Processing | Folding, sorting and degradation | 8 | 93 | 219 | 5633 | 0.027466661 | 0.210748369 | 2.212598812 | *Seita.1G326600;Seita.1G342400;Seita.3G216900;Seita.5G092600;Seita.5G376100;Seita.6G200300;Seita.9G451900;Seita.9G461500* |
| ko04933 | AGE-RAGE signaling pathway in diabetic complications | Human Diseases | Endocrine and metabolic disease | 1 | 93 | 10 | 5633 | 0.153471025 | 0.623581956 | 6.056989247 | *Seita.3G048500* |
| ko00030 | Pentose phosphate pathway | Metabolism | Carbohydrate metabolism | 1 | 93 | 62 | 5633 | 0.645778189 | 0.774933826 | 0.97693375 | *Seita.9G365500* |
| ko00040 | Pentose and glucuronate interconversions | Metabolism | Carbohydrate metabolism | 1 | 93 | 87 | 5633 | 0.767673463 | 0.82908734 | 0.696205661 | *Seita.6G235000* |
| ko00051 | Fructose and mannose metabolism | Metabolism | Carbohydrate metabolism | 2 | 93 | 67 | 5633 | 0.30373746 | 0.623581956 | 1.808056492 | *Seita.5G260400;Seita.6G235000* |
| ko00052 | Galactose metabolism | Metabolism | Carbohydrate metabolism | 2 | 93 | 65 | 5633 | 0.29145951 | 0.623581956 | 1.863688999 | *Seita.2G282600;Seita.6G195300* |
| ko00053 | Ascorbate and aldarate metabolism | Metabolism | Carbohydrate metabolism | 1 | 93 | 59 | 5633 | 0.627436115 | 0.774933826 | 1.026608347 | *Seita.6G210000* |
| ko00061 | Fatty acid biosynthesis | Metabolism | Lipid metabolism | 2 | 93 | 57 | 5633 | 0.242236266 | 0.623581956 | 2.125259385 | *Seita.1G349400;Seita.4G031400* |
| ko00062 | Fatty acid elongation | Metabolism | Lipid metabolism | 2 | 93 | 51 | 5633 | 0.205596521 | 0.623581956 | 2.375289901 | *Seita.1G362000;Seita.4G225400* |
| ko00100 | Steroid biosynthesis | Metabolism | Lipid metabolism | 1 | 93 | 42 | 5633 | 0.504298003 | 0.707127546 | 1.442140297 | *Seita.5G327000* |
| ko00190 | Oxidative phosphorylation | Metabolism | Energy metabolism | 1 | 93 | 176 | 5633 | 0.949047747 | 0.949047747 | 0.344147116 | *Seita.5G017200* |
| ko00230 | Purine metabolism | Metabolism | Nucleotide metabolism | 1 | 93 | 108 | 5633 | 0.837225661 | 0.872271941 | 0.560832338 | *Seita.1G231800* |
| ko00240 | Pyrimidine metabolism | Metabolism | Nucleotide metabolism | 1 | 93 | 73 | 5633 | 0.705706967 | 0.825802299 | 0.829724554 | *Seita.3G138700* |
| ko00270 | Cysteine and methionine metabolism | Metabolism | Amino acid metabolism | 2 | 93 | 122 | 5633 | 0.603053127 | 0.774933826 | 0.992949057 | *Seita.3G037900;Seita.4G075200* |
| ko00280 | Valine, leucine and isoleucine degradation | Metabolism | Amino acid metabolism | 1 | 93 | 56 | 5633 | 0.608154998 | 0.774933826 | 1.081605223 | *Seita.3G382100* |
| ko00330 | Arginine and proline metabolism | Metabolism | Amino acid metabolism | 2 | 93 | 56 | 5633 | 0.236098704 | 0.623581956 | 2.163210445 | *Seita.3G020400;Seita.3G020600* |
| ko00380 | Tryptophan metabolism | Metabolism | Amino acid metabolism | 1 | 93 | 77 | 5633 | 0.724913591 | 0.825802299 | 0.78662198 | *Seita.1G117500* |
| ko00410 | beta-Alanine metabolism | Metabolism | Metabolism of other amino acids | 2 | 93 | 47 | 5633 | 0.181549963 | 0.623581956 | 2.577442233 | *Seita.3G020400;Seita.3G020600* |
| ko00440 | Phosphonate and phosphinate metabolism | Metabolism | Metabolism of other amino acids | 1 | 93 | 8 | 5633 | 0.124766693 | 0.612491037 | 7.571236559 | *Seita.9G306400* |
| ko00460 | Cyanoamino acid metabolism | Metabolism | Metabolism of other amino acids | 2 | 93 | 76 | 5633 | 0.358400948 | 0.623581956 | 1.593944539 | *Seita.2G415300;Seita.4G134400* |
| ko00480 | Glutathione metabolism | Metabolism | Metabolism of other amino acids | 3 | 93 | 139 | 5633 | 0.40417349 | 0.623581956 | 1.307263866 | *Seita.6G210000;Seita.9G064500;Seita.9G365500* |
| ko00500 | Starch and sucrose metabolism | Metabolism | Carbohydrate metabolism | 8 | 93 | 180 | 5633 | 0.0093592 | 0.101079355 | 2.691995221 | *Seita.1G262000;Seita.1G347300;Seita.2G197800;Seita.2G415300;Seita.4G134400;Seita.5G318400;Seita.6G144600;Seita.9G229700* |
| ko00520 | Amino sugar and nucleotide sugar metabolism | Metabolism | Carbohydrate metabolism | 2 | 93 | 164 | 5633 | 0.759798183 | 0.82908734 | 0.738657225 | *Seita.1G225300;Seita.2G282600* |
| ko00561 | Glycerolipid metabolism | Metabolism | Lipid metabolism | 2 | 93 | 103 | 5633 | 0.510703228 | 0.707127546 | 1.176114417 | *Seita.5G327000;Seita.6G110100* |
| ko00562 | Inositol phosphate metabolism | Metabolism | Carbohydrate metabolism | 2 | 93 | 69 | 5633 | 0.315979986 | 0.623581956 | 1.755649057 | *Seita.2G200700;Seita.3G048500* |
| ko00564 | Glycerophospholipid metabolism | Metabolism | Lipid metabolism | 3 | 93 | 131 | 5633 | 0.368111548 | 0.623581956 | 1.387096774 | *Seita.1G171900;Seita.5G327000;Seita.9G306400* |
| ko00565 | Ether lipid metabolism | Metabolism | Lipid metabolism | 1 | 93 | 30 | 5633 | 0.393912375 | 0.623581956 | 2.018996416 | *Seita.5G327000* |
| ko00590 | Arachidonic acid metabolism | Metabolism | Lipid metabolism | 1 | 93 | 16 | 5633 | 0.234113141 | 0.623581956 | 3.78561828 | *Seita.5G327000* |
| ko00591 | Linoleic acid metabolism | Metabolism | Lipid metabolism | 1 | 93 | 16 | 5633 | 0.234113141 | 0.623581956 | 3.78561828 | *Seita.5G327000* |
| ko00592 | alpha-Linolenic acid metabolism | Metabolism | Lipid metabolism | 2 | 93 | 59 | 5633 | 0.254532424 | 0.623581956 | 2.053216694 | *Seita.5G169500;Seita.5G327000* |
| ko00630 | Glyoxylate and dicarboxylate metabolism | Metabolism | Carbohydrate metabolism | 1 | 93 | 79 | 5633 | 0.734046488 | 0.825802299 | 0.7667075 | *Seita.1G117500* |
| ko00740 | Riboflavin metabolism | Metabolism | Metabolism of cofactors and vitamins | 1 | 93 | 20 | 5633 | 0.28360295 | 0.623581956 | 3.028494624 | *Seita.3G216400* |
| ko00780 | Biotin metabolism | Metabolism | Metabolism of cofactors and vitamins | 1 | 93 | 26 | 5633 | 0.35196504 | 0.623581956 | 2.329611249 | *Seita.1G349400* |
| ko00790 | Folate biosynthesis | Metabolism | Metabolism of cofactors and vitamins | 1 | 93 | 29 | 5633 | 0.38368444 | 0.623581956 | 2.088616982 | *Seita.3G216400* |
| ko00860 | Porphyrin and chlorophyll metabolism | Metabolism | Metabolism of cofactors and vitamins | 1 | 93 | 52 | 5633 | 0.580903623 | 0.774933826 | 1.164805624 | *Seita.2G134400* |
| ko00902 | Monoterpenoid biosynthesis | Metabolism | Metabolism of terpenoids and polyketides | 2 | 93 | 17 | 5633 | 0.031221981 | 0.210748369 | 7.125869703 | *Seita.7G179400;Seita.9G042400* |
| ko00904 | Diterpenoid biosynthesis | Metabolism | Metabolism of terpenoids and polyketides | 1 | 93 | 30 | 5633 | 0.393912375 | 0.623581956 | 2.018996416 | *Seita.5G147400* |
| ko00905 | Brassinosteroid biosynthesis | Metabolism | Metabolism of terpenoids and polyketides | 1 | 93 | 17 | 5633 | 0.246793838 | 0.623581956 | 3.562934851 | *Seita.8G027000* |
| ko00906 | Carotenoid biosynthesis | Metabolism | Metabolism of terpenoids and polyketides | 6 | 93 | 36 | 5633 | 2.25E-05 | 0.000607535 | 10.09498208 | *Seita.1G288100;Seita.1G288400;Seita.2G035400;Seita.2G229900;Seita.7G209000;Seita.9G156500* |
| ko00908 | Zeatin biosynthesis | Metabolism | Metabolism of terpenoids and polyketides | 2 | 93 | 40 | 5633 | 0.140743911 | 0.623581956 | 3.028494624 | *Seita.6G174400;Seita.9G217200* |
| ko00910 | Nitrogen metabolism | Metabolism | Energy metabolism | 1 | 93 | 39 | 5633 | 0.478724806 | 0.698679447 | 1.553074166 | *Seita.6G189200* |
| ko00920 | Sulfur metabolism | Metabolism | Energy metabolism | 2 | 93 | 37 | 5633 | 0.123954474 | 0.612491037 | 3.274048242 | *Seita.2G327500;Seita.4G075200* |
| ko00940 | Phenylpropanoid biosynthesis | Metabolism | Biosynthesis of other secondary metabolites | 7 | 93 | 274 | 5633 | 0.16551953 | 0.623581956 | 1.547406012 | *Seita.1G022500;Seita.2G415300;Seita.4G134400;Seita.4G176600;Seita.9G298200;Seita.9G562600;Seita.J003100* |
| ko04626 | Plant-pathogen interaction | Organismal Systems | Environmental adaptation | 8 | 93 | 253 | 5633 | 0.056464821 | 0.338788926 | 1.915253517 | *Seita.1G362000;Seita.3G196600;Seita.3G261400;Seita.4G225400;Seita.5G233100;Seita.6G198600;Seita.6G200300;Seita.9G427100* |
| ko04712 | Circadian rhythm - plant | Organismal Systems | Environmental adaptation | 4 | 93 | 45 | 5633 | 0.006186439 | 0.083516923 | 5.383990442 | *Seita.1G043800;Seita.1G236100;Seita.1G376200;Seita.3G055700* |
|  |  |  |  |  |  |  |  |  |  |  |  |
|  | | | | | | | | | | | |

| Table S8. KEGG enrichment analysis of differentially expressed genes in foxtail millet after 7 days of PET nanoplastics treatment | | | | | | | | | | | |
| --- | --- | --- | --- | --- | --- | --- | --- | --- | --- | --- | --- |
| id | Term | Classification_level1 | Classification_level2 | ListHits | ListTotal | PopHits | PopTotal | p-value | q-value | Enrichment_score | geneID |
| ko04144 | Endocytosis | Cellular Processes | Transport and catabolism | 2 | 28 | 174 | 5633 | 0.213737244 | 0.388332901 | 2.312397373 | *Seita.2G418500;Seita.5G381400* |
| ko04075 | Plant hormone signal transduction | Environmental Information Processing | Signal transduction | 3 | 28 | 273 | 5633 | 0.151900464 | 0.360763602 | 2.210753532 | *Seita.4G246400;Seita.7G227400;Seita.9G358800* |
| ko04016 | MAPK signaling pathway - plant | Environmental Information Processing | Signal transduction | 3 | 28 | 172 | 5633 | 0.052418855 | 0.331986079 | 3.508928571 | *Seita.3G206900;Seita.7G094400;Seita.7G239900* |
| ko04070 | Phosphatidylinositol signaling system | Environmental Information Processing | Signal transduction | 1 | 28 | 71 | 5633 | 0.299543304 | 0.388332901 | 2.833501006 | *Seita.2G418500* |
| ko00970 | Aminoacyl-tRNA biosynthesis | Genetic Information Processing | Translation | 1 | 28 | 72 | 5633 | 0.303069515 | 0.388332901 | 2.794146825 | *Seita.6G093100* |
| ko00940 | Phenylpropanoid biosynthesis | Metabolism | Biosynthesis of other secondary metabolites | 2 | 28 | 274 | 5633 | 0.398377691 | 0.445245654 | 1.468456726 | *Seita.3G004800;Seita.7G128200* |
| ko00500 | Starch and sucrose metabolism | Metabolism | Carbohydrate metabolism | 1 | 28 | 180 | 5633 | 0.598102125 | 0.598102125 | 1.11765873 | *Seita.2G188200* |
| ko00010 | Glycolysis / Gluconeogenesis | Metabolism | Carbohydrate metabolism | 2 | 28 | 164 | 5633 | 0.19546015 | 0.388332901 | 2.453397213 | *Seita.5G251100;Seita.5G377300* |
| ko00520 | Amino sugar and nucleotide sugar metabolism | Metabolism | Carbohydrate metabolism | 1 | 28 | 164 | 5633 | 0.563652993 | 0.594967049 | 1.226698606 | *Seita.8G008500* |
| ko00270 | Cysteine and methionine metabolism | Metabolism | Amino acid metabolism | 2 | 28 | 122 | 5633 | 0.122223911 | 0.360763602 | 3.298009368 | *Seita.2G225500;Seita.3G037900* |
| ko00710 | Carbon fixation in photosynthetic organisms | Metabolism | Energy metabolism | 1 | 28 | 83 | 5633 | 0.340745316 | 0.404635063 | 2.42383821 | *Seita.5G377300* |
| ko00240 | Pyrimidine metabolism | Metabolism | Nucleotide metabolism | 1 | 28 | 73 | 5633 | 0.306578606 | 0.388332901 | 2.755870841 | *Seita.3G138700* |
| ko00562 | Inositol phosphate metabolism | Metabolism | Carbohydrate metabolism | 2 | 28 | 69 | 5633 | 0.045553253 | 0.331986079 | 5.83126294 | *Seita.2G418500;Seita.5G377300* |
| ko00051 | Fructose and mannose metabolism | Metabolism | Carbohydrate metabolism | 1 | 28 | 67 | 5633 | 0.285265638 | 0.388332901 | 3.002665245 | *Seita.5G377300* |
| ko00062 | Fatty acid elongation | Metabolism | Lipid metabolism | 1 | 28 | 51 | 5633 | 0.225297201 | 0.388332901 | 3.944677871 | *Seita.4G225400* |
| ko00600 | Sphingolipid metabolism | Metabolism | Lipid metabolism | 1 | 28 | 32 | 5633 | 0.147771699 | 0.360763602 | 6.286830357 | *Seita.7G177500* |
| ko00904 | Diterpenoid biosynthesis | Metabolism | Metabolism of terpenoids and polyketides | 1 | 28 | 30 | 5633 | 0.139188931 | 0.360763602 | 6.705952381 | *Seita.3G148100* |
| ko00909 | Sesquiterpenoid and triterpenoid biosynthesis | Metabolism | Metabolism of terpenoids and polyketides | 1 | 28 | 15 | 5633 | 7.21E-02 | 0.342512545 | 13.41190476 | *Seita.6G048500* |
| ko04626 | Plant-pathogen interaction | Organismal Systems | Environmental adaptation | 9 | 28 | 253 | 5633 | 2.10441E-06 | 3.99837E-05 | 7.156549972 | *Seita.3G135500;Seita.3G206900;Seita.3G261400;Seita.3G380800;Seita.4G225400;Seita.5G337400;Seita.5G451500;Seita.5G456800;Seita.7G239900* |
|  |  |  |  |  |  |  |  |  |  |  |  |

| Table S9. KEGG pathway analysis of the unique DEGs in the turquoise module | | | | | | | |
| --- | --- | --- | --- | --- | --- | --- | --- |
| id | Term | Classification_level1 | Classification_level2 | ListHits | p-value | Enrichment_score | geneID |
| ko04141 | Protein processing in endoplasmic reticulum | Genetic Information Processing | Folding, sorting and degradation | 6 | 0.00258251788775955 | 4.20729684908789 | *Seita.1G326600;Seita.1G342400;Seita.5G092600;Seita.5G376100;Seita.6G200300;Seita.9G451900* |
| ko04016 | MAPK signaling pathway - plant | Environmental Information Processing | Signal transduction | 5 | 0.00449379441081606 | 4.51745014245014 | *Seita.3G139000;Seita.3G218800;Seita.5G284300;Seita.5G379400;Seita.9G427100* |
| ko00904 | Diterpenoid biosynthesis | Metabolism | Metabolism of terpenoids and polyketides | 2 | 0.0187970197188779 | 9.3962962962963 | *Seita.3G148100;Seita.5G147400* |
| ko00920 | Sulfur metabolism | Metabolism | Energy metabolism | 2 | 0.0238165977742034 | 8.29084967320261 | *Seita.2G327500;Seita.4G075200* |
| ko00906 | Carotenoid biosynthesis | Metabolism | Metabolism of terpenoids and polyketides | 2 | 0.0251486830076147 | 8.05396825396825 | *Seita.2G035400;Seita.7G209000* |
| ko04075 | Plant hormone signal transduction | Environmental Information Processing | Signal transduction | 5 | 0.0338916405892986 | 2.73148148148148 | *Seita.1G331600;Seita.3G055700;Seita.3G139000;Seita.3G218800;Seita.5G379400* |
| ko04712 | Circadian rhythm - plant | Organismal Systems | Environmental adaptation | 2 | 0.0384291751185895 | 6.40656565656566 | *Seita.1G043800;Seita.3G055700* |
| ko00440 | Phosphonate and phosphinate metabolism | Metabolism | Metabolism of other amino acids | 1 | 0.055407571669843 | 17.6180555555556 | *Seita.9G306400* |
| ko04070 | Phosphatidylinositol signaling system | Environmental Information Processing | Signal transduction | 2 | 0.0812185255849148 | 4.20729684908789 | *Seita.2G200700;Seita.9G427100* |
| ko00902 | Monoterpenoid biosynthesis | Metabolism | Metabolism of terpenoids and polyketides | 1 | 0.0885091384728663 | 10.8418803418803 | *Seita.9G042400* |
| ko04814 | Motor proteins | Cellular Processes | Cell motility | 2 | 0.118959708339472 | 3.35582010582011 | *Seita.2G352400;Seita.7G248700* |
| ko04626 | Plant-pathogen interaction | Organismal Systems | Environmental adaptation | 3 | 0.190776154496146 | 1.98513302034429 | *Seita.3G261400;Seita.6G200300;Seita.9G427100* |
| ko00564 | Glycerophospholipid metabolism | Metabolism | Lipid metabolism | 2 | 0.193300348575047 | 2.47270955165692 | *Seita.1G171900;Seita.9G306400* |
| ko00908 | Zeatin biosynthesis | Metabolism | Metabolism of terpenoids and polyketides | 1 | 0.237814807527845 | 3.70906432748538 | *Seita.6G174400* |
| ko00061 | Fatty acid biosynthesis | Metabolism | Lipid metabolism | 1 | 0.310738750351295 | 2.71047008547009 | *Seita.4G031400* |
| ko04144 | Endocytosis | Cellular Processes | Transport and catabolism | 2 | 0.312096829448061 | 1.77288609364081 | *Seita.5G376100;Seita.9G451900* |
| ko00592 | alpha-Linolenic acid metabolism | Metabolism | Lipid metabolism | 1 | 0.315679691208992 | 2.65932914046122 | *Seita.5G169500* |
| ko00562 | Inositol phosphate metabolism | Metabolism | Carbohydrate metabolism | 1 | 0.358625945088103 | 2.27329749103943 | *Seita.2G200700* |
| ko00240 | Pyrimidine metabolism | Metabolism | Nucleotide metabolism | 1 | 0.372350142612781 | 2.16837606837607 | *Seita.3G138700* |
| ko03040 | Spliceosome | Genetic Information Processing | Transcription | 2 | 0.385145886108718 | 1.50742721330957 | *Seita.5G376100;Seita.9G451900* |

| Table S10. KEGG pathway analysis of the unique DEGs in the red module | | | | | | | |
| --- | --- | --- | --- | --- | --- | --- | --- |
| id | Term | Classification_level1 | Classification_level2 | ListHits | p-value | Enrichment_score | geneID |
| ko04016 | MAPK signaling pathway - plant | Environmental Information Processing | Signal transduction | 6 | 0.00107985843767447 | 5.00394477317554 | *Seita.1G117500;Seita.3G076200;Seita.3G196600;Seita.5G233100;Seita.7G094400;Seita.9G437300* |
| ko04626 | Plant-pathogen interaction | Organismal Systems | Environmental adaptation | 6 | 0.00520965274492668 | 3.66486096063561 | *Seita.1G362000;Seita.3G135500;Seita.3G196600;Seita.3G380800;Seita.5G233100;Seita.6G198600* |
| ko00906 | Carotenoid biosynthesis | Metabolism | Metabolism of terpenoids and polyketides | 2 | 0.0292018862762583 | 7.43443223443223 | *Seita.1G288100;Seita.2G229900* |
| ko00770 | Pantothenate and CoA biosynthesis | Metabolism | Metabolism of cofactors and vitamins | 2 | 0.034009581667825 | 6.84750337381916 | *Seita.3G020400;Seita.3G020600* |
| ko00410 | beta-Alanine metabolism | Metabolism | Metabolism of other amino acids | 2 | 0.0444702644571786 | 5.91375291375291 | *Seita.3G020400;Seita.3G020600* |
| ko04712 | Circadian rhythm - plant | Organismal Systems | Environmental adaptation | 2 | 0.0444702644571786 | 5.91375291375291 | *Seita.1G236100;Seita.1G376200* |
| ko00330 | Arginine and proline metabolism | Metabolism | Amino acid metabolism | 2 | 0.0600010426621473 | 5.00394477317554 | *Seita.3G020400;Seita.3G020600* |
| ko00590 | Arachidonic acid metabolism | Metabolism | Lipid metabolism | 1 | 0.0743213470254009 | 13.0102564102564 | *Seita.5G327000* |
| ko00940 | Phenylpropanoid biosynthesis | Metabolism | Biosynthesis of other secondary metabolites | 4 | 0.0748488650393712 | 2.49000122684333 | *Seita.1G022500;Seita.4G176600;Seita.7G128200;Seita.9G298200* |
| ko00051 | Fructose and mannose metabolism | Metabolism | Carbohydrate metabolism | 2 | 0.0748909396943316 | 4.41025641025641 | *Seita.5G377300;Seita.6G235000* |
| ko00052 | Galactose metabolism | Metabolism | Carbohydrate metabolism | 2 | 0.0748909396943316 | 4.41025641025641 | *Seita.2G282600;Seita.6G195300* |
| ko00562 | Inositol phosphate metabolism | Metabolism | Carbohydrate metabolism | 2 | 0.0816019197550804 | 4.19685690653433 | *Seita.3G048500;Seita.5G377300* |
| ko04070 | Phosphatidylinositol signaling system | Environmental Information Processing | Signal transduction | 2 | 0.0931827805562295 | 3.88365862992729 | *Seita.3G048500;Seita.3G196600* |
| ko00902 | Monoterpenoid biosynthesis | Metabolism | Metabolism of terpenoids and polyketides | 1 | 0.0955483437951334 | 10.0078895463511 | *Seita.7G179400* |
| ko00591 | Linoleic acid metabolism | Metabolism | Lipid metabolism | 1 | 0.116300779912636 | 8.13141025641026 | *Seita.5G327000* |
| ko00905 | Brassinosteroid biosynthesis | Metabolism | Metabolism of terpenoids and polyketides | 1 | 0.116300779912636 | 8.13141025641026 | *Seita.8G027000* |
| ko00500 | Starch and sucrose metabolism | Metabolism | Carbohydrate metabolism | 3 | 0.139210987750195 | 2.30951297223487 | *Seita.1G347300;Seita.5G318400;Seita.6G144600* |
| ko00740 | Riboflavin metabolism | Metabolism | Metabolism of cofactors and vitamins | 1 | 0.156423096585689 | 5.91375291375291 | *Seita.3G216400* |
| ko00790 | Folate biosynthesis | Metabolism | Metabolism of cofactors and vitamins | 1 | 0.188497611699571 | 4.81861348528015 | *Seita.3G216400* |
| ko00565 | Ether lipid metabolism | Metabolism | Lipid metabolism | 1 | 0.200991934995859 | 4.48629531388152 | *Seita.5G327000* |

| Table S11. The standard curve provided by the kit is used by the manufacturer with additional raw data | | | | | | | | | | | | | | | | | | | | | | | |
| --- | --- | --- | --- | --- | --- | --- | --- | --- | --- | --- | --- | --- | --- | --- | --- | --- | --- | --- | --- | --- | --- | --- | --- |
| hydrogen peroxide (H₂O₂) content standard curve | | | | | |  | trehalose-6-phosphate phosphatase (TPP)activity standard curve | | | | | | |  | trehalase (TRE) activity standard curve | | | | | | | | |
| Standard concentration（μmol/mL) | 0.2 | 0.4 | 0.6 | 0.8 | 1 |  | Standard concentration（μmol/mL) | 0.25 | 0.2 | 0.15 | 0.08 | 0.06 | 0.04 |  | Standard concentration（μmol/mL) | 2.2 | 1.1 | 0.55 | 0.4 | 0.33 | 0.22 | 0.11 | 0.05 |
| ΔA | 0.1375 | 0.3103 | 0.462 | 0.5967 | 0.7431 |  | ΔA | 0.9736 | 0.7726 | 0.5648 | 0.2985 | 0.2458 | 0.1498 |  | ΔA | 1.0547 | 0.5407 | 0.2757 | 0.1997 | 0.1637 | 0.0937 | 0.0407 | 0.0067 |
|  |  |  |  |  |  |  |  |  |  |  |  |  |  |  |  |  |  |  |  |  |  |  |  |
|  |  |  |  |  |  |  |  |  |  |  |  |  |  |  |  |  |  |  |  |  |  |  |  |
|  |  |  |  |  |  |  |  |  |  |  |  |  |  |  |  |  |  |  |  |  |  |  |  |
|  |  |  |  |  |  |  |  |  |  |  |  |  |  |  |  |  |  |  |  |  |  |  |  |
|  |  |  |  |  |  |  |  |  |  |  |  |  |  |  |  |  |  |  |  |  |  |  |  |
|  |  |  |  |  |  |  |  |  |  |  |  |  |  |  |  |  |  |  |  |  |  |  |  |
|  |  |  |  |  |  |  |  |  |  |  |  |  |  |  |  |  |  |  |  |  |  |  |  |
| 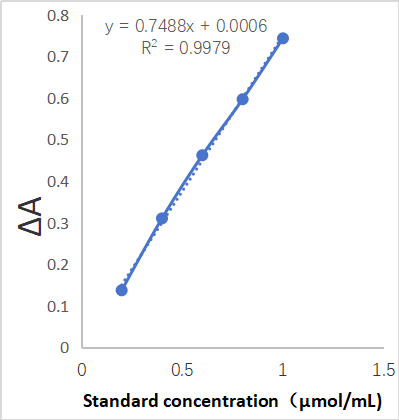 |  |  |  |  |  |  | 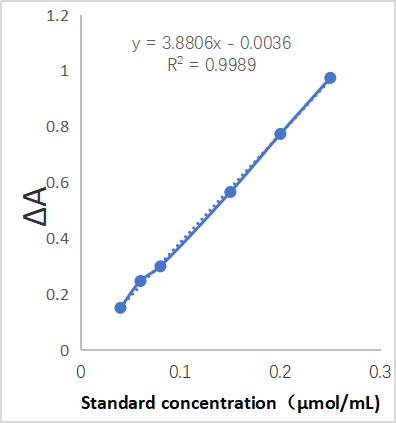 |  |  |  |  |  |  |  | 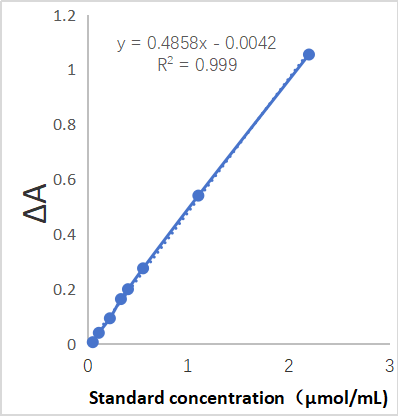 |  |  |  |  |  |  |  |  |
|  |  |  |  |  |  |  |  |  |  |  |  |  |  |  |  |  |  |  |  |  |  |  |  |
|  |  |  |  |  |  |  |  |  |  |  |  |  |  |  |  |  |  |  |  |  |  |  |  |
|  |  |  |  |  |  |  |  |  |  |  |  |  |  |  |  |  |  |  |  |  |  |  |  |
|  |  |  |  |  |  |  |  |  |  |  |  |  |  |  |  |  |  |  |  |  |  |  |  |
|  |  |  |  |  |  |  |  |  |  |  |  |  |  |  |  |  |  |  |  |  |  |  |  |
|  |  |  |  |  |  |  |  |  |  |  |  |  |  |  |  |  |  |  |  |  |  |  |  |
|  |  |  |  |  |  |  |  |  |  |  |  |  |  |  |  |  |  |  |  |  |  |  |  |
|  |  |  |  |  |  |  |  |  |  |  |  |  |  |  |  |  |  |  |  |  |  |  |  |
|  |  |  |  |  |  |  |  |  |  |  |  |  |  |  |  |  |  |  |  |  |  |  |  |
|  |  |  |  |  |  |  |  |  |  |  |  |  |  |  |  |  |  |  |  |  |  |  |  |
|  |  |  |  |  |  |  |  |  |  |  |  |  |  |  |  |  |  |  |  |  |  |  |  |
|  |  |  |  |  |  |  |  |  |  |  |  |  |  |  |  |  |  |  |  |  |  |  |  |
|  |  |  |  |  |  |  |  |  |  |  |  |  |  |  |  |  |  |  |  |  |  |  |  |
|  |  |  |  |  |  |  |  |  |  |  |  |  |  |  |  |  |  |  |  |  |  |  |  |
