# Supplementary material for: Transcriptome Analysis Reveals the Role of Trehalose in Response to Polyethylene Terephthalate Nanoplastics Treatment in Foxtail Millet ( Setaria italica ) Seedlings
Source: Food Sci Nutr. 2025 Jul 21;13(7):e70593. doi: 10.1002/fsn3.70593 (PMC12280233; doi:10.1002/fsn3.70593)
Supplement: Supplementary file 1 — Figure S1. Treatment with 1 g/L PET nanoplastics did not impact the growth of 2‐week‐old “Jingu21” foxtail millet seedlings. Figure S2. Cluster analysis among samples. The horizontal and vertical coordinates are the sample names, and the color represents the distance. The darker the color, the closer the distance is. [file FSN3-13-e70593-s001.pdf]

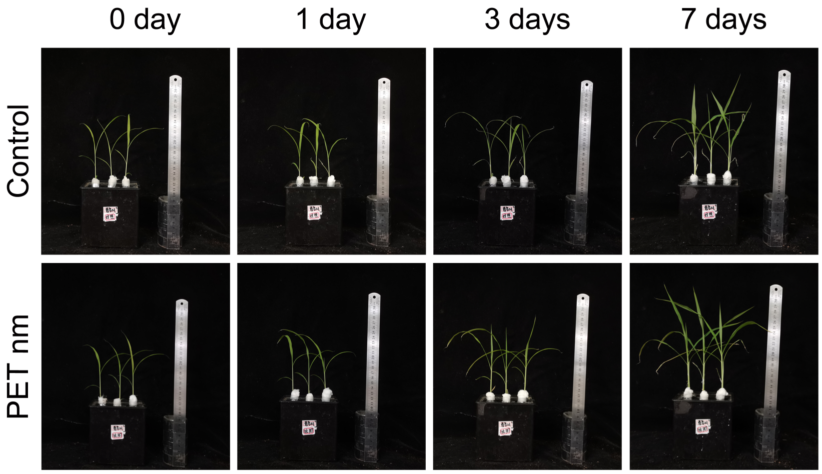

Figure S1 Treatment with 1 g/L PET nanoplastics did not impact the growth of two weeks old "Jingu21" foxtail millet seedlings.

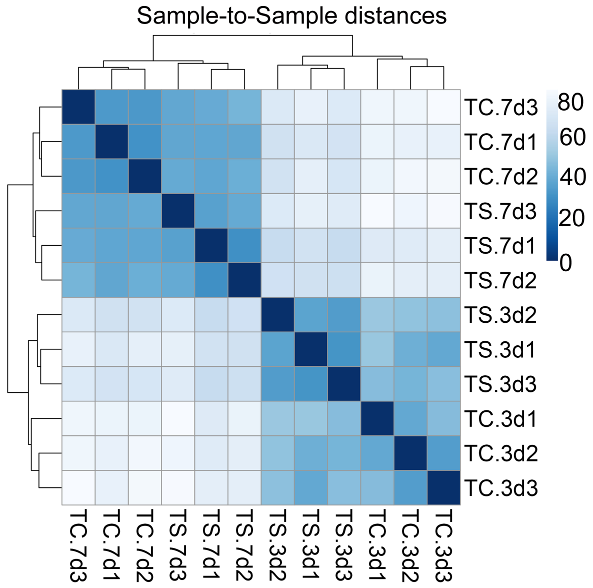

Figure S2 Cluster analysis among samples. The horizontal and vertical coordinates are the sample names, and the color represents the distance. The darker the color, the closer the distance is.

Table S1. Primer used for qRT-PCR.

Table S2. Transcriptome sequencing quality and reference genome alignment results.

Table S3. The differentially expressed genes in foxtail millet after 3 days of PET nanoplastics treatment.

Table S4. The differentially expressed genes in foxtail millet after 7 days of PET nanoplastics treatment.

Table S5. GO enrichment analysis of differentially expressed genes in foxtail millet after 3 days of PET nanoplastics treatment.

Table S6. GO enrichment analysis of differentially expressed genes in foxtail millet after 7 days of PET nanoplastics treatment.

Table S7. KEGG enrichment analysis of differentially expressed genes in foxtail millet after 3 days of PET nanoplastics treatment.

Table S8. KEGG enrichment analysis of differentially expressed genes in foxtail millet after 7 days of PET nanoplastics treatment.

Table S9. KEGG pathway analysis of the unique DEGs in the turquoise module.

Table S10. KEGG pathway analysis of the unique DEGs in the red module.

Table S11. The standard curve provided by the kit is used by the manufacturer with additional raw data.
